# MGP regulates the adipogenic differentiation of mesenchymal stem cells in osteoporosis via the Ca2+/CaMKII/RIP140/FABP3 axis

**DOI:** 10.1038/s41420-025-02472-2

**Published:** 2025-04-12

**Authors:** Xinglang Wang, Yunhui Zhang, Chenhao Xu, Quanfeng Li, Pengfei Ji, Huatao Liu, Yibin Zhang, Jiahao Jin, Zihao Yuan, Miao Yuan, Pei Feng, Yanfeng Wu, Wenjie Liu, Huiyong Shen, Peng Wang

**Affiliations:** 1 Department of Orthopedics, The Eighth Affiliated Hospital, Sun Yat-sen University, 3025# Shennan Road, Shenzhen, 518000 PR China; 2 Center for Biotherapy, The Eighth Affiliated Hospital, Sun Yat-sen University, 3025# Shennan Road, Shenzhen, 518000 PR China

**Keywords:** Mesenchymal stem cells, Transcriptional regulatory elements

## Abstract

The dysregulation of bone marrow mesenchymal stem cells (BM-MSCs) is crucial in the pathogenesis of osteoporosis, and adipogenic differentiation of BM-MSCs is considered an essential factor in this process. However, the mechanisms underlying the regulation of MSC adipogenic differentiation require further investigation. MGP (Matrix Gla Protein) was reported to impair the osteogenic differentiation. However, the mechanisms through which MGP regulates osteoporosis and bone-fat imbalance in MSCs are still unclear. In this study, we confirmed that the expression of MGP upregulated in osteoporosis and has a negative correlation with BMD (bone mineral density). Gain- and loss-of-function experiments were performed to ensure the role of MGP in MSC adipogenic differentiation. Mechanistically, MGP increased intracellular free Ca2+ levels and enhanced CaMKII phosphorylation, which in turn activated RIP140 protein degradation. This led to an increase in the transcription of FABP3, ultimately promoting adipogenic differentiation in MSCs. Furthermore, we demonstrated that using recombinant adeno-associated virus 9 (rAAV9) to silence MGP has the effect of alleviating bone loss and reversing the excessive bone marrow adipose tissue in mice with osteoporosis. In summary, our research has unveiled the regulatory role of MGP/Ca2+/CaMKII/RIP140/FABP3 axis in adipogenic differentiation in MSC and it might be a promising approach for osteoporosis treatment.

## Introduction

Osteoporosis is a systemic bone disease characterized by deterioration of the bone microstructure and loss of bone mass, which lead to increased bone fragility and an increased risk of fracture [1]. The incidence of this disease increases with age and changes in energy metabolism [2]. Studies have shown that osteoporosis is often accompanied by abnormal fat accumulation and is thus also called “bone obesity” [3]. This condition is caused by the abnormal differentiation of adipocytes and osteoblast progenitors [4]. However, in the complex bone marrow microenvironment, the specific biological mechanism leading to this outcome requires further investigation.

Human mesenchymal stem cells (hMSCs) are nonhematopoietic stem cells that show multilineage differentiation potential and can selectively differentiate into adipocytes or osteoblasts under appropriate conditions [5]. In some pathological processes, such as ageing, osteoporosis, and diabetes, hMSCs tend to differentiate into adipocytes while weakening the differentiation ability of osteoblasts [6–8]. An increasing number of studies have shown that the lipogenic and osteogenic balance plays a key role in bone metabolism [9, 10]. Therefore, elucidation of the lipogenesis and differentiation of hMSCs and identification of potential therapeutic targets are urgently needed.

Matrix Gla protein (MGP) is a calcium-binding matrix protein that is synthesized mainly in bone, cartilage and other tissues and is closely related to osteogenic differentiation, atherosclerosis and vascular calcification [11–13]. MGP exerts its biological effects by regulating many cellular functions, including proliferation, differentiation, and signal transduction [14, 15]. Our previous studies showed that MGP inhibits osteoblast formation of MSCs by binding to BMP2 [16]. In adipocytes, the MGP protein was found to be a highly secreted novel adipokine [17]. Microarray sequencing confirmed that MGP expression was significantly increased during the lipid-forming differentiation of preadipocytes [18], and these studies indicated the importance of this molecule in lipid metabolism. In addition, Gla residues of the MGP protein bind calcium ions and carry out corresponding biological functions [19]. High intracellular Ca2+ concentrations ([Ca2+]i) were shown to enhance the accumulation of fat cells [20]. Furthermore, intracellular calcium signaling was shown to promote the later stages of this process and lead to lipid filling [21]. However, whether MGP can promote the lipogenic differentiation of MSCs and whether this effect is achieved by regulating intracellular Ca2+ are unclear.

FABP3 is a member of the intracellular lipid-binding protein superfamily and plays a key role in the intracellular transport of long-chain fatty acids [22]. Under hypoxic conditions, a decrease in the endogenous expression of FABP3 was found to impair the formation of lipid droplets [23]. Moreover, abnormal expression of FABP3 occurs in individuals with various diseases, such as obesity and diabetes [24, 25]. Previous studies have reported the effect of FABP3 on 3T3-L1 preadipocytes [26], and emerging studies indicate that the functionality of FABP3 can be modulated at the transcriptional level [27]; therefore, transcriptional regulation may have functional implications for the role of FABP3 in adipogenesis. Although these studies confirmed that FABP3 is important in the regulation of fat metabolism, the upstream effects of FABP3 require further analyses.

In this study, we explored the role of MGP in MSC adipogenesis in osteoporosis, both in vitro and in vivo. Further investigations revealed that MGP increases FABP3 gene transcription via the Ca2+/CaMKII/RIP140 axis, results in facilitating the adipogenic differentiation of MSCs. Notably, in vivo knockdown of MGP using rAAV9 effectively ameliorates excessive marrow adipogenesis and bone loss associated with osteoporosis. Collectively, these findings not only uncover a novel regulatory mechanism of MGP in MSC adipogenesis, but also offer valuable insights for the treatment of osteoporosis.

## Results

### MGP expression is increased in osteoporosis

**Fig. 1:**
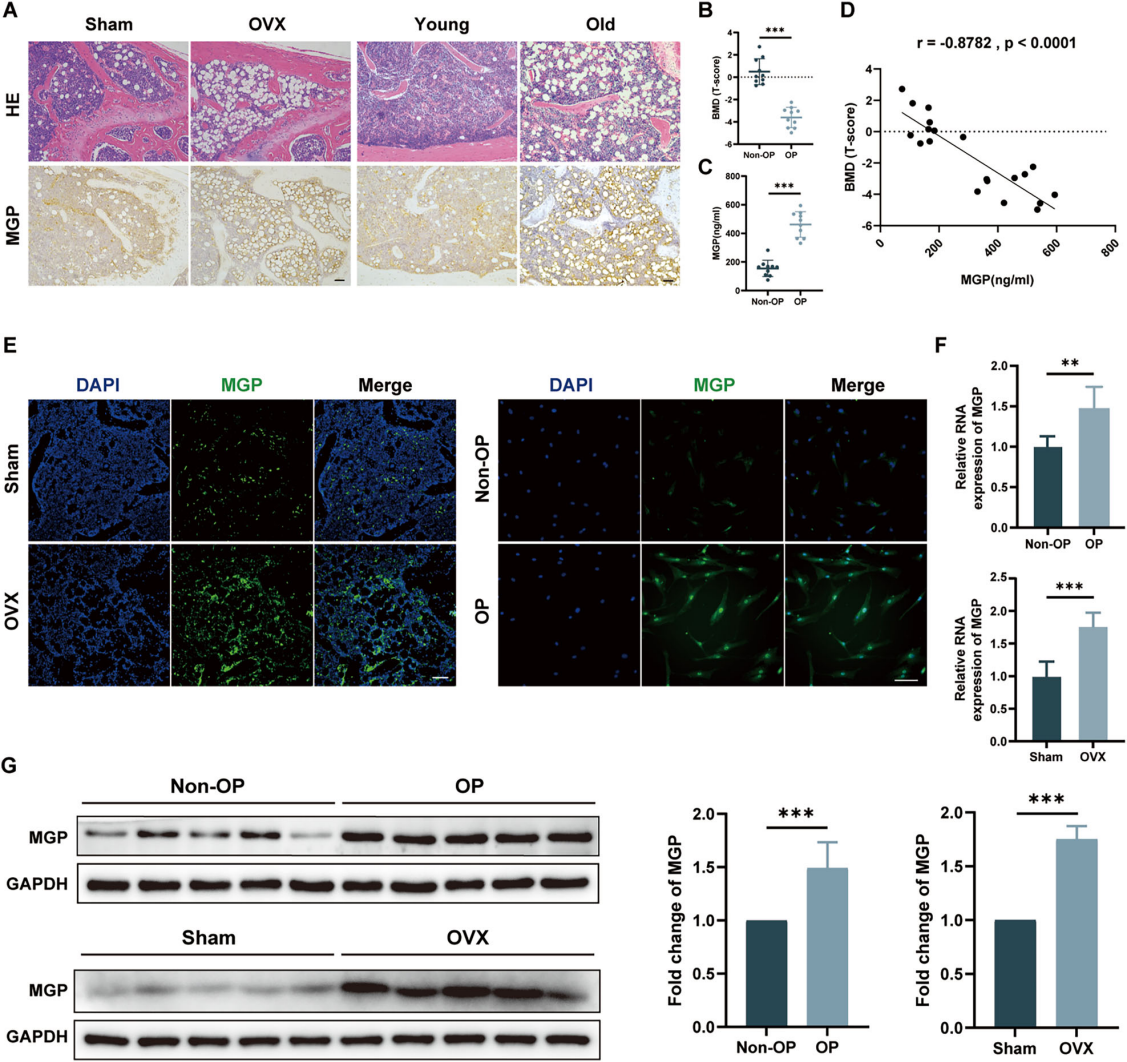
MGP expression is increased in osteoporosis. **A** H&E staining and IHC staining for MGP in the femurs of C57BL/6J mice subjected to sham surgery, OVX mice, young mice, and aged mice (*n* = 6). **B**, **C** The bone mineral density of nonosteoporotic (non-OP) patients and osteoporotic (OP) patients was measured via T scores, and the MGP expression levels in MSC lysates were analyzed via ELISA (*n* = 10). **D** The correlation between MGP expression and the T score is shown. **E** Immunofluorescence staining of MGP expression in bone marrow MSCs from non-OP and OP patients and the femurs of C57BL/6J mice subjected to sham surgery, OVX mice (*n* = 6). **F** The MGP expression levels in bone marrow MSCs from non-OP and OP patients and mice that underwent sham surgery and OVX were assessed through qRT‒PCR(*n* = 10). **G** The MGP protein levels in bone marrow MSCs from non-OP and OP patients and mice that underwent sham surgery and OVX were detected using Western blotting (*n* = 10). Scale bar = 50 μm. All the data are presented as the means ± SDs; Pearson correlation analysis was used to determine the correlation indices. Student’s *t* test or ANOVA was used to determine significant differences. ns not statistically significant; *P < 0.05; **P < 0.01; and ***P < 0.001.

The postmenopausal osteoporosis and senile osteoporosis mouse models were established as follows: The postmenopausal osteoporosis model was induced by conducting ovariectomy (OVX) on 8-week-old C57BL/6J mice, with an equal number of 8-week-old C57BL/6J mice undergoing sham surgery as control group. For the senile osteoporosis model, 18-month-old C57BL/6J mice were used, while 8-week-old C57BL/6J mice served as control group. As shown by H&E staining of mouse femur slices, a marked reduction in bone volume was observed in postmenopausal osteoporosis and senile osteoporosis groups. Additionally, IHC staining revealed that MGP expression was significantly upregulated in both the postmenopausal osteoporosis and senile osteoporosis groups compared to the control group (Fig. 1A). Furthermore, ten OP patients and ten Non-OP patients were recruited to explore the association between the severity of osteoporosis and MGP. T scores were calculated to assess bone density, and the OP patients exhibited significantly lower T score than the Non-OP patients did. Moreover, MSC lysates from the OP patients and Non-OP patients were extracted, and ELISA was used to determine the MGP protein concentration. We observed a significant increase in MGP expression in the OP patients, which was strongly negatively correlated with the T score (Fig. 1B–D). Besides, MGP expression level was significantly increased in tissues from the mice with postmenopausal osteoporosis and MSCs from OP patients, as shown by immunofluorescence staining (Fig. 1E). Furthermore, Western blotting and qRT‒PCR analyses also demonstrated a notable increase in MGP expression in the MSCs derived from the OP patients, consistent with the increase in MGP expression observed in MSCs from the OVX-induced mouse model (Fig. 1F, G). Taken together, these results indicate that MGP is critically involved in osteoporosis.

### MGP positively regulates the adipogenic differentiation of MSCs in vitro

**Fig. 2:**
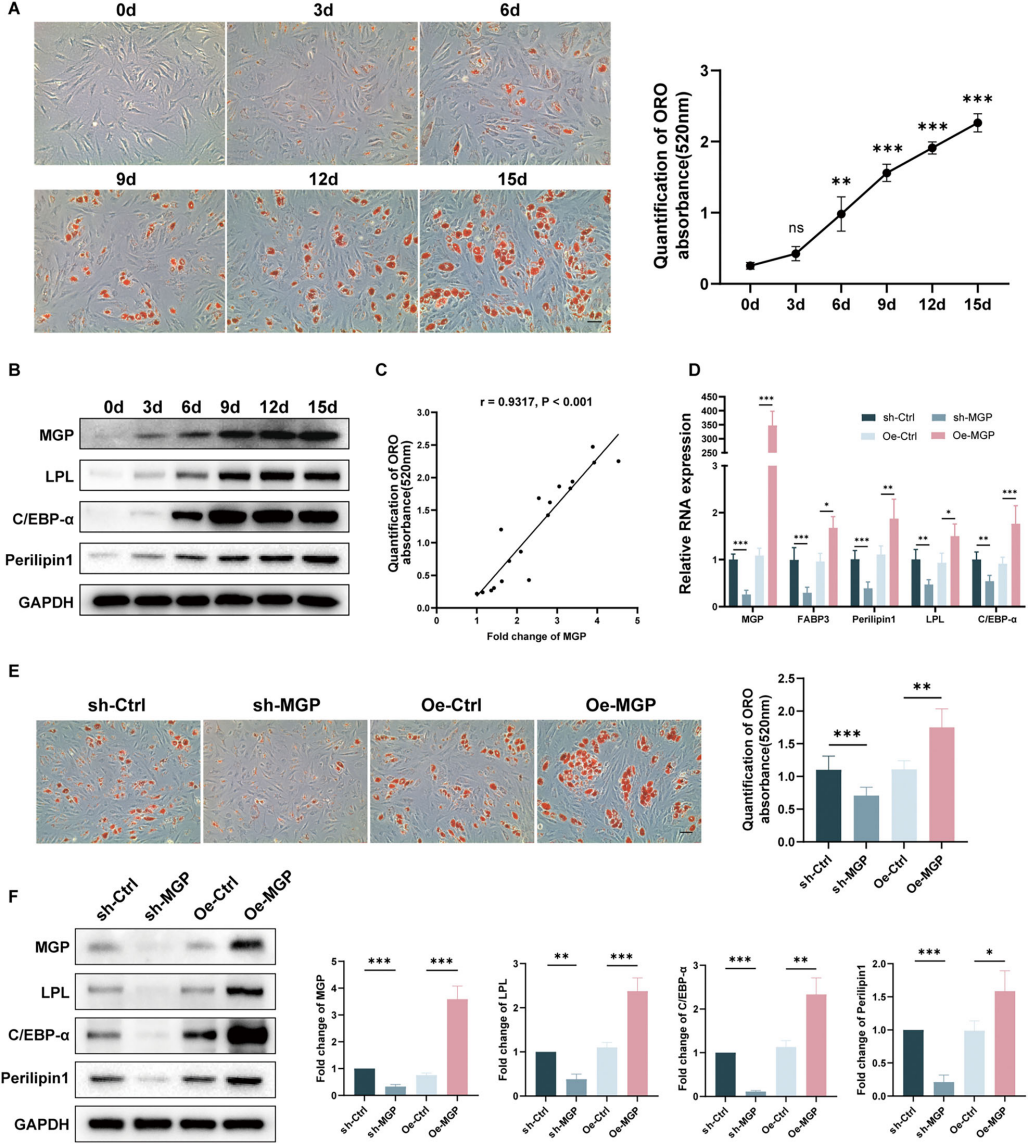
MGP positively regulates the adipogenic differentiation of MSCs in vitro. **A** ORO staining and quantification are shown along with the days of hMSC adipogenic differentiation (0–15 days). **B** The protein levels of MGP and the adipogenic markers C/EBP-α, LPL and Perilipin 1 were measured during hMSC adipogenic differentiation (0–15 days). **C** Quantification of the correlation between the expression levels of MGP and ORO staining during the adipogenic differentiation of MSCs. **D** The expression levels of MGP and the adipogenic markers C/EBP-α, LPL and Perilipin 1 in MSCs infected with sh-NC, sh-MGP, OE-NC, or OE-MGP lentiviruses were evaluated through qRT‒PCR. **E** ORO staining and quantitative analysis were performed to assess the level of adipogenic differentiation of MSCs infected with sh-NC, sh-MGP, OE-NC, or OE-MGP lentiviruses on the 10th day to assess adipogenic differentiation. **F** Protein levels of the adipogenic markers C/EBPα, LPL and perilipin 1 on the 5th day to assess adipogenic differentiation were assessed through Western blotting analysis, and the quantitative data are shown. Scale bar = 50 μm. All the data are presented as the means ± SDs; *n* = 6 per group. Student’s *t* test or ANOVA was used to determine significant differences. ns, not statistically significant; *P < 0.05; **P < 0.01; and ***P < 0.001.

During the development of osteoporosis, the pathological accumulation of bone marrow adipose tissue has been shown to occur, highlighting the essential role of the imbalance between osteogenesis and adipogenesis [28]. To clarify the impact of MGP on osteoporosis, we investigated the function of MGP in MSC adipogenic differentiation. ORO staining was used to evaluate the adipogenic differentiation capacity. ORO staining revealed a gradual increase in adipogenic differentiation from day 0 to 15 (Fig. 2A). Correspondingly, Western blotting indicated an increase in the protein levels of adipogenic markers (LPL, CEBP-α, and Perilipin 1) following adipogenic induction, coupled with a gradual increase in MGP expression during this process (Fig. 2B). Furthermore, we conducted correlation analysis between MGP expression and ORO staining and revealed a strong positive correlation throughout the adipogenic differentiation of MSCs (Fig. 2C). To investigate the specific regulatory impact of MGP on MSC adipogenic differentiation, we designed lentiviruses carrying MGP shRNA and overexpressing MGP. The adipogenic capacity of MSCs in various groups was assessed following a 10-day period of adipogenic induction. ORO staining showed that the MSCs treated with MGP shRNA exhibited lower adipogenic differentiation than the control shRNA-treated MSCs, suggesting that MGP knockout decreases the adipogenic potential. Conversely, overexpression of MGP significantly elevated the formation of adipocytes (Fig. 2E). Adipogenesis-associated markers were also assessed. MGP overexpression in MSCs led to elevated protein levels of LPL, CEBPα, and Perilipin 1, as determined by qRT-PCR (Fig. 2D) and Western blotting (Fig. 2F), whereas MGP knockdown resulted in a reduction in the levels of these proteins during adipogenic induction. In conclusion, these results suggest that MGP plays a positive regulatory role in MSC adipogenic differentiation in vitro.

### FABP3 is a key downstream target of MGP-mediated adipogenic differentiation of MSCs

**Fig. 3:**
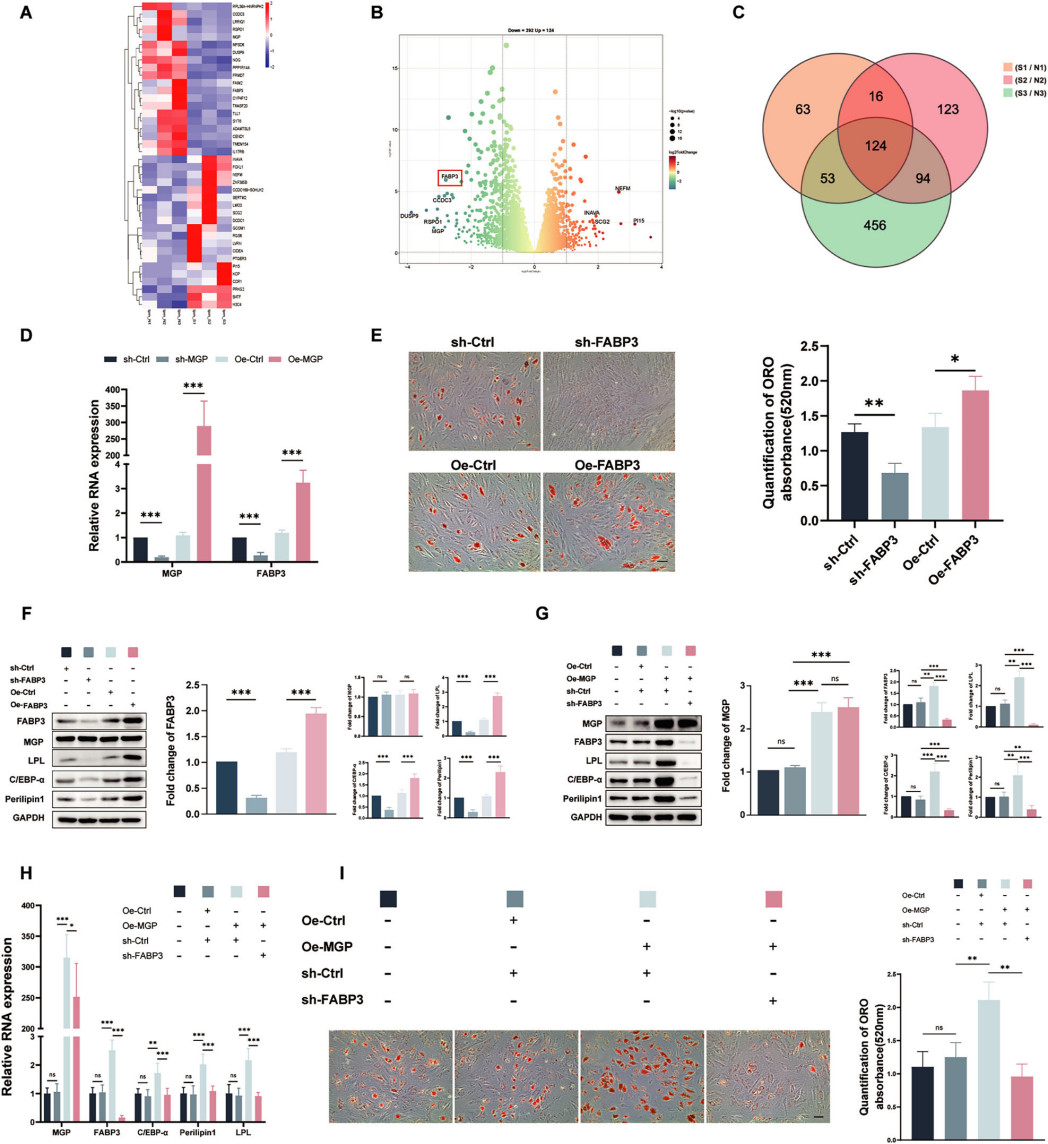
FABP3 is a key downstream target of MGP-mediated adipogenic differentiation of MSCs. **A** Cluster heatmap, **B** Volcano map and **C** Venn diagram for MSCs infected with sh-NC or sh-MGP lentivirus on the 3rd day of adipogenic differentiation are shown. **D** After MSCs were infected with sh-NC, shMGP, OE-NC, or OE-MGP lentiviruses, the relative RNA expression of FABP3 was determined via qRT–PCR. **E** ORO staining and quantification of MSCs infected with sh-NC, sh-FABP3, OE-NC or OE-FABP3 lentivirus on the 10th day to assess adipogenic differentiation. **F** Protein levels of FABP3, MGP and the adipogenic markers C/EBP-α, LPL and Perilipin 1 in MSCs infected with sh-NC, sh-FABP3, OE-NC or OE-FABP3 lentiviruses on the 5th day were detected. **G**, **H** The protein and relative RNA expression levels of MGP, FABP3, and the adipogenic markers C/EBP-α, LPL and Perilipin 1 after FABP3 knockdown or/and MGP overexpression using lentiviruses were determined via Western blotting analysis and qRT–PCR. **I** ORO staining and quantification were performed after 10 days to assess adipogenic differentiation in cells with FABP3 knockdown and/or MGP overexpression. Scale bar = 50 μm. All the data are presented as the means ± SDs; *n* = 6 per group. Student’s *t* test or ANOVA was used to determine significant differences. ns not statistically significant; *P < 0.05; **P < 0.01; and ***P < 0.001.

To further elucidate the downstream mechanisms by which MGP regulates adipogenic differentiation, we examined the expression of distinct genes in MSCs from the control and MGP knockdown groups via RNA sequencing after 3 days of adipogenic induction. A heatmap visually represents genes with differential expression before and after knockdown (Fig. 3A). In the MGP knockdown group, 392 genes were downregulated and 124 genes were upregulated compared to those in the control group (Fig. 3B). Venn diagram analysis identified 124 significantly differentially expressed mRNAs (Fig. 3C).

The results of the GO analyses indicated that the differentially expressed genes were enriched in biological processes including “intracellular calcium ion homeostasis”, “mesenchymal cell differentiation”, and “fatty acid metabolic processes” (Supplementary Fig. S1). KEGG analyses indicated the potential impacts of MGP knockout on “lipid metabolism”, “signal transduction”, and “transport and catabolism” in Cellular Processes, Environmental Information Processing, and Genetic Information Processing categories (Supplementary Fig. S2).

We found a decrease in the expression of FABP3, a fatty acid transport protein, in the RNA-seq data. Given the reported importance of FABP3 in adipogenic differentiation, we performed qRT‒PCR to assess the regulatory impact of MGP on FABP3 expression. After MGP knockdown, FABP3 expression decreased, consistent with the RNA-seq data, whereas overexpression of MGP resulted in a significant upregulation of FABP3 expression (Fig. 3D). Taken together, these results indicate that MGP positively modulates the expression of FABP3 in MSCs. To further validate the impact of FABP3 on MSC adipogenic differentiation, we knocked down and overexpressed FABP3 using lentiviruses. As shown by ORO staining, downregulation of FABP3 impaired the adipogenesis of MSCs, whereas the overexpression of FABP3 promoted adipogenesis (Fig. 3E). In addition, FABP3 downregulation in MSCs led to a decrease in adipogenic differentiation markers was detected as determined by Western blotting, while overexpressing FABP3 increased the expression, however, no significant change in MGP expression levels was observed following either FABP3 overexpression or knockdown (Fig. 3F). These findings indicate that the adipogenic differentiation capacity of MSCs is decreased following FABP3 depletion. To further verify that MGP promotes MSC adipogenic differentiation through the regulation of FABP3, we co-overexpressed MGP and downregulated FABP3 in MSCs. The results of Western blotting and qRT-PCR revealed that MGP overexpression significantly increased the protein and mRNA levels of adipogenic markers, but these effects were substantially attenuated by downregulating FABP3 (Fig. 3G, H). Consistent with these findings, ORO staining indicated that MGP significantly enhanced MSC adipogenic differentiation, and the downregulation of FABP3 counteracted the stimulatory effect mediated by MGP (Fig. 3I). Overall, MGP positively regulates the expression of FABP3 and promotes MSC adipogenic differentiation.

### MGP activates the calcium signaling pathway in MSCs

**Fig. 4:**
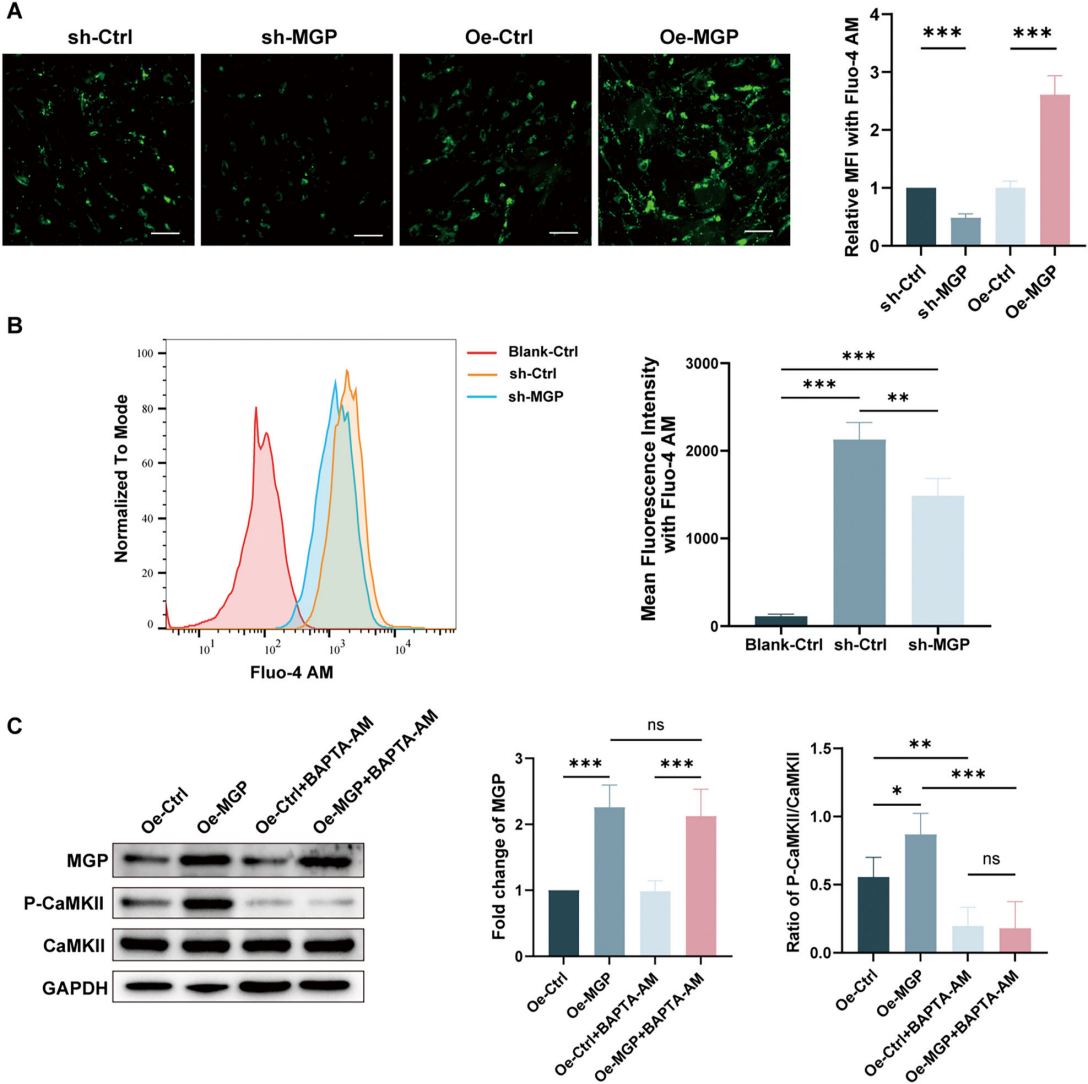
MGP activates the calcium signaling pathway in MSCs. **A** The level of intracellular calcium ions in MSCs infected with sh-NC, sh-MGP, OE-NC, or OE-MGP lentiviruses was assessed through calcium fluorescence, and the quantitative data are shown. **B** The level of intracellular calcium ions in MSCs infected with sh-NC or shMGP was determined through flow cytometry using Fluo-4 AM. **C** Protein levels and quantification of MGP, CaMKII, P-CaMKII, and GAPDH expression after infection with OE-NC or OE-MGP lentiviruses and/or addition of the calcium ion chelator BAPTA-AM. Scale bar = 50 μm. All the data are presented as the means ± SDs; *n* = 6 per group. Student’s *t* test or ANOVA was used to determine significant differences. ns not statistically significant; *P < 0.05; **P < 0.01; and ***P < 0.001.

As described above, the results from RNA-seq bioinformatics analysis suggest a close association between MGP and intracellular calcium ion homeostasis. We hypothesized that the process by which MGP promotes MSC adipogenesis may involve dynamic changes in the intracellular Ca2+ balance. To further validate the regulatory association between MGP and intracellular calcium ions, we used the fluorescent probe Fluo-4 AM. The fluorescence microscopy and quantitative analysis revealed a marked reduction in Ca2+ influx in the MSCs transfected with sh-MGP compared to that in the control group, while overexpressing MGP resulted in a significant increase in Ca2+ influx (Fig. 4A). Flow cytometry was also employed for the quantitative assessment of intracellular calcium levels in MSCs. The mean fluorescence intensity of the sh-MGP MSCs was significantly lower than that of the sh-control group (Fig. 4B). Given that CaMKII serves as a mediator in various signaling pathways induced by Ca2+ influx, we speculated that CaMKII might mediate MGP-promoted adipogenic differentiation. After MGP was overexpressed in MSCs, the P-CaMKII/CaMKII ratio significantly increased, indicating the activation of CaMKII during this process (Fig. 4C). To examine the impact of intracellular Ca2+ chelation on MGP-promoted CaMKII phosphorylation, we used the calcium chelator BATPA-AM. The results showed that BATPA-AM could reverse the MGP-induced increase in CAMKII phosphorylation (Fig. 4C). The results above demonstrated that MGP might activate CaMKII phosphorylation by promoting Ca2+ influx.

### RIP140 is involved in MGP-induced adipogenic differentiation through binding to the FABP3 promoter

**Fig. 5:**
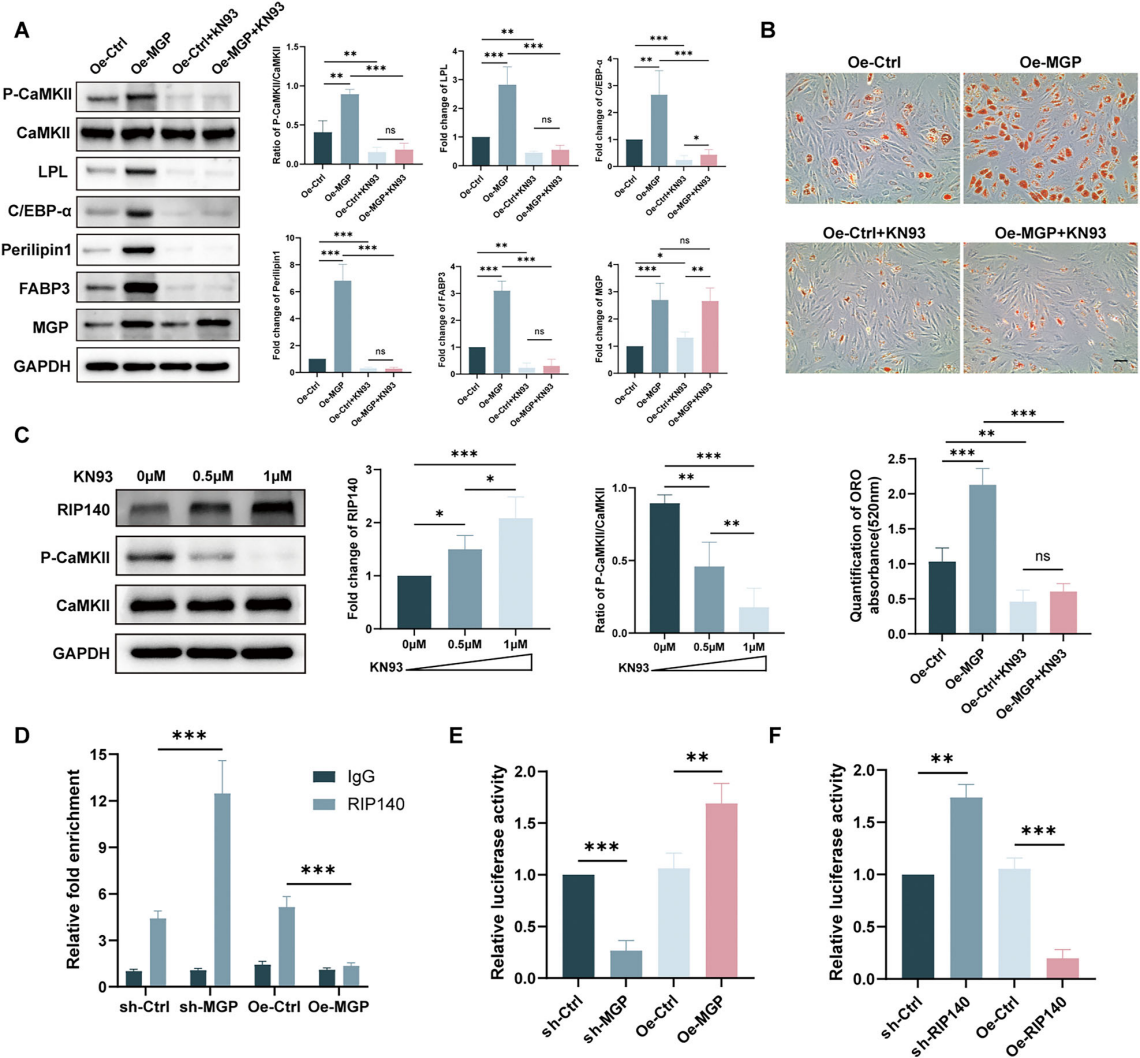
RIP140 is involved in MGP-induced adipogenic differentiation through binding to the FABP3 promoter. **A** Protein levels and quantification of the levels of FABP3, P-CaMKII, GAPDH and the adipogenic markers C/EBP-α and Perilipin 1 after overexpressing MGP and/or adding KN93, a P-CaMKII inhibitor. **B** ORO staining and quantitative analysis after infection with OE-NC or OE-MGP lentiviruses and/or the addition of KN93, a P-CaMKII inhibitor. **C** The protein levels and quantification of GAPDH, P-CaMKII, CaMKII and RIP140 levels are shown along with the concentration gradient of KN93. **D** CUT&Tag-qPCR was performed with an anti-RIP140 antibody or normal mouse IgG in MSCs infected with OE-NC or OE-MGP lentiviruses. **E**, **F** The interaction between MGP or RIP140 and FABP3 was detected through a dual-luciferase reporter system. Scale bar = 50 μm. All the data are presented as the means ± SDs; *n* = 6 per group. Student’s *t* test or ANOVA was used to determine significant differences. ns, not statistically significant; *P < 0.05; **P < 0.01; and ***P < 0.001.

To further validate the role of CaMKII in MGP-induced adipogenic differentiation, we employed KN-93, a competitive inhibitor of P-CaMKII. Western blotting demonstrated a reversal of the MGP-induced upregulation of FABP3 expression when CaMKII phosphorylation was inhibited by KN-93 (Fig. 5A). Notably, the expression of adipogenic markers was significantly downregulated during this process. ORO staining revealed that KN-93 effectively prevented the adipogenic accumulation caused by MGP overexpression (Fig. 5B). In summary, MGP promotes MSC adipogenic differentiation through the Ca2+/CaMKII pathway, emphasizing the pivotal role of CaMKII in mediating this effect.

RIP140 is a crucial signaling factor in energy metabolism that negatively regulates FABP3 in skeletal muscle cells [29]. CaMKII phosphorylation leads to the degradation of the RIP140 protein [30]. Therefore, we investigated whether the MGP-induced upregulation of FABP3 expression, which results in MSC adipogenic differentiation, involves the regulation of RIP140. Western blotting revealed a dose-dependent increase in RIP140 expression in MSCs when CaMKII phosphorylation was inhibited by KN-93 (0 µM, 0.5 µM, and 1 µM) (Fig. 5C). To determine if the change in protein levels stemmed from changes in mRNA levels, qRT-PCR assays were conducted. The results illustrated that the levels of RIP140 mRNA remained unchanged (Supplementary Fig. S3), suggesting that CaMKII governs RIP140 expression primarily at the protein level. To examine the impact of MGP on RIP140 at the FABP3 promoter in MSCs, we conducted CUT&Tag-qPCR analysis and determined that, compared with the sh-control treatment, MGP knockdown elevated the binding of RIP140 to the FABP3 promoter, whereas MGP overexpression substantially reduced the enrichment of RIP140 at the FABP3 promoter (Fig. 5D). To assess the effect of MGP or RIP140 on FABP3 transcription, we utilized a firefly luciferase reporter to construct the pGL4.10-FABP3 plasmid carrying the FABP3 sequence upstream of the firefly luciferase reporter gene. The reporter plasmid was co-transfected into HEK293T cells with lentiviruses with knockdown/overexpression of MGP and a control vector to explore the potential regulation of the FABP3 promoter by MGP. MGP knockdown resulted in decreased luciferase activity, whereas MGP overexpression significantly increased the luciferase activity compared to that in the control group in HEK293T cells (Fig. 5E). However, when we co-transfected lentiviruses overexpressing or knocking down RIP140 and the reporter plasmid into HEK293T cells, luciferase assays showed that RIP140 gene knockdown increased FABP3 promoter transcriptional activity while overexpressing RIP140 decreased FABP3 promoter transcriptional activity in HEK293T cells (Fig. 5F). Taken together, these findings indicate that MGP-induced CaMKII phosphorylation promotes RIP140 degradation, diminishing its inhibitory effect on FABP3 transcription and ultimately facilitating adipogenesis in MSCs.

### Treatment with rAAV9-Mgp alleviates osteoporosis and excessive bone marrow adipose tissue in mice

**Fig. 6:**
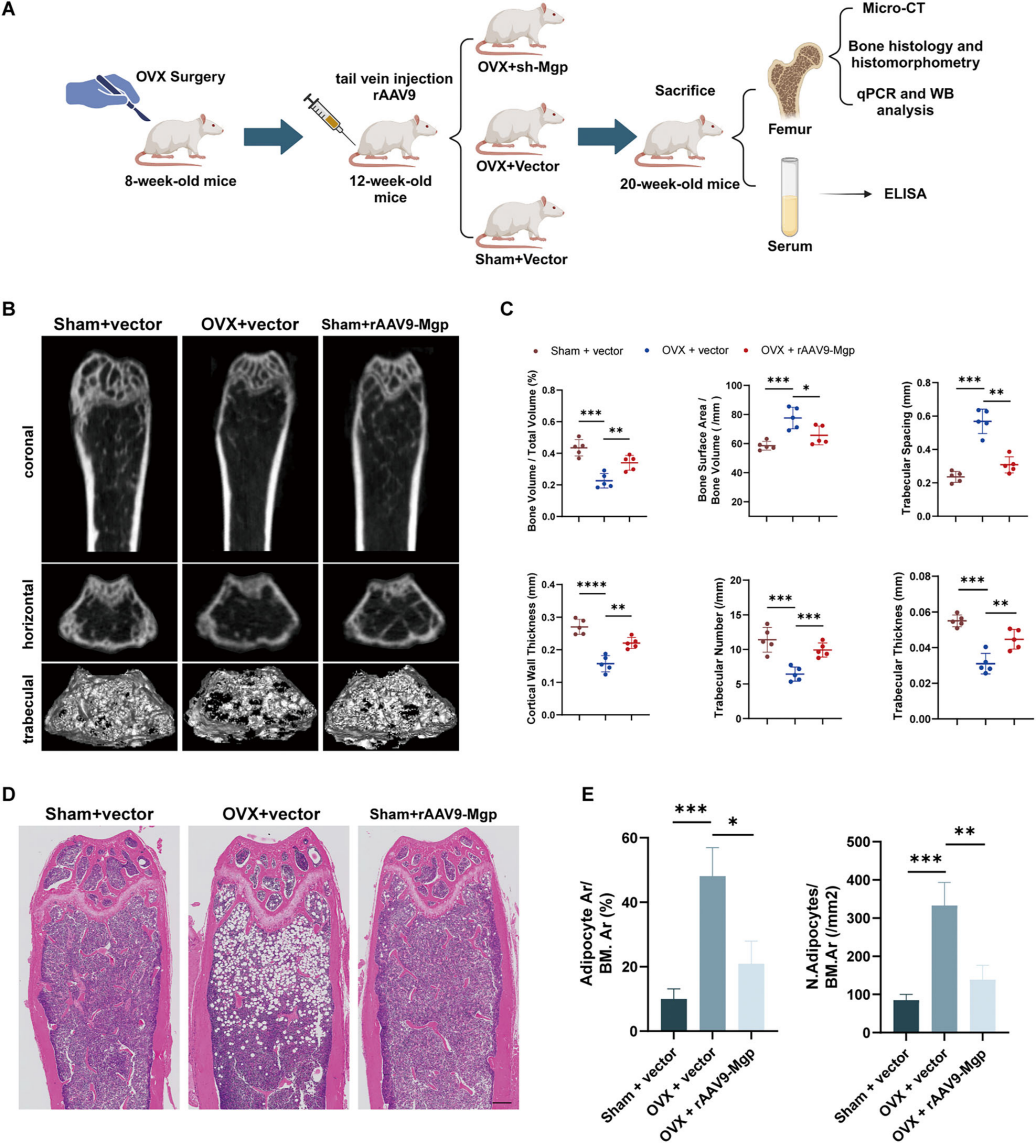
Treatment with rAAV9-Mgp alleviates osteoporosis and excessive adipogenesis in mice. **A** Schematic of the timeline and workflow for the in vivo studies in mice to confirm the function of MGP. **B** Micro-CT scans were performed on coronal and horizontal sections of femurs from mice in the control group, mice with postmenopausal osteoporosis, and rAAV-9-Mgp-treated mice. Cortical bone and trabecular bone structure were analyzed through three-dimensional micro-CT reconstruction. **C** BV/TV, BS/BV, Tb.Sp, Ct.Th, Tb.N and Tb.Th were assessed in the control mice, mice with postmenopausal osteoporosis or rAAV-9 Mgp-treated mice. **D** H&E staining was performed on tissues from the control mice, mice with postmenopausal osteoporosis or rAAV-Mgp-treated mice. **E** The lipid level of each group was assessed by calculating the number and area of adipocytes. Scale bar = 250 μm. All the data are presented as the means ± SDs; *n* = 6 per group. Student’s *t* test or ANOVA was used to determine significant differences. ns not statistically significant; *P < 0.05; **P < 0.01; ***P < 0.001; and ****P < 0.0001.

After validating MGP and its regulatory mechanisms in adipogenic differentiation in vitro, we aimed to assess its potential as a therapeutic target for osteoporosis in vivo. To achieve this goal, we established an OVX-induced osteoporosis model and employed rAAV9 for in vivo MGP knockdown. OVX was performed on eight-week-old C57BL/6 mice, followed by tail vein injection of rAAV9 at 12 weeks. Subsequent experiments were conducted on the femurs extracted at 20 weeks, as illustrated in the schematic (Fig. 6A). Micro-CT scans of the mouse femurs revealed a significant decrease in trabecular bone quantity in the ovariectomized mice. Notably, rAAV9MGP treatment mitigated bone density loss in the osteoporotic mice, normalizing trabecular morphometric parameters (BV/TV, BS/BV, Tb.Th, Tb.N, Tb.Sp, and Ct.Th) compared to those of the ovariectomized mice injected with the rAAV9 vector, as depicted in the 3D microstructure reconstruction (Fig. 6B, C).

To confirm the regulatory role of MGP in bone marrow adipogenesis in ovariectomized mice, we conducted H&E staining of mouse femurs to observe adipocytes within the bone marrow. Consistent with the micro-CT findings, the ovariectomized mice exhibited a significant reduction in trabecular bone quantity and an increase in fat cell number and area, whereas rAAV9-Mgp treatment significantly reversed the OVX-induced bone loss and adipocyte accumulation (Fig. 6D, E). In addition, The decrease in serum concentrations of bone formation markers (OCN, PINP) induced by OVX was also completely reversed (Supplementary Fig. S4). The dual functionality of MGP in promoting bone marrow adiposity, coupled with our previous findings that MGP inhibits MSC osteogenic differentiation, suggests a regulatory role for MGP in the delicate balance between adipogenic and osteogenic differentiation.

## Discussion

**Fig. 7:**
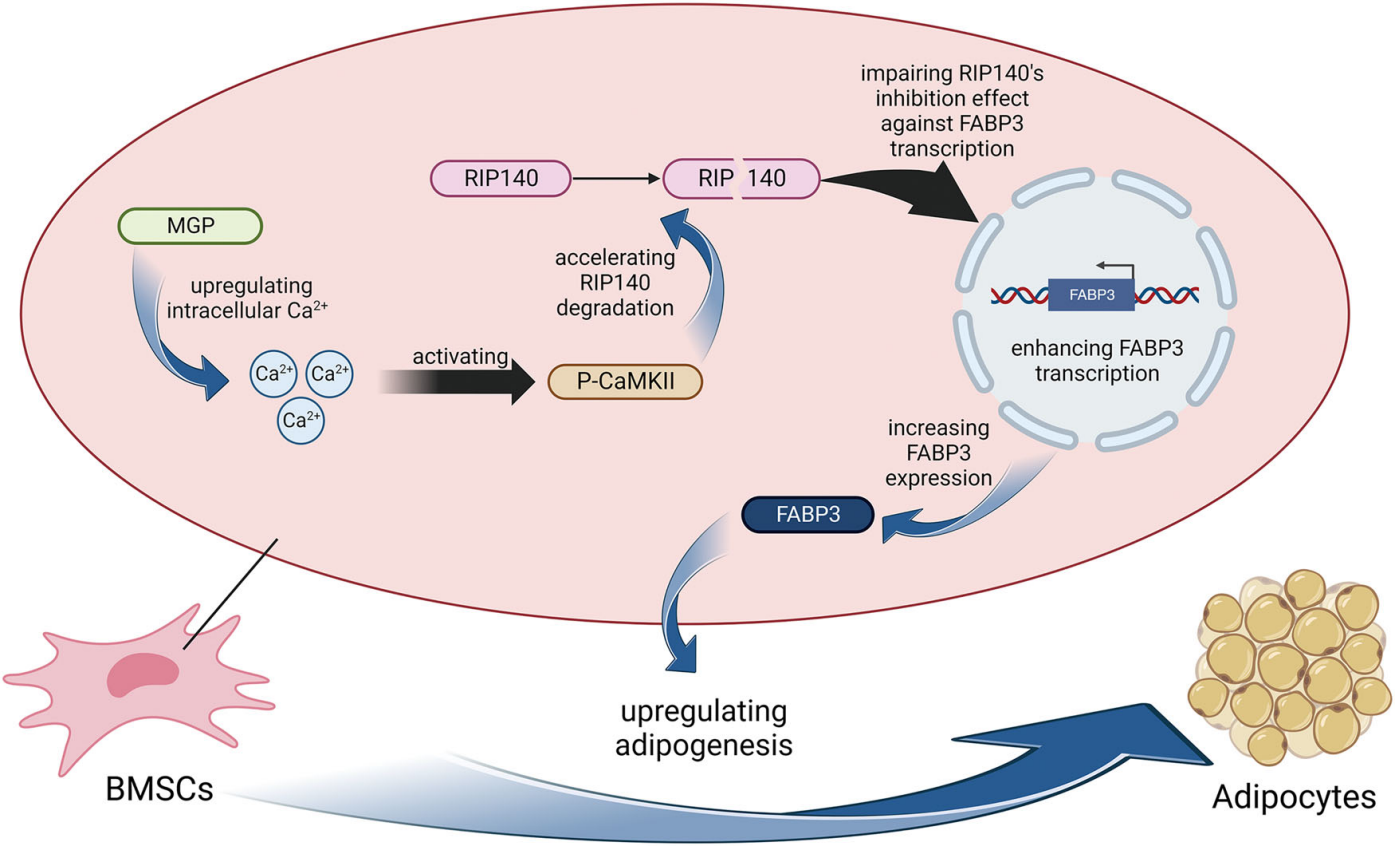
Schematic diagram of how MGP positively regulates the adipogenic differentiation of MSCs via the Ca2+/CaMKII/RIP140/FABP3 axis. Created with BioRender.com.

In our previous research, we confirmed that APPL1 suppresses the expression of MGP in MSCs and activates the BMP2 pathway, increasing the osteogenic differentiation of MSCs [16]. In this study, we detected elevated MGP expression in bone marrow MSCs from patients with osteoporosis and osteoporotic mouse models. Moreover, we found a robust correlation between MGP expression and the severity of osteoporosis, revealing the critical involvement of MGP in osteoporotic progression. This pathological process is due to the upregulation of FABP3 transcription by MGP, which consequently promotes adipogenic differentiation in MSCs and abnormal accumulation of bone marrow adipose tissue. Mechanistically, MGP induces RIP140 protein degradation by activating the Ca2+/CaMKII pathway, consequently attenuating its capacity to repress FABP3 transcription and thereby strongly increasing FABP3 expression (Fig. 7). Importantly we demonstrated the therapeutic potential of sh-MGP in vivo, suggesting its potential in osteoporosis treatment.

Bone marrow-derived MSCs (BMSCs) are non-hematopoietic cells within the bone marrow that can undergo multidirectional differentiation, including adipogenesis, osteogenesis and chondrogenesis [31]. Our previous reports have emphasized the potential therapeutic applications of BMSCs in various conditions, such as osteoporosis, ankylosing spondylitis, and osteoarthritis [32–34]. Generally, the processes of adipogenic and osteogenic differentiation in MSCs are mutually regulated. An imbalance between osteogenic and adipogenic differentiation is a pivotal pathogenic factor in osteoporosis. Therefore, intensive exploration of the crucial targets and signaling pathways orchestrating MSC differentiation into adipocytes could contribute to the effective utilization of MSCs in treating conditions such as osteoporosis. Currently, substantial discussion in osteoporosis-related research has centerd on osteoblasts. However, further investigation into the augmented adipogenic anomalies in osteoporosis is essential. An increase in MGP expression was observed during adipogenic differentiation in mouse 3T3-L1 cells through single-cell sequencing [35]. However, upon stimulation with y(+)-cholesten-3-one (CN), osteogenic differentiation was significantly increased, and this change was accompanied by the upregulation of MGP expression [36]. Nevertheless, this research was limited to murine-derived MSCs and did not elucidate the broader influence of these MSCs on overall bone mass alterations in humans. Due to conflicting findings concerning the effect of MGP on MSCs, comprehensive research is needed to elucidate the biological importance and potential mechanisms of MGP in osteoporosis. Our study demonstrated that rAAV9mediated knockout of MGP in mice prevents bone loss in an osteoporotic mouse model and diminishes the overall volume of marrow adipose tissue. However, the exact mechanism through which MGP positively affects MSC adipogenic differentiation in osteoporotic pathogenesis requires further exploration.

Despite the crucial involvement of MGP in MSC adipogenic differentiation, knowledge of its downstream mechanisms in this process is lacking. Consequently, by employing transcriptomic sequencing and qRT‒PCR, we discovered that MGP knockout notably suppressed FABP3 expression, while MGP overexpression in MSCs produced contrasting outcomes. Fatty acid-binding proteins (FABPs) play pivotal regulatory roles in lipid generation, metabolism, and signal transduction pathways [37]. Previous evidence indicates that FABP3 may regulate muscle homeostasis by inducing endoplasmic reticulum stress through membrane lipid component mediation [38]. The protein expression levels of FABP3 in hTERT-immortalized MSCs exhibited a negative linear correlation with femoral neck and lumbar spine bone density [39]. Liu et al. reported that aberrant FABP3 accumulation in aged mice can induce BMSC ageing, consequently leading to diminished osteogenesis and heightened adipogenesis [40]. Nonetheless, the osteoporosis model employed in that study was aged mice, and further exploration of the role of FABP3 in postmenopausal osteoporosis, specifically concerning primary osteoporosis after menopause, requires comprehensive validation in human MSCs and OVX-induced mouse models to determine the potential upstream mechanisms involved in postmenopausal osteoporosis. Additionally, in this study, we verified the regulatory role of FABP3 in the adipogenic differentiation of MSCs. FABP3 knockdown/overexpression has inhibitory/promotional effects on adipogenic differentiation. Our rescue experiments revealed that deleting FABP3 can counteract the increase in adipogenesis induced by MGP overexpression. Consequently, we confirmed the positive regulatory function of FABP3 in MSC adipogenic differentiation, confirming that this biological process stems from its downstream effect induced by MGP.

As a calcium-binding protein, MGP plays an inhibitory role in the pathogenesis of vascular and cartilage calcification. Previous extensive research has focused primarily on the extracellular biological functions of MGP, overlooking its important role within the cytoplasm. When Ca2+ is present, BMP-2 binds to the Gla-rich regions of MGP, which mediate the functions of MGP as an inhibitor of BMP-2. Given our previous research findings, we hypothesize that Ca2+ might be a crucial signaling mediator that facilitates the MGP-mediated promotion of adipogenic differentiation. Previous investigations underscore the critical regulatory effect of intracellular Ca2+-dependent mitochondrial metabolism on maintaining lipid homeostasis in adipocytes [41]. Intracellular Ca2+ has been identified as a trigger for the activation of CaMKII [42]. Furthermore, the Ca2+/CaMKII/JNK pathway plays a crucial role in adipocyte insulin resistance [43]. Our findings indicate that MGP enhances intracellular calcium flux, consistent with previous results [44]. However, CaMKII promotes adipogenic differentiation in a time-dependent manner [45], revealing the bidirectional effects of the Ca2+/CaMKII pathway in the current study. In this study, for the first time, we unveiled the biological mechanism underlying the promotion of MSC adipogenic differentiation via the Ca2+/CaMKII pathway and confirmed the involvement of MGP in increasing FABP3 expression through this pathway.

RIP140 acts as a corepressor of nuclear receptors and plays a pivotal role in human metabolic processes [46]. RIP140 knockdown in mice was reported to induce amplified bone resorption and diminished bone formation [47]. Employing the inhibitor KN-93, we confirmed a negative correlation between CaMKII and RIP140, which was consistent with the findings of a previous study [30]. When the concentration of KN-93 was increased, the protein expression of RIP140 gradually increased as CaMKII was inhibited in MSCs. Research has demonstrated an interaction between RIP140 and FABP3 [29]. Consequently, this discovery prompted us to speculate that MGP likely modulates FABP3 via the Ca2+/CaMKII/RIP140 pathway to foster adipogenic differentiation in MSCs. Using CUT&Tag and qPCR assays and dual-luciferase reporter experiments, we confirmed that RIP140 in MSCs binds to the DNA sequence promoter of FABP3, leading to the inhibition of its transcription. RIP140 expression exhibited an inverse correlation with FABP3 expression. In our study, we verified that MGP governs adipogenic differentiation by upregulating the transcription of FABP3 in MSCs and contributes to osteoporosis by affecting the Ca2+/CaMKII/RIP140 pathway.

To date, primary treatments for osteoporosis involve medications such as teriparatide and denosumab, which are designed to promote bone formation or impede osteoclast function. However, using these drugs alone may result in specific side effects. For example, teriparatide can induce muscle pain symptoms in patients, and its duration of use is limited. Another limitation is that the majority of existing medications do not directly address the balance between the osteogenic and adipogenic differentiation of BMSCs [48]. Through an extensive examination of the direct regulatory mechanisms governing osteogenic and adipogenic balance, we concluded that developing drugs targeting bone marrow fat and combining conventional antiosteoporotic drugs with those targeting bone marrow fat may further optimize the treatment effects. This study has certain limitations. For instance, nanocarriers were not used for bone-targeted drug delivery to enhance targeting efficiency. In addition, genetic and environmental factors affecting bone health, particularly their influence on MGP’s role in bone metabolism, require more attention, especially in populations with high MGP expression. To address this, we will conduct a longitudinal analysis with an expanded sample size across diverse populations in our forthcoming research.

## Materials and methods

### Isolation and culture of MSCs

The study received approval from The Ethics Committee of The Eighth Affiliated Hospital, Sun Yat-sen University. Subsequently, ten patients diagnosed with osteoporosis (OP) and ten non-osteoporotic control subjects (Non-OP) were recruited and received full disclosure of the study’s aims and methodology prior to providing informed consent. Thereafter, all participants voluntarily gave their informed consent. Further details regarding the enrolled participants can be found in Supplementary Table S1. Bone marrow was obtained from humans as previously described [49]. After MSCs reached 80 to 90% confluence, detachment was facilitated with 0.25% trypsin, and the digestion process was halted by the addition of fetal bovine serum The cells were subsequently seeded in flasks with Dulbecco’s modified Eagle’s medium(DMEM, Gibco) containing 10% fetal bovine serum. MSCs were cultured and expanded, and cells from passage 3-5 were utilized for the subsequent experiments.

Murine bone marrow MSCs from mice with osteoporosis (mice that underwent OVX) and control (mice that underwent sham surgery) C57BL/6J mice were obtained after clearing the musculature and tendons enveloping the femurs. Bone marrow was aspirated from the medullary cavity using an injector and introduced into complete αMEM (Gibco) supplemented with 10% fetal bovine serum and 100 IU/ml penicillin– streptomycin. After suspension, the cells were cultured in 25 cm^2^ flasks using medium, and the medium was changed every three days. Subsequently, MSCs were seeded into a 12-well plate for subsequent experiments.

### Establishment of the osteoporosis model in mice

In this study, eight-week-old C57BL/6J mice were purchased from Guangdong Medical Laboratory Animal Center. The mice underwent sham (sham) or ovariectomy (OVX) surgery after being anaesthetized with isoflurane. The mice were given a 2-month recovery period following the ovariectomy surgery. Besides, At 18 months of age, the mice were utilized as models for senile osteoporosis in a related study, with 8-week-old mice serving as the control group. The animals were maintained according to the guidelines for the Care of Laboratory Animals. Ovariectomized mice and aged mice were established to determine whether MGP was involved in the pathogenesis of osteoporosis. The mice were subsequently euthanized and utilized for various assays.

### RNA sequencing (RNA-seq) data analysis

The transcriptome sequencing data, which included transcripts per million (TPM) or transcripts per kilobase of exon model per million mapped reads (FPKM) and gene counts, was generated by BGI Gene (Shenzhen China). Subsequent bioinformatics analysis was carried out using R (v4.3.2). Differentially expressed gene analysis was performed using DESeq2 (v1.42.0). A volcano map was generated using ggplot2 (v3.4.4), and a heatmap was created using pheatmap (v1.0.12). Gene Ontology (GO) enrichment was conducted using clusterProfiler (v4.10.0) and visualized with ggplot2 (v3.4.4). Kyoto Encyclopedia of Genes and Genomes (KEGG) pathway enrichment analyses was conducted using BGI’s Dr. Tom Multi-Omics Data Visualization System.

### rAAV-9 MGP construction

Recombinant adeno-associated virus type 9 (rAAV9) vectors expressing short hairpin RNAs (shRNAs) targeting MGP (rAAV9-Mgp) or a control hairpin (rAAV9-vector) were purchased from OBiO Technology (Shanghai China). Twelve-week-old wild-type mice (C57BL/6J) were intravenously injected with adeno-associated virus (3 × 10^11 ^v.g./mouse) for 8 weeks.

### Enzyme‐linked immunosorbent assay (ELISA)

In this study, an ELISA was used to determine the content of MGP in MSC lysates according to the manufacturer’s instructions for the ELISA Kit (ELK Biotechnology). The MGP concentrations in the samples were determined based on a standard curve.

### Adipogenic differentiation induction

MSCs were cultured in 12-well plates at a density of 6 × 10^4^ cells per well. Adipogenic differentiation was induced by with the addition of adipogenic components, including DMEM (10% FBS), 0.5 mM 3-isobutyl-1-methylxanthine, 0.2 mM indomethacin, 10 mg/ml insulin, and 1 μM dexamethasone, to the culture medium. The culture medium was refreshed every 3 days, and upon completion of the culture period (3–15 days), the cells were stained with oil red O (ORO) for visualization of adipocyte differentiation.

### ORO staining and quantification

After a specific induction period of adipogenic differentiation, MSCs were fixed with 4% paraformaldehyde for 20 min. Subsequently, the cells were stained with ORO working solution for 20 min at room temperature. After staining, the cells were washed three times with PBS and observed under a microscope. The stained cells were destained with 600 μl of isopropyl alcohol for extraction. A 200 μl aliquot was transferred to a 96-well plate, and the absorbance was measured at 520 nm.

### Western blotting

The cells were lysed using RIPA buffer with protease and phosphatase inhibitors for 30 min on ice. After lysis, the obtained lysates were centrifuged at 14,000 rpm for 30 min at 4 °C. Subsequently, the protein concentrations in the lysates were determined using the BCA Protein Assay Kit, and equivalent quantities of proteins were combined with 5× sodium dodecyl sulfate (SDS) loading buffer, followed by boiling for 10 min. After boiling, the proteins were separated via SDS‒polyacrylamide gel electrophoresis and subsequently transferred onto polyvinylidene fluoride membranes (Millipore, IPVH0010). After a 1-hour blocking step with 5% nonfat milk, the membranes were incubated with primary antibodies against MGP (Proteintech 600551-Ig), FABP3 (ABclonal A5312), LPL (Abcam ab168773), CEBPα (Abcam ab40761), Perilipin 1 (CST 3470), CaMKII (Abcam ab52476), P-CaMKII (CST D21E4), RIP140 (Santa Cruz Biotechnology 2656C6a) or GAPDH (CST 5174) at 4 °C overnight. Then, the membranes were washed three times with Tris-buffered saline-Tween (TBST), followed by a 1-hour incubation with HRP-conjugated secondary antibodies. Detection was carried out using Immobilon Western Chemiluminescent HRP Substrate (Millipore WBKLS0500). Densitometric analysis was conducted using ImageJ software.

### Real-time quantitative reverse transcription‒polymerase chain reaction (qRT‒PCR)

For quantification of the mRNA expression levels of the target genes in our study, SYBR Premix Ex Taq (TaKaRa) and a real-time fluorescence quantitative PCR system (Applied Biosystems, 7500) were used. Total RNA was isolated from MSCs using TRIzol (Invitrogen) following the manufacturer’s instructions. Subsequently, cDNA synthesis was carried out using a PrimeScriptTM RT Reagent Kit (TaKaRa). The relative gene expression levels were normalized to those of GAPDH utilizing the 2^−ΔΔCT^ method. The primer sequences used for this investigation are shown in Supplementary Table S2.

### Lentiviral construction and infection

The procedures for constructing and infecting lentiviruses have been previously described [50]. The lentiviruses used in the experiment were synthesized by OBiO Technology (Shanghai, China). The overexpression lentiviruses (OE-MGP, OEFABP3, and OE-RIP140) and their corresponding vector controls were generated. The constructed lentiviruses encoding shRNA (sh-MGP, sh-FABP3, sh-RIP140) and negative control shRNA were also purchased from OBiO Technology (Shanghai, China). MSCs were transduced with the MGP-overexpressing lentivirus or vector (MOI: 50) in the presence of 5 µg/ml polybrene. The medium containing the lentivirus was replaced with fresh medium after 24 h. The sequences for the target and negative control shRNAs are provided in Supplementary Table S2.

### Measurement of intracellular free Ca2+ by fluorescence microscopy

Intracellular free calcium was measured by Fluo 4-AM (Beyotime S1060). Briefly, MSCs were incubated with Fluo-4 AM dissolved in DMSO to a final concentration of 10 μM for 20 min at room temperature, after which the Fluo-4-AM was allowed to fully enter the cells. Finally, the cells were examined under a fluorescence microscope (Leica DMI4000 B) at an excitation wavelength of 488 nm and an emission wavelength of 520 nm and analyzed with ImageJ software.

### Determination of the intracellular calcium concentration by flow cytometry

MSCs were transfected with sh-MGP lentivirus or vector for 24 h. Subsequently, the culture medium was changed to adipogenesis-inducing medium. After 3 days of adipogenic induction, the cells were washed three times with HBSS solution. The cells were treated with Fluo-4 AM working solution, ensuring complete coverage, and then incubated at 37 °C and 5% CO2 for 30 min. After incubation, the Fluo-4 AM working solution was removed, and the cells were suspended in HBSS solution. The cells were promptly analyzed using flow cytometry. Doublet exclusion was performed by analyzing the two-dimensional dot plot FSC-A vs. FSC-H. The mean intensity of fluorescence was determined using FlowJo software.

### CUT&Tag-qPCR

A NovoNGS CUT&Tag 3.0 High-Sensitivity Kit (Novoprotein) was used to study DNA‒protein interactions. A total of 1.0 × 10^5^ MSCs were obtained through digestion with trypsin and subsequently incubated with ConA beads for 10 minutes. The RIP140 antibody RIP140 (Santa Cruz Biotechnology 2656C6a) was diluted at a ratio of 1:50 and incubated overnight at 4 °C. The samples were incubated with anti-mouse IgG antibody buffer for 1 h, followed by three washes and subsequent treatment with Protein A/G-Tn5. The tagment process was activated by mixing ChiTaq Buffer with MgCl2 at room temperature for 1 h, and DNA extraction was performed using Tagment DNA Extract Beads. The extracted DNA was purified by DNA clean beads and utilized for CUT&Tag-qPCR analysis. The obtained data were normalized to that of the IgG control. The primers are listed in Supplementary Table S2.

### Dual-luciferase reporter assay

The promoter sequence of FABP3 from −2000 bp relative to the transcription start site and the antisense sequence were synthesized and individually inserted into a pGL4.10 vector. Subsequently, 293T cells were transfected with the indicated vectors, followed by transduction with lentiviruses for RIP140 silencing or overexpression using Lipofectamine™ RNAiMAX (Invitrogen). Luciferase activity was quantified using a dual luciferase reporter assay kit (Yeasen 11402ES60), with Renilla luciferase serving as an internal control for normalizing luciferase activity.

### Microcomputed tomography (micro-CT)

After anesthesia and euthanasia, femurs were harvested from the different groups of C57BL/6J mice and fixed in 4% polyoxymethylene for 36 h. Imaging was conducted with a Micro-CT system (Siemens) at each of the 360 rotational steps, employing a pixel size of 8.82 lm, a voltage of 80 kV, a current of 500 lA, and an exposure time of 1500 ms. Micro-CT analysis, adhering to the guidelines established by the American Society for Bone and Mineral Research, was performed using the Inveon Research Workplace (Siemens) to quantify various parameters, including bone volume/total volume (BV/TV), Bone surface area/bone volume (BS/BV), trabecular separation (Tb.Sp), cortical thickness (Ct.Th), trabecular number (Tb.N) and trabecular thickness (Tb.Th) of femurs. Furthermore, three-dimensional structures were generated for 100 scanning layers of the distal femur approximately 50 mm below the growth plate through the reconstruction of image slices.

### Hematoxylin and eosin (H&E), immunohistochemical (IHC) staining

The tissue sections were subjected to various preparation techniques for staining, depending on the staining method employed. For H&E staining, sections were initially deparaffinized in xylene, followed by hydration with decreasing ethanol concentrations. Subsequently, the sections were treated with haematoxylin for 8 min and eosin for 3 min after clearing with a differentiation solution (1% HCl and 70% alcohol).

For MGP IHC staining, the sections were subjected to antigen retrieval, blocked in 5% goat serum, and then incubated with anti-MGP (Proteintech 60055-1-Ig). Next, the sections were exposed to the appropriate biotin-conjugated secondary antibody and subsequently treated with a DAB solution, followed by color development. After staining, all sections were dehydrated with increasing concentrations of ethanol and xylene. Finally, all sections were visualized and photographed under a light microscope (Leica, Germany).

### Immunofluorescence staining

For immunofluorescence staining of tissue sections, the sections were first subjected to deparaffinization‌ and rehydration. Then the sections were permeabilized using 1% Triton X-100 for 15 min, followed by antigen retrieval in citrate buffer. For immunofluorescence staining of hMSCs, Cultured cells were fixed in 4% paraformaldehyde for 30 min, permeabilized with 0.5% Triton X-100 for 15 min After blocking with 5% normal goat serum for 30 min at room temperature, bone sections or hMSCs were incubated with the primary antibody (anti-MGP, Proteintech 60055-1-Ig) overnight at 4 °C. The next day, the sections or hMSCs were incubated with a fluorescein-conjugated secondary antibody (Cell Signaling Technology 4409) for 60 min at room temperature. Nuclei were stained with DAPI. Images were captured using a fluorescence microscope (DMI4000 B).

### Statistical analysis

The statistical analysis of the data involved the following procedures. The experiments were repeated at least three times, and all the data are expressed as the mean ± standard deviation (SD). Student’s *t* test was used for two-group comparisons, while one-way ANOVA followed by the Bonferroni correction was used for comparisons involving three or more groups. The Pearson correlation test was used for correlation analyses.

The statistical analysis was performed using GraphPad Prism 9 and SPSS 22.0 software. *P* value < 0.05 was considered to indicate statistical significance.

## Supplementary information

Supplementary Table S1

Supplementary Table S2

Supplementary figure

Original western blots

## Data Availability

The research’s supporting data can be accessed by making a reasonable request to the corresponding authors.

## References

[1] Compston JE, McClung MR, Leslie WD. Osteoporosis. Lancet. 2019;393:364–76. 30696576 10.1016/S0140-6736(18)32112-3

[2] Lane NE. Epidemiology, etiology, and diagnosis of osteoporosis. Am J Obstet Gynecol. 2006;194:S3–11. 16448873 10.1016/j.ajog.2005.08.047

[3] Li J, Chen X, Lu L, Yu X. The relationship between bone marrow adipose tissue and bone metabolism in postmenopausal osteoporosis. Cytokine Growth Factor Rev. 2020;52:88–98. 32081538 10.1016/j.cytogfr.2020.02.003

[4] Chen Q, Shou P, Zheng C, Jiang M, Cao G, Yang Q, et al. Fate decision of mesenchymal stem cells: adipocytes or osteoblasts? Cell Death Differ. 2016;23:1128–39. 26868907 10.1038/cdd.2015.168 PMC4946886

[5] Xie Q, Liu R, Jiang J, Peng J, Yang C, Zhang W, et al. What is the impact of human umbilical cord mesenchymal stem cell transplantation on clinical treatment? Stem Cell Res Ther. 2020;11:519. 33261658 10.1186/s13287-020-02011-z PMC7705855

[6] Palmer AK, Jensen MD. Metabolic changes in aging humans: current evidence and therapeutic strategies. J Clin Invest. 2022;132:e158451. 10.1172/JCI158451 PMC9374375 35968789

[7] Bethel M, Chitteti BR, Srour EF, Kacena MA. The changing balance between osteoblastogenesis and adipogenesis in aging and its impact on hematopoiesis. Curr Osteoporos Rep. 2013;11:99–106. 23423562 10.1007/s11914-013-0135-6 PMC3643998

[8] Li SN, Wu JF. TGF-beta/SMAD signaling regulation of mesenchymal stem cells in adipocyte commitment. Stem Cell Res Ther. 2020;11:41. 31996252 10.1186/s13287-020-1552-y PMC6990519

[9] Han L, Wang B, Wang R, Gong S, Chen G, Xu W. The shift in the balance between osteoblastogenesis and adipogenesis of mesenchymal stem cells mediated by glucocorticoid receptor. Stem Cell Res Ther. 2019;10:377. 31805987 10.1186/s13287-019-1498-0 PMC6896503

[10] Devlin MJ, Rosen CJ. The bone-fat interface: basic and clinical implications of marrow adiposity. Lancet Diab Endocrinol. 2015;3:141–7. 10.1016/S2213-8587(14)70007-5 PMC4138282 24731667

[11] Price PA, Williamson MK. Primary structure of bovine matrix Gla protein, a new vitamin K-dependent bone protein. J Biol Chem. 1985;260:14971–5. 3877721

[12] Schurgers LJ, Uitto J, Reutelingsperger CP. Vitamin K-dependent carboxylation of matrix Gla-protein: a crucial switch to control ectopic mineralization. Trends Mol Med. 2013;19:217–26. 23375872 10.1016/j.molmed.2012.12.008

[13] Poterucha TJ, Goldhaber SZ. Warfarin and Vascular Calcification. Am J Med. 2016;129:635.e1–4. 26714212 10.1016/j.amjmed.2015.11.032

[14] Wang M, Chen L, Chen Y, Wei R, Guo Q, Zhu S, et al. Intracellular matrix Gla protein promotes tumor progression by activating JAK2/STAT5 signaling in gastric cancer. Mol Oncol. 2020;14:1045–58. 32086862 10.1002/1878-0261.12652 PMC7191194

[15] Velleman SG, Sporer KR, Ernst CW, Reed KM, Strasburg GM. Versican, matrix Gla protein, and death-associated protein expression affect muscle satellite cell proliferation and differentiation. Poult Sci. 2012;91:1964–73. 22802192 10.3382/ps.2012-02147

[16] Yuan W, Liu W, Zhang Y, Wang X, Xu C, Li Q, et al. Reduced APPL1 impairs osteogenic differentiation of mesenchymal stem cells by facilitating MGP expression to disrupt the BMP2 pathway in osteoporosis. J Biol Chem. 2023;299:104823. 37187293 10.1016/j.jbc.2023.104823 PMC10318529

[17] Li C, Li J, He F, Li K, Li X, Zhang Y. Matrix Gla protein regulates adipogenesis and is serum marker of visceral adiposity. Adipocyte. 2020;9:68–76. 32000575 10.1080/21623945.2020.1721692 PMC6999844

[18] Mutch DM, Rouault C, Keophiphath M, Lacasa D, Clement K. Using gene expression to predict the secretome of differentiating human preadipocytes. Int J Obes. 2009;33:354–63. 10.1038/ijo.2009.3 19223850

[19] Hackeng TM, Rosing J, Spronk HM, Vermeer C. Total chemical synthesis of human matrix Gla protein. Protein Sci. 2001;10:864–70. 11274477 10.1110/ps.44701 PMC2373974

[20] Hashimoto R, Katoh Y, Miyamoto Y, Itoh S, Daida H, Nakazato Y, et al. Increased extracellular and intracellular Ca(2)(+) lead to adipocyte accumulation in bone marrow stromal cells by different mechanisms. Biochem Biophys Res Commun. 2015;457:647–52. 25603052 10.1016/j.bbrc.2015.01.042

[21] Shi H, Halvorsen YD, Ellis PN, Wilkison WO, Zemel MB. Role of intracellular calcium in human adipocyte differentiation. Physiol Genomics. 2000;3:75–82. 11015602 10.1152/physiolgenomics.2000.3.2.75

[22] Huang X, Zhou Y, Sun Y, Wang Q. Intestinal fatty acid binding protein: A rising therapeutic target in lipid metabolism. Prog Lipid Res. 2022;87:101178. 35780915 10.1016/j.plipres.2022.101178

[23] Bensaad K, Favaro E, Lewis CA, Peck B, Lord S, Collins JM, et al. Fatty acid uptake and lipid storage induced by HIF-1alpha contribute to cell growth and survival after hypoxia-reoxygenation. Cell Rep. 2014;9:349–65. 25263561 10.1016/j.celrep.2014.08.056

[24] Rodriguez-Calvo R, Granado-Casas M, Perez-Montes de Oca A, Julian MT, Domingo M, Codina P, et al. Fatty Acid Binding Proteins 3 and 4 Predict Both All-Cause and Cardiovascular Mortality in Subjects with Chronic Heart Failure and Type 2 Diabetes Mellitus. Antioxidants. 2023;12:645. 10.3390/antiox12030645 PMC10044995 36978893

[25] Diaz P, Harris J, Rosario FJ, Powell TL, Jansson T. Increased placental fatty acid transporter 6 and binding protein 3 expression and fetal liver lipid accumulation in a mouse model of obesity in pregnancy. Am J Physiol Regul Integr Comp Physiol. 2015;309:R1569–77. 26491104 10.1152/ajpregu.00385.2015 PMC4698418

[26] Yi B, Wang J, Wang S, Yuan D, Sun J, Li Z, et al. Overexpression of Banna mini-pig inbred line fatty acid binding protein 3 promotes adipogenesis in 3T3-L1 preadipocytes. Cell Biol Int. 2014;38:918–23. 24737696 10.1002/cbin.10285

[27] Yao D, Zhao X, Zhao S, Shi H, Ma Y, Li J. Characterization of the fatty acid binding protein 3 (FABP3) promoter and its transcriptional regulation by cAMP response element binding protein 1 (CREB1) in goat mammary epithelial cells. Anim Biotechnol. 2023;34:1960–7. 35416753 10.1080/10495398.2022.2061504

[28] Scheller EL, Rosen CJ. What’s the matter with MAT? Marrow adipose tissue, metabolism, and skeletal health. Ann N Y Acad Sci. 2014;1311:14–30. 24650218 10.1111/nyas.12327 PMC4049420

[29] Seth A, Steel JH, Nichol D, Pocock V, Kumaran MK, Fritah A, et al. The transcriptional corepressor RIP140 regulates oxidative metabolism in skeletal muscle. Cell Metab. 2007;6:236–45. 17767910 10.1016/j.cmet.2007.08.004 PMC2680991

[30] Lin YW, Liu PS, Pook KA, Wei LN. Glyburide and retinoic acid synergize to promote wound healing by anti-inflammation and RIP140 degradation. Sci Rep. 2018;8:834. 29339732 10.1038/s41598-017-18785-x PMC5770422

[31] Al-Azab M, Safi M, Idiiatullina E, Al-Shaebi F, Zaky MY. Aging of mesenchymal stem cell: machinery, markers, and strategies of fighting. Cell Mol Biol Lett. 2022;27:69. 35986247 10.1186/s11658-022-00366-0 PMC9388978

[32] Yu W, Xie Z, Li J, Lin J, Su Z, Che Y, et al. Super enhancers targeting ZBTB16 in osteogenesis protect against osteoporosis. Bone Res. 2023;11:30. 37280207 10.1038/s41413-023-00267-8 PMC10244438

[33] Xie Z, Yu W, Zheng G, Li J, Cen S, Ye G, et al. TNF-alpha-mediated m(6)A modification of ELMO1 triggers directional migration of mesenchymal stem cell in ankylosing spondylitis. Nat Commun. 2021;12:5373. 34508078 10.1038/s41467-021-25710-4 PMC8433149

[34] Ye G, Li J, Yu W, Xie Z, Zheng G, Liu W, et al. ALKBH5 facilitates CYP1B1 mRNA degradation via m6A demethylation to alleviate MSC senescence and osteoarthritis progression. Exp Mol Med. 2023;55:1743–56. 37524872 10.1038/s12276-023-01059-0 PMC10474288

[35] Li J, Jin C, Gustafsson S, Rao A, Wabitsch M, Park CY, et al. Single-cell transcriptome dataset of human and mouse in vitro adipogenesis models. Sci Data. 2023;10:387. 37328521 10.1038/s41597-023-02293-x PMC10275883

[36] Hou Q, Huang Y, Liu Y, Luo Y, Wang B, Deng R, et al. Profiling the miRNA-mRNA-lncRNA interaction network in MSC osteoblast differentiation induced by (+)-cholesten-3-one. BMC Genomics. 2018;19:783. 30373531 10.1186/s12864-018-5155-2 PMC6206902

[37] Furuhashi M, Hotamisligil GS. Fatty acid-binding proteins: role in metabolic diseases and potential as drug targets. Nat Rev Drug Discov. 2008;7:489–503. 18511927 10.1038/nrd2589 PMC2821027

[38] Lee SM, Lee SH, Jung Y, Lee Y, Yoon JH, Choi JY, et al. FABP3-mediated membrane lipid saturation alters fluidity and induces ER stress in skeletal muscle with aging. Nat Commun. 2020;11:5661. 33168829 10.1038/s41467-020-19501-6 PMC7653047

[39] Blaschke M, Koepp R, Lenz C, Kruppa J, Jung K, Siggelkow H. Crohn’s disease patient serum changes protein expression in a human mesenchymal stem cell model in a linear relationship to patients’ disease stage and to bone mineral density. J Clin Transl Endocrinol. 2018;13:26–38. 30003044 10.1016/j.jcte.2018.06.002 PMC6039964

[40] Liu ZZ, Hong CG, Hu WB, Chen ML, Duan R, Li HM, et al. Autophagy receptor OPTN (optineurin) regulates mesenchymal stem cell fate and bone-fat balance during aging by clearing FABP3. Autophagy. 2021;17:2766–82. 33143524 10.1080/15548627.2020.1839286 PMC8526045

[41] Ding L, Yang X, Tian H, Liang J, Zhang F, Wang G, et al. Seipin regulates lipid homeostasis by ensuring calcium-dependent mitochondrial metabolism. EMBO J. 2018;37:e97572. 10.15252/embj.201797572 PMC6120665 30049710

[42] Griffith LC. CaMKII: new tricks for an old dog. Cell. 2008;133:397–9. 18455979 10.1016/j.cell.2008.04.018 PMC2655315

[43] Gao M, Du Y, Xie JW, Xue J, Wang YT, Qin L, et al. Redox signal-mediated TRPM2 promotes Ang II-induced adipocyte insulin resistance via Ca(2+)-dependent CaMKII/JNK cascade. Metabolism. 2018;85:313–24. 29775644 10.1016/j.metabol.2018.05.005

[44] Rong D, Sun G, Zheng Z, Liu L, Chen X, Wu F, et al. MGP promotes CD8(+) T cell exhaustion by activating the NF-kappaB pathway leading to liver metastasis of colorectal cancer. Int J Biol Sci. 2022;18:2345–61. 35414780 10.7150/ijbs.70137 PMC8990480

[45] Wang H, Goligorsky MS, Malbon CC. Temporal activation of Ca2+-calmodulin-sensitive protein kinase type II is obligate for adipogenesis. J Biol Chem. 1997;272:1817–21. 8999866 10.1074/jbc.272.3.1817

[46] Chung HT. RIP140, a Janus metabolic switch involved in defense functions. Cell Mol Immunol. 2013;10:7–9. 23241901 10.1038/cmi.2012.53 PMC4003179

[47] Lee B, Iwaniec UT, Turner RT, Lin YW, Clarke BL, Gingery A, et al. RIP140 in monocytes/macrophages regulates osteoclast differentiation and bone homeostasis. JCI Insight. 2017;2:e90517. 28405613 10.1172/jci.insight.90517 PMC5374065

[48] Song S, Guo Y, Yang Y, Fu D. Advances in pathogenesis and therapeutic strategies for osteoporosis. Pharm Ther. 2022;237:108168. 10.1016/j.pharmthera.2022.108168 35283172

[49] Liu W, Wang P, Xie Z, Wang S, Ma M, Li J, et al. Abnormal inhibition of osteoclastogenesis by mesenchymal stem cells through the miR-4284/CXCL5 axis in ankylosing spondylitis. Cell Death Dis. 2019;10:188. 30804325 10.1038/s41419-019-1448-x PMC6389901

[50] Li J, Wang P, Xie Z, Wang S, Cen S, Li M, et al. TRAF4 positively regulates the osteogenic differentiation of mesenchymal stem cells by acting as an E3 ubiquitin ligase to degrade Smurf2. Cell Death Differ. 2019;26:2652–66. 31076633 10.1038/s41418-019-0328-3 PMC7224386

