# Supplementary material for: MGP regulates the adipogenic differentiation of mesenchymal stem cells in osteoporosis via the Ca2+/CaMKII/RIP140/FABP3 axis
Source: Cell Death Discov. 2025 Apr 12;11:166. doi: 10.1038/s41420-025-02472-2 (PMC11992250; doi:10.1038/s41420-025-02472-2)
Supplement: Supplementary file 1 — Supplementary Table S1 [file 41420_2025_2472_MOESM1_ESM.docx]

| Table S1 Sequences for sh-RNA, qPCR Primer and CUT&Tag-qPCR Primer. | |  |
| --- | --- | --- |
|  |  |  |
| **Lentiviruses encoding short hairpin RNA** | **Sequences for sh-RNA(5' - 3')** |  |
| NC | TTCTCCGAACGTGTCACGTTTC |  |
| MGP | GGATACAATGCTGCCTATAAT |  |
| FABP3 | GCTAGTGGACAGCAAGAATTT |  |
| RIP140 | CCTCAGTCATGATTCTTTAAA |  |
|  |  |  |
| **qPCR Primer** | **Forward Primer(5' - 3')** | **Reverse Primer(5' - 3')** |
| MGP (human) | TCCGAGAACGCTCTAAGCCT | GCAAAGTCTGTAGTCATCACAGG |
| FABP3 (human) | GGCACCTGGAAGCTAGTGG | CTGCCTGGTAGCAAAACCC |
| GAPDH (human) | TGTGGGCATCAATGGATTTGG | ACACCATGTATTCCGGGTCAAT |
| LPL (human) | GGCCTGAAAAATGAGGACGTT | GTGCCGAAGGTCATGGTTGA |
| Perilipin1 (human) | TGTGCAATGCCTATGAGAAGG | AGGGCGGGGATCTTTTCCT |
| C/EBPα (human) | GCGGGAACGCAACAACATC | GTCACTGGTCAACTCCAGCAC |
| RIP140 (human) | AATGTGCACTTGAGCCATGATG | TCGGACACTGGTAAGGCAGG |
| MGP(mouse) | AGCCCAAAAGAGAGTCCAGGA | TGCCTGAAGTAGCGGTTGTAG |
| GAPDH(mouse) | AGGTCGGTGTGAACGGATTTG | AGGTCGGTGTGAACGGATTTG |
|  |  |  |
| **CUT&Tag-qPCR Primer** | **Forward Primer(5' - 3')** | **Reverse Primer(5' - 3')** |
| FABP3 | TGCTTTAAAAGTCCAATGAGGCCAG | TCCGGTTTCTTCGCACAGC |
