# Supplementary material for: MGP regulates the adipogenic differentiation of mesenchymal stem cells in osteoporosis via the Ca2+/CaMKII/RIP140/FABP3 axis
Source: Cell Death Discov. 2025 Apr 12;11:166. doi: 10.1038/s41420-025-02472-2 (PMC11992250; doi:10.1038/s41420-025-02472-2)
Supplement: Supplementary file 2 — Supplementary Table S2 [file 41420_2025_2472_MOESM2_ESM.docx]

Supplementary Table 2 Clinical features of control and osteoporosis subjects

| Clinical features | Control | Osteoporosis |
| --- | --- | --- |
| All subjects | 10 | 10 |
| Gender | Female | Female |
| Age (years) | 57.19±5.47 | 58.04±6.15 |
| Hight (cm) | 158.64±7.59 | 159.14±6.15 |
| Weight (kg) | 57.53±6.77 | 59.12±4.58 |
| BMI (kg/m2) | 22.82±1.80 | 23.41±2.17 |
| Menarche age(years) | 14.25±1.23 | 13.67±0.97 |
| Menopause age（yeas） | 51.59±2.27 | 49.06±2.44 |
| T score | 0.49±1.09 | -3.60±0.86 |

The data is presented as the mean ± standard deviation (SD), with a sample size of n=10 in each group.
