# Supplementary material for: MGP regulates the adipogenic differentiation of mesenchymal stem cells in osteoporosis via the Ca2+/CaMKII/RIP140/FABP3 axis
Source: Cell Death Discov. 2025 Apr 12;11:166. doi: 10.1038/s41420-025-02472-2 (PMC11992250; doi:10.1038/s41420-025-02472-2)

Figure. S1 GO analysis of the RNA sequencing data between sh-NC and sh-MGP groups. n= 3 per group.


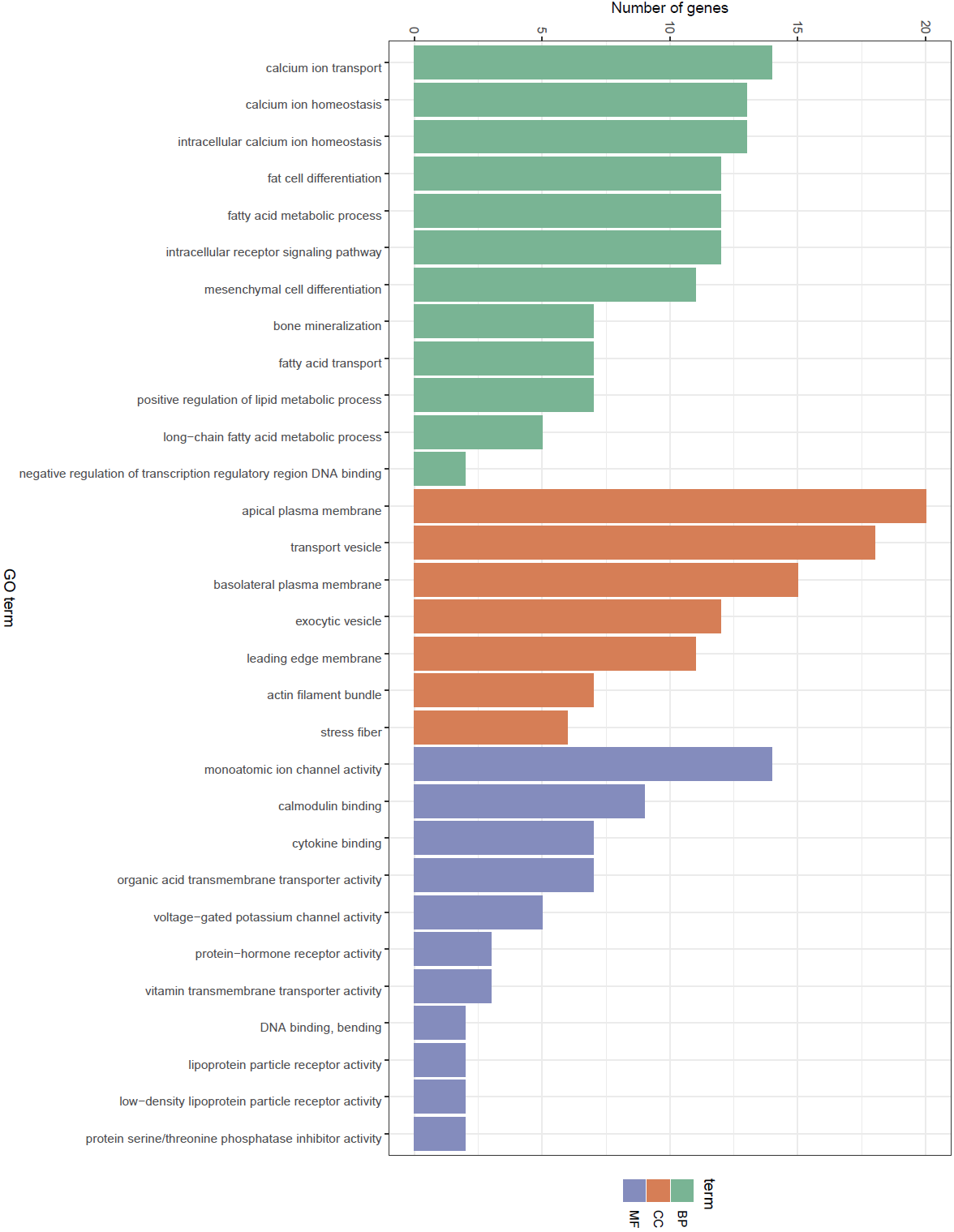


Figure. S2 KEGG pathway analysis between sh-NC and sh-MGP groups. n= 3 per group.


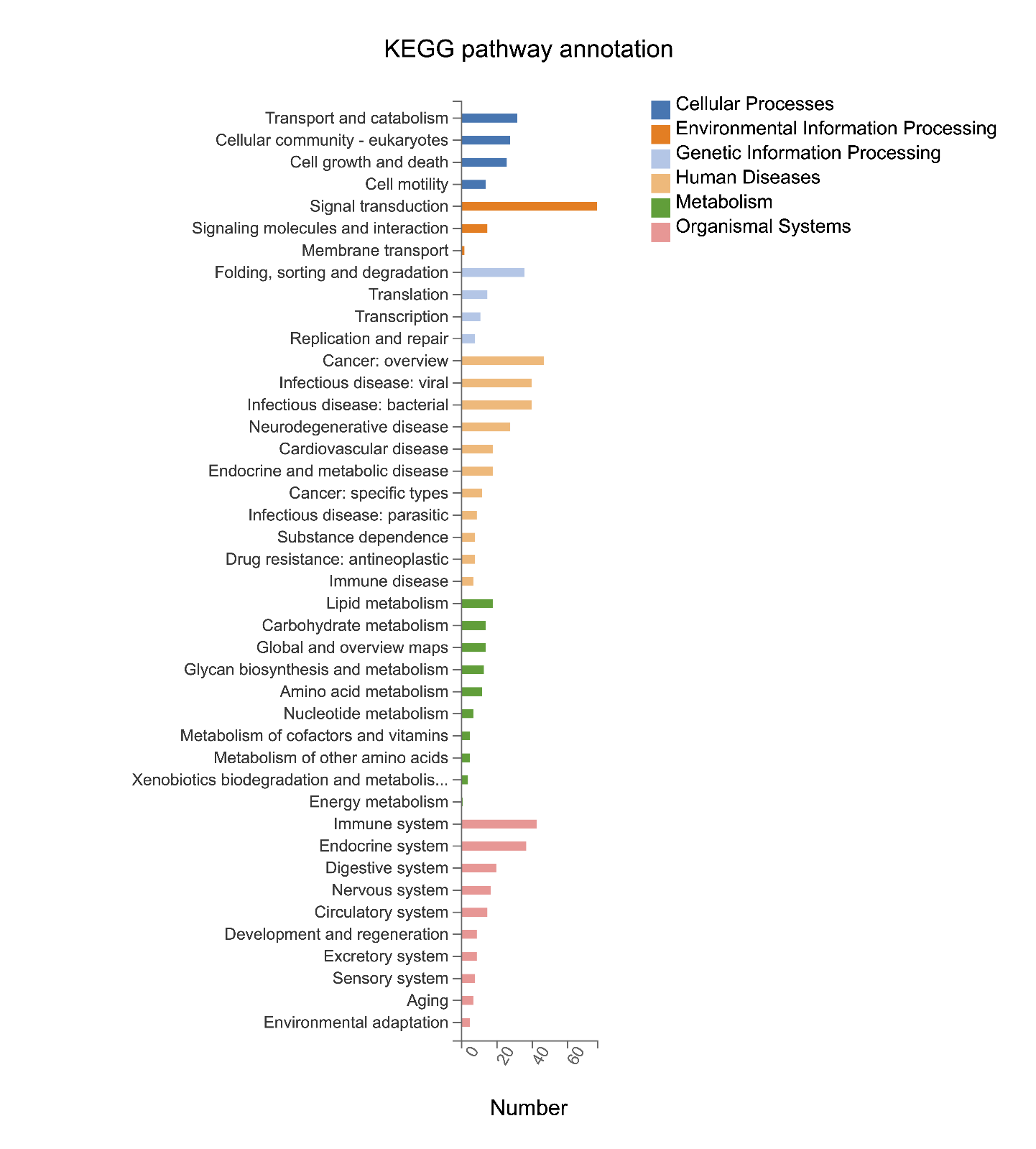


Figure. S3 The relative RNA expression of RIP140 levels was determined via qRT–

PCR are shown along with the concentration gradient of KN93. All the data are presented as the means ± SDs; n= 6 per group. ANOVA was used to determine significant differences. ns, not statistically significant.


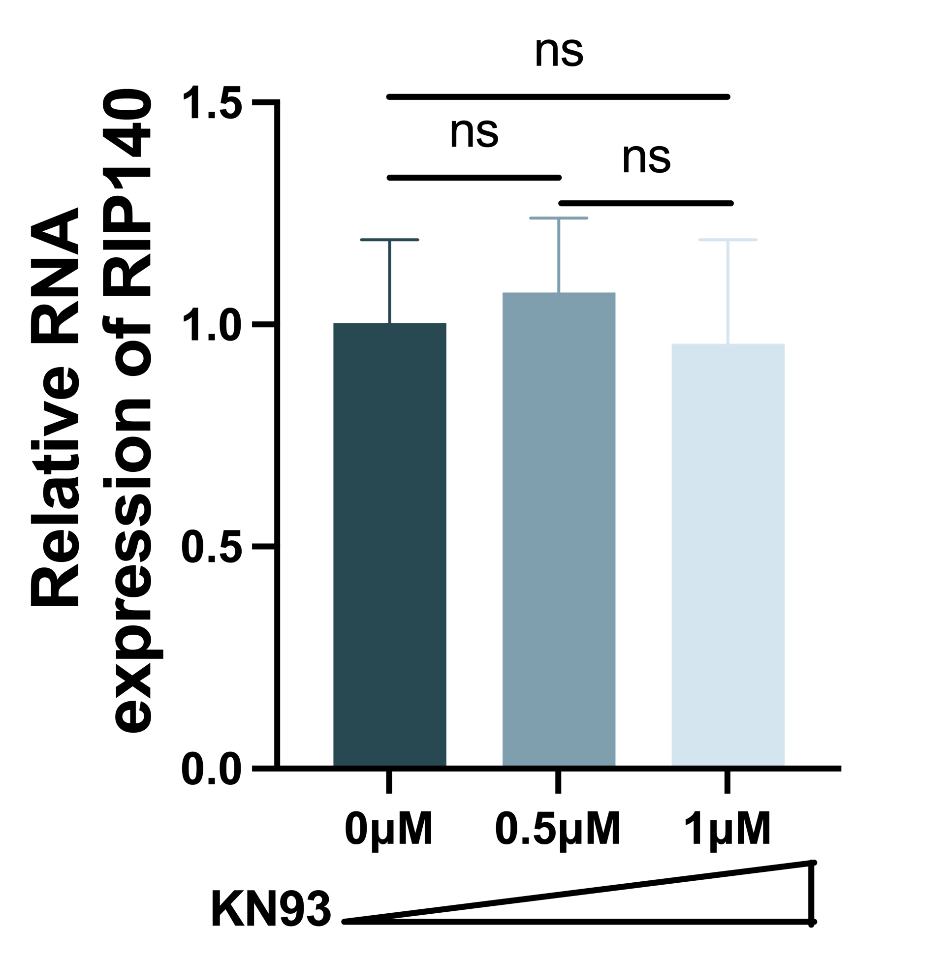


Figure. S4 Serum OCN and PINP bone formation marker levels measured by ELISA. The data are presented as the means ± SDs; n= 6 per group. ANOVA was used to determine significant differences. ns, not statistically significant; *P < 0.05; **P < 0.01.


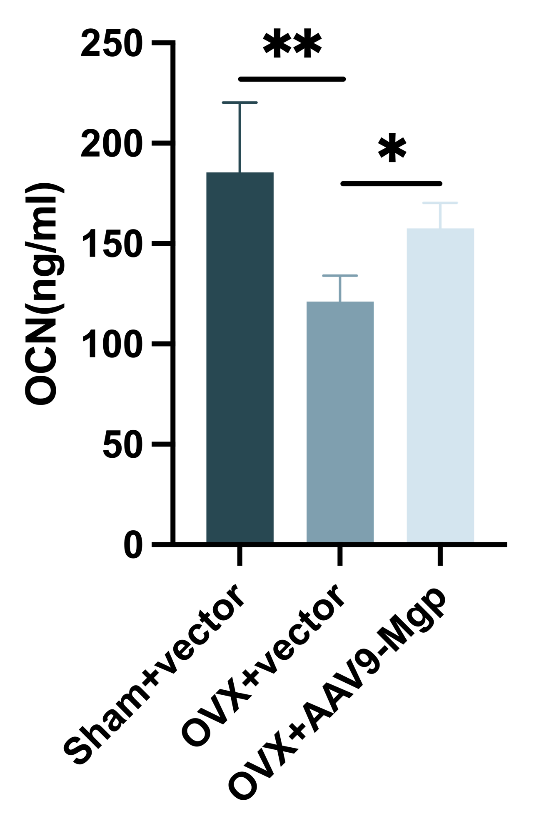

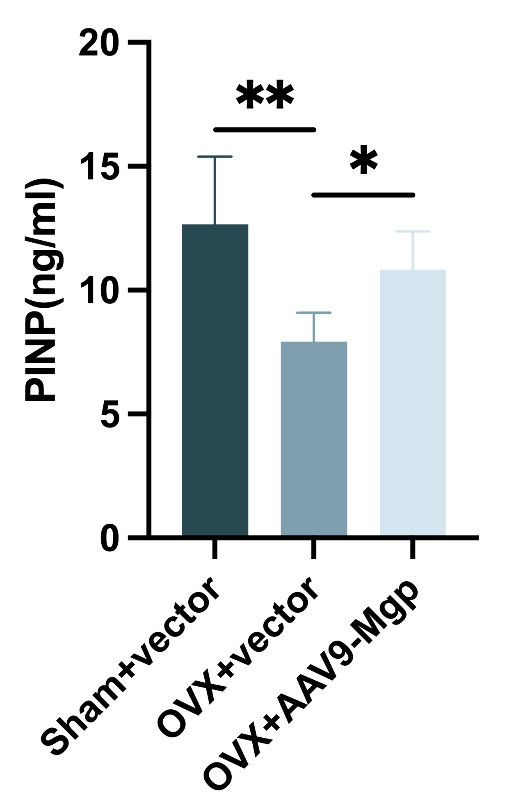

Supplement: Supplementary file 3 — Supplementary figure [file 41420_2025_2472_MOESM3_ESM.docx]
