# Supplementary material for: MGP regulates the adipogenic differentiation of mesenchymal stem cells in osteoporosis via the Ca2+/CaMKII/RIP140/FABP3 axis
Source: Cell Death Discov. 2025 Apr 12;11:166. doi: 10.1038/s41420-025-02472-2 (PMC11992250; doi:10.1038/s41420-025-02472-2)

Figure1G

MGP

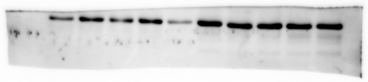

GAPDH

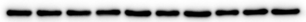

Figure1G

MGP

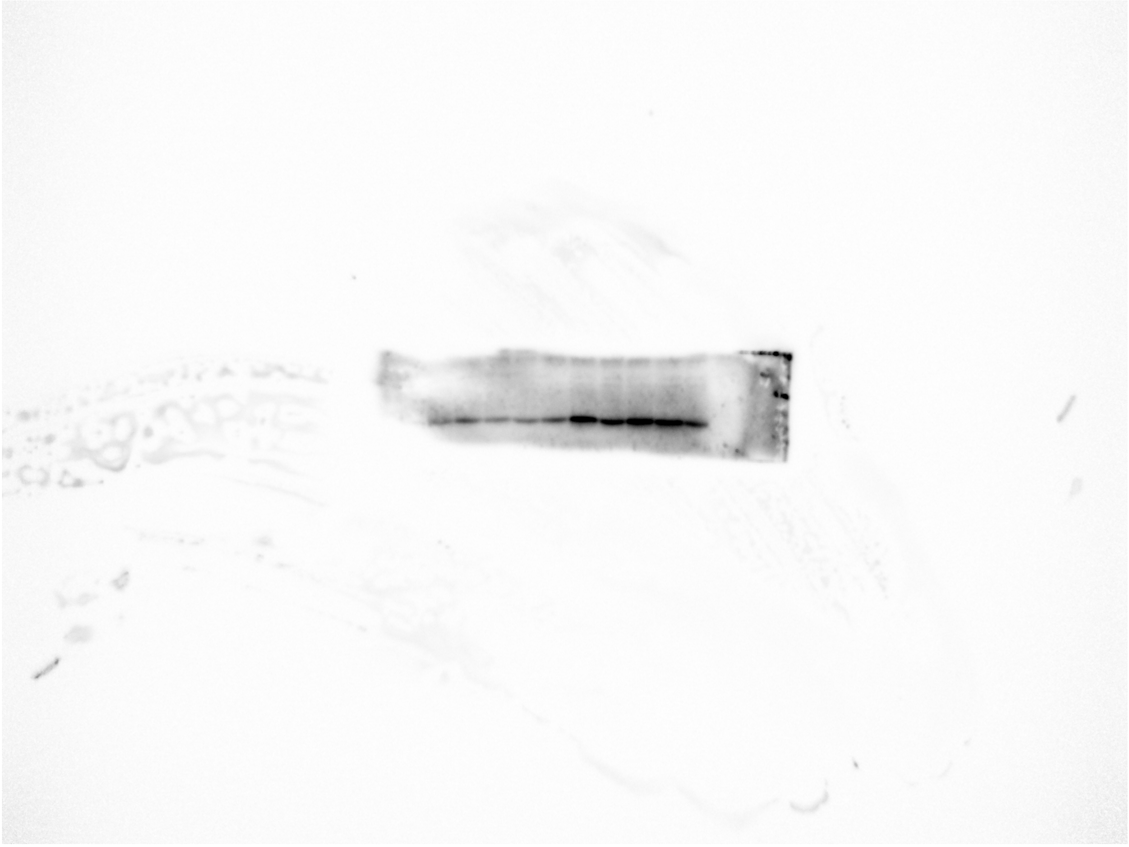

GAPDH

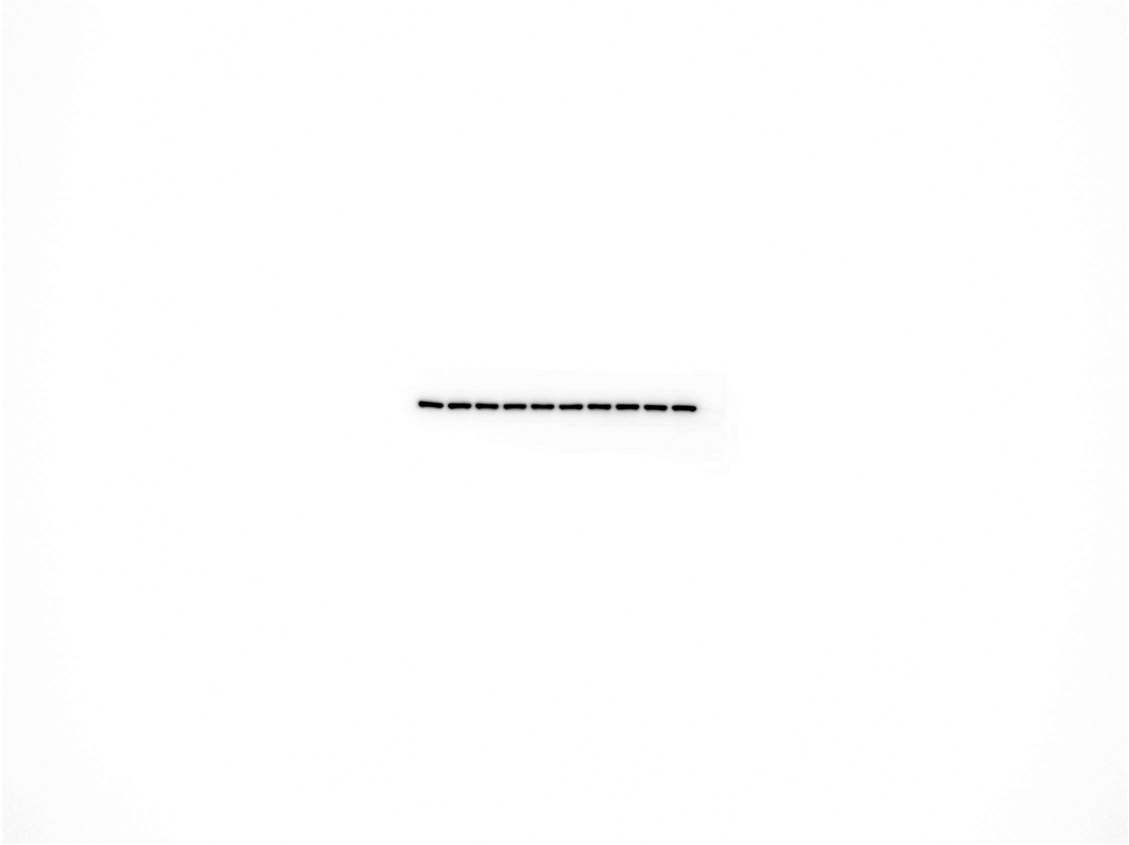

Figure2B

MGP

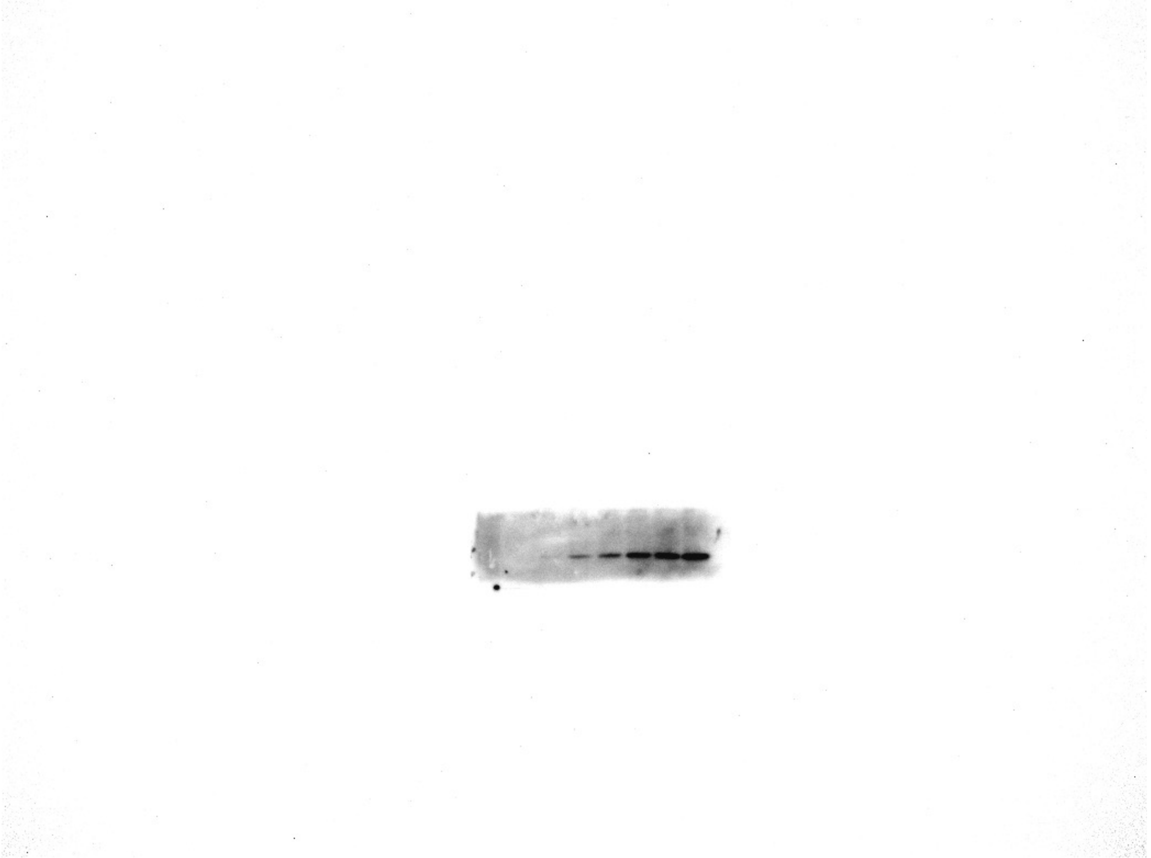

LPL

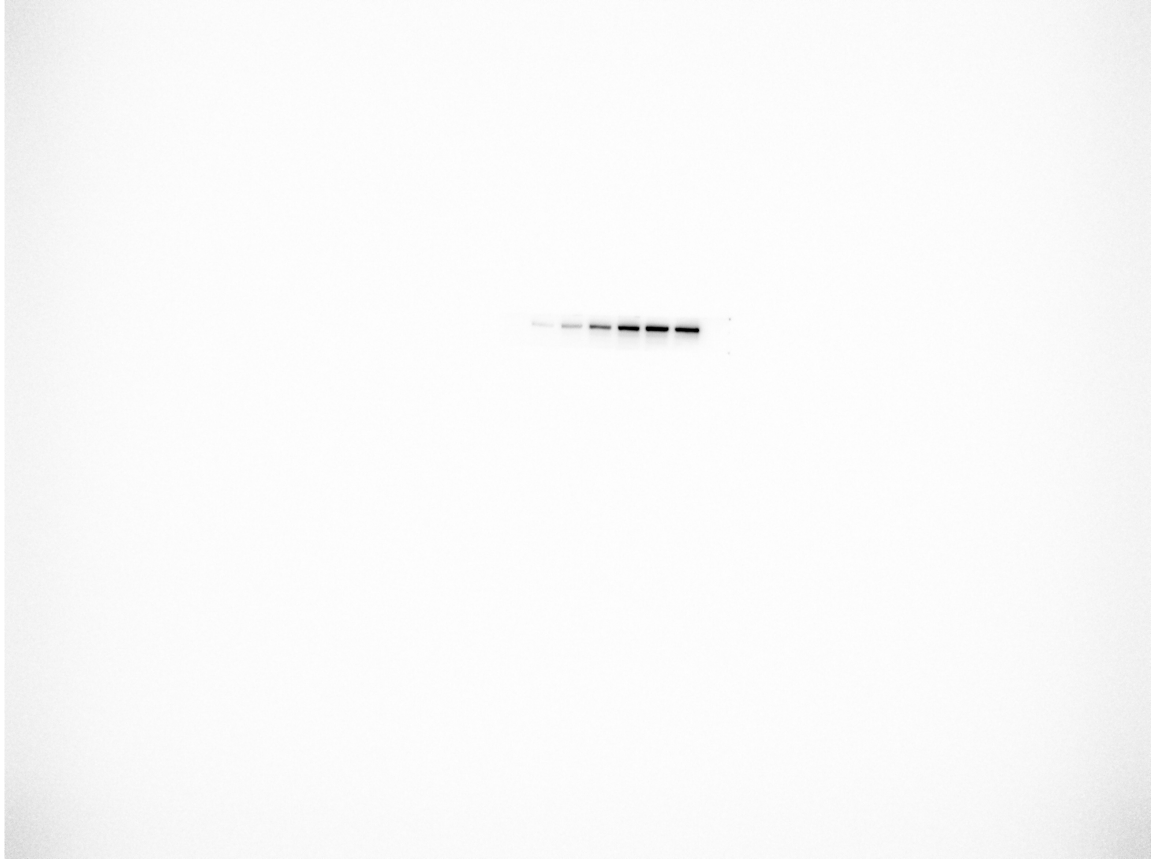

Figure2B

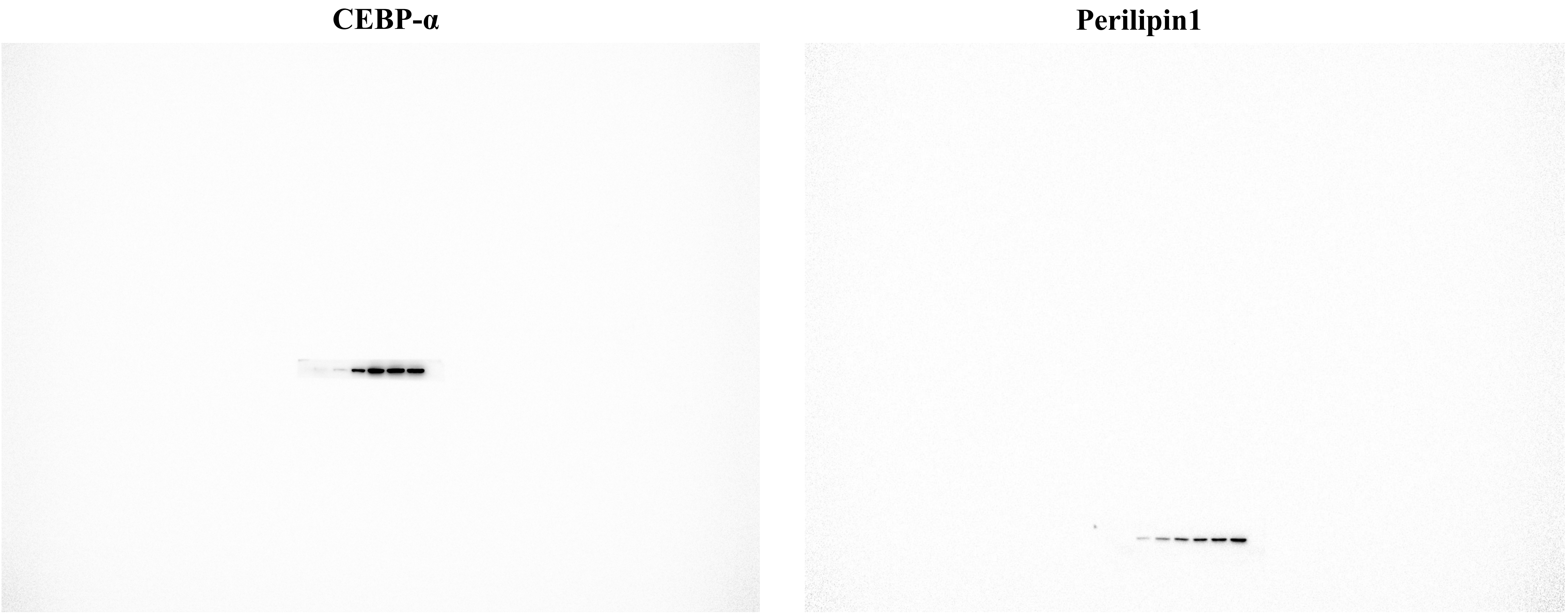

Figure2B

GAPDH

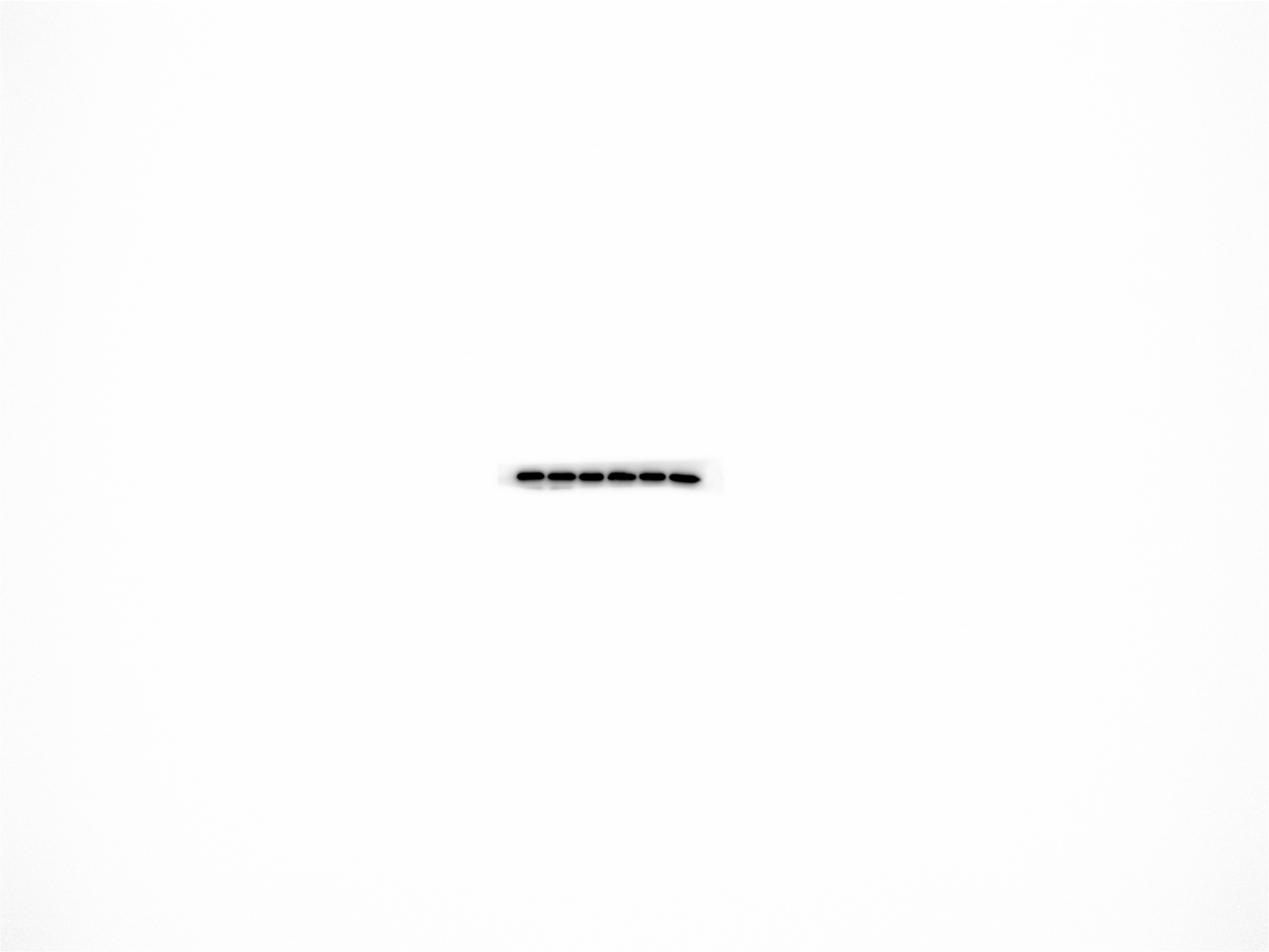

Figure2F

MGP

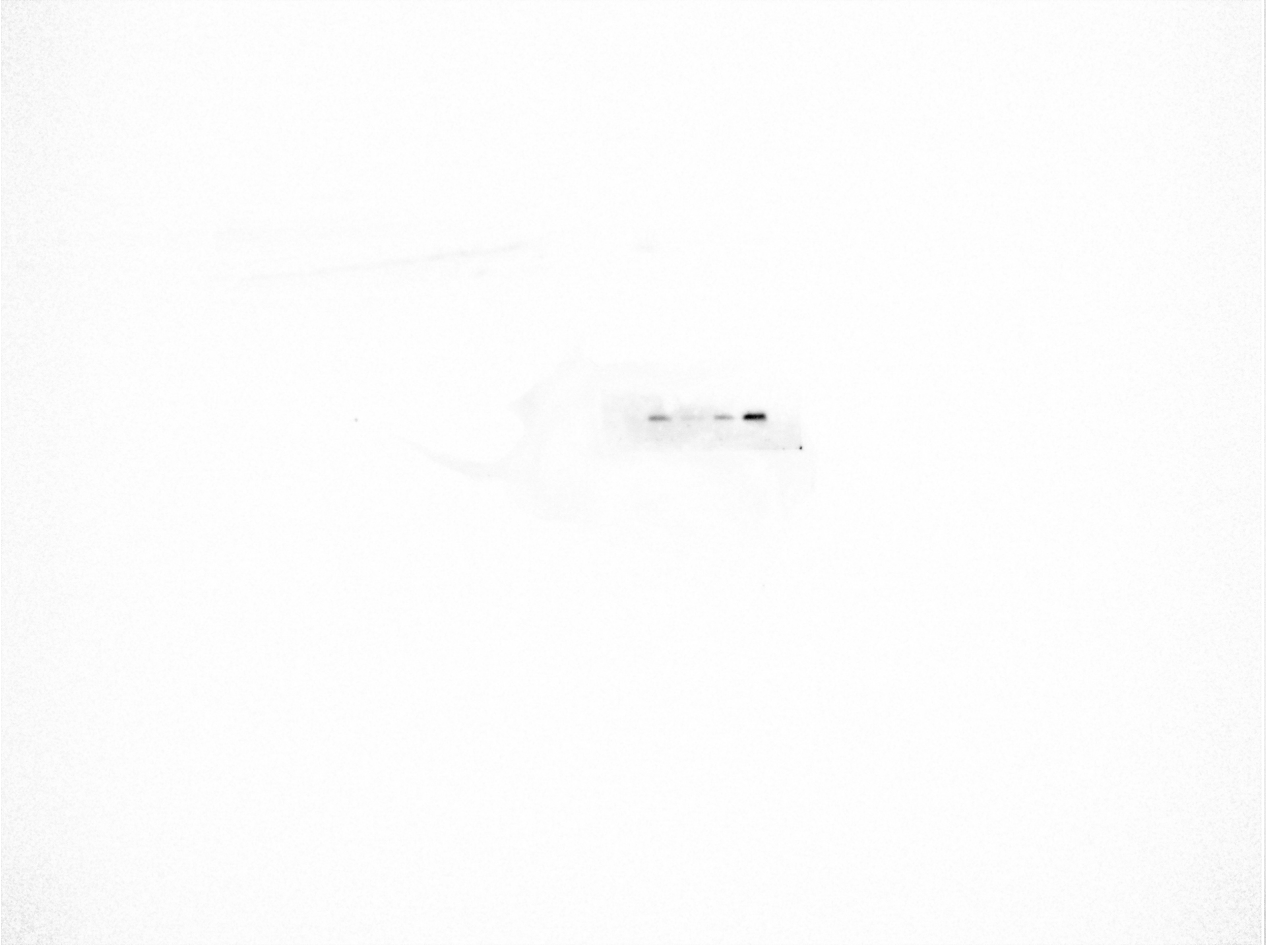

LPL

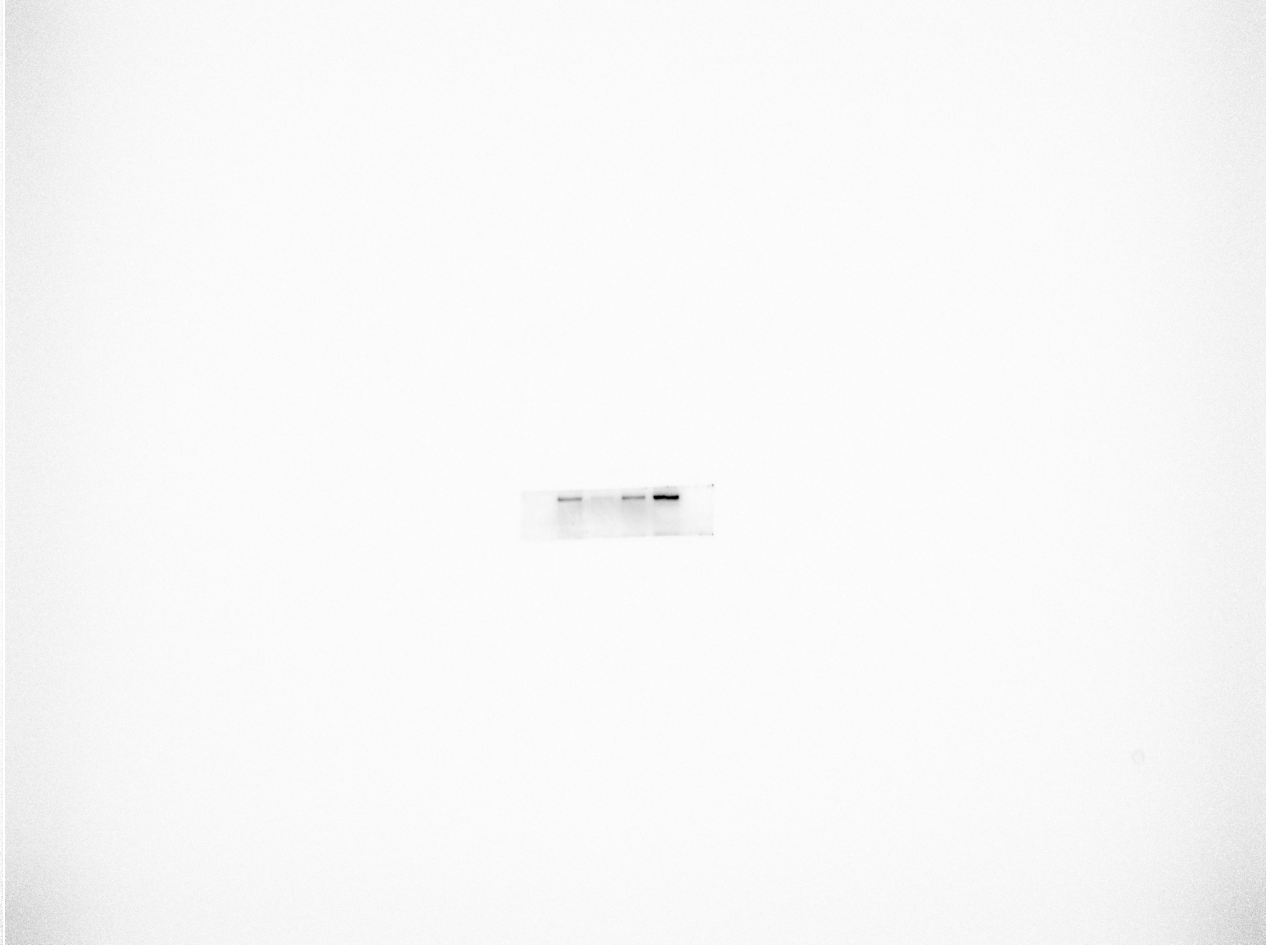

Figure2F

CEBP-α

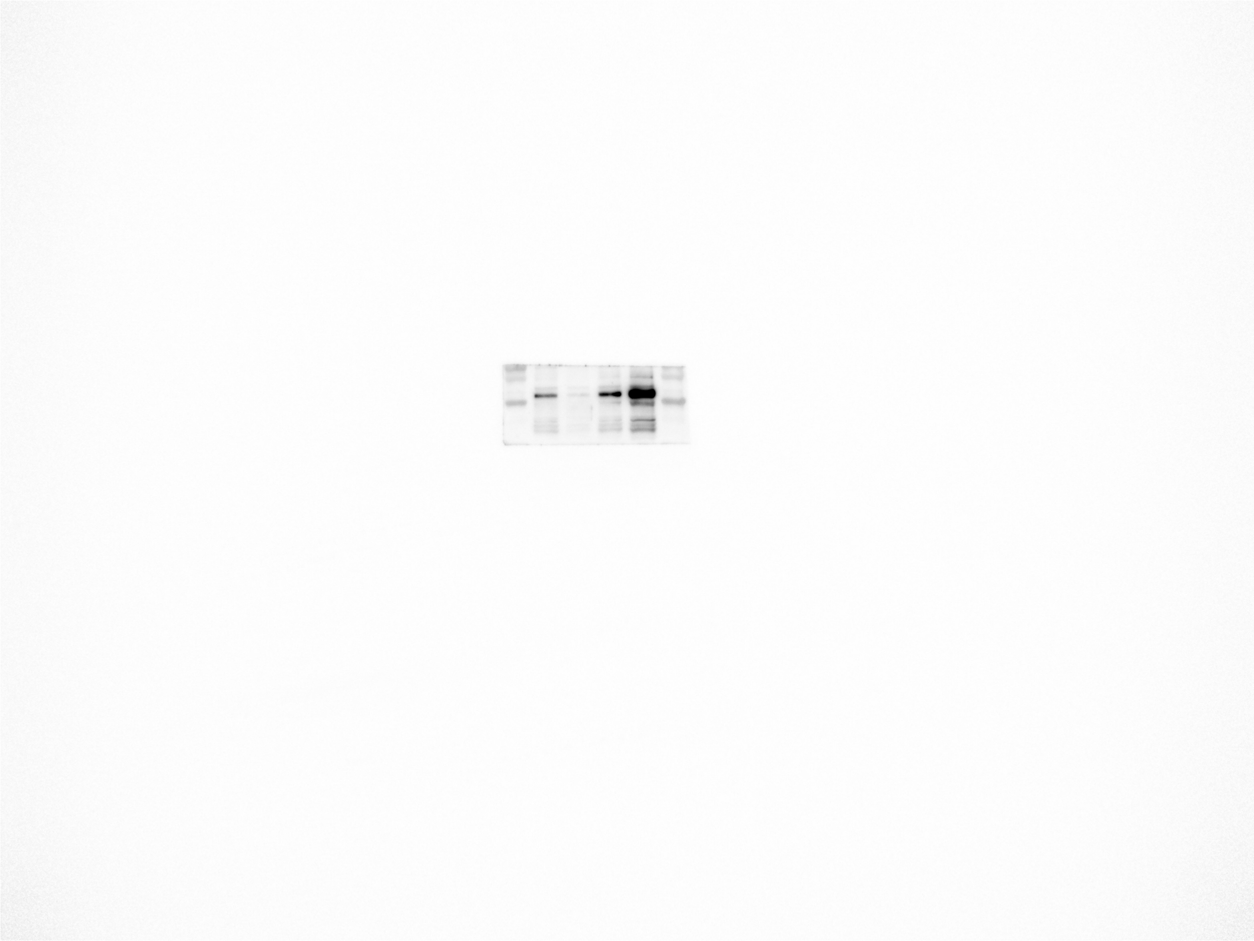

Perilipin1

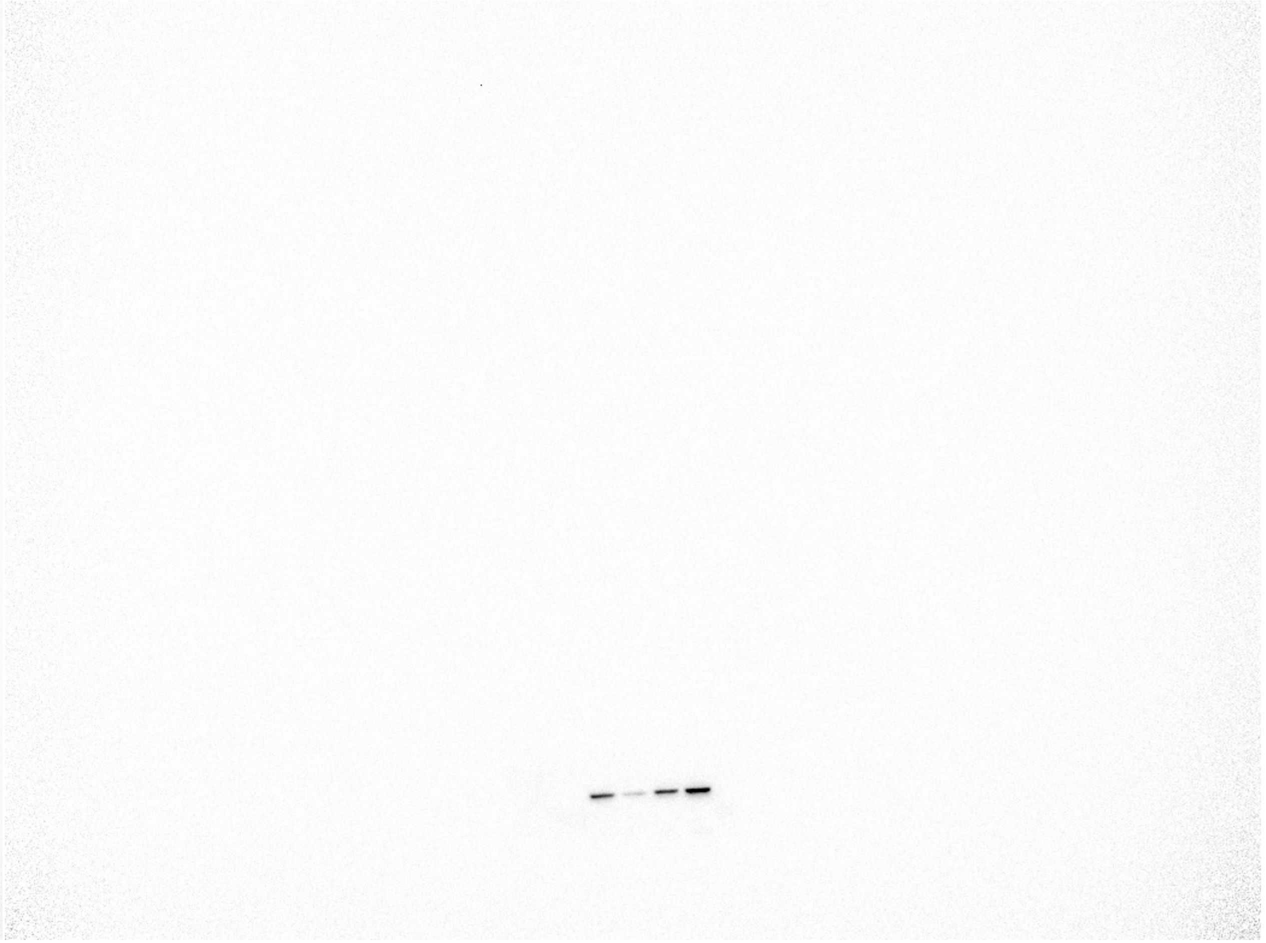

Figure2F

GAPDH

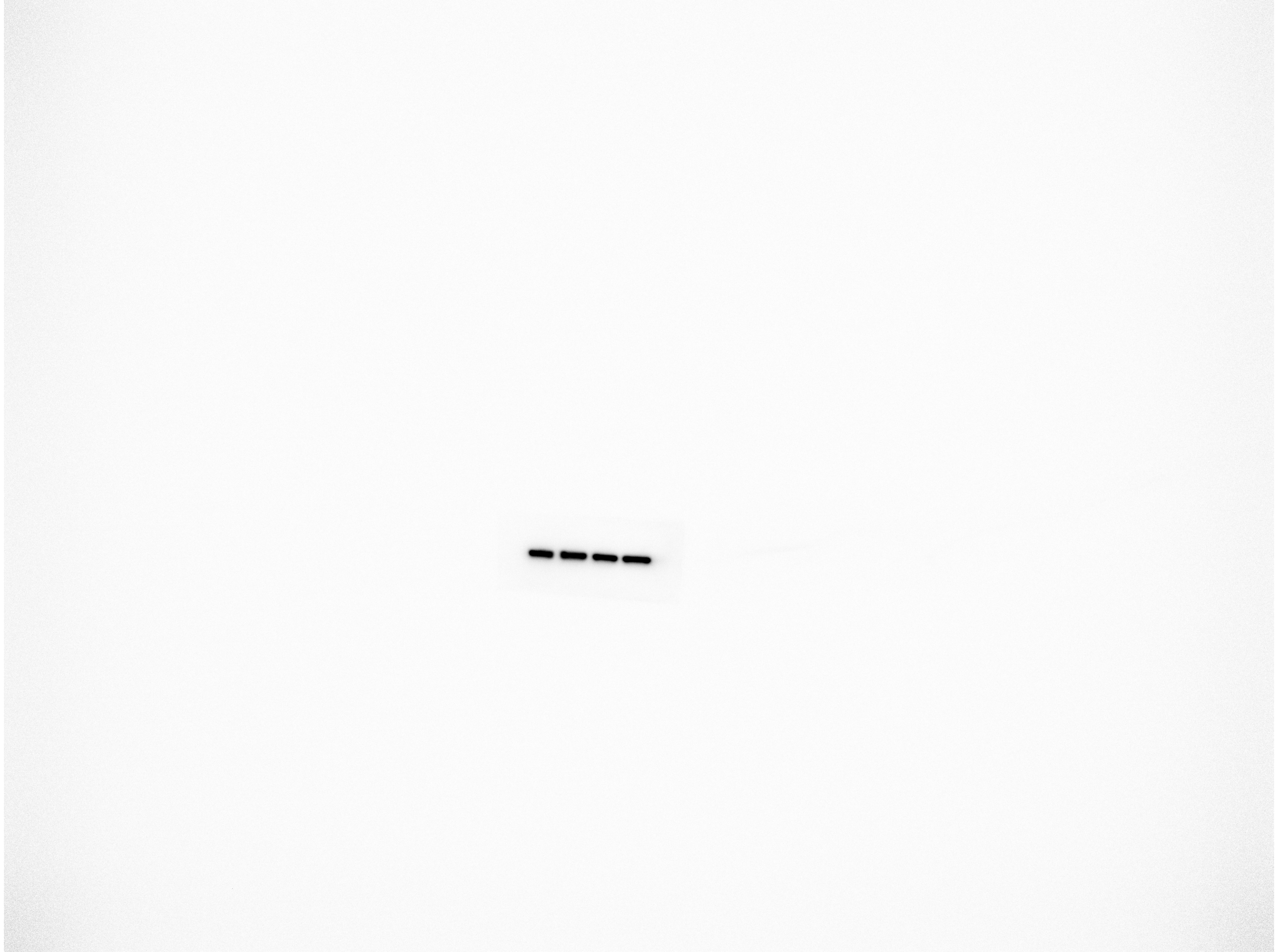

Figure3F

FABP3

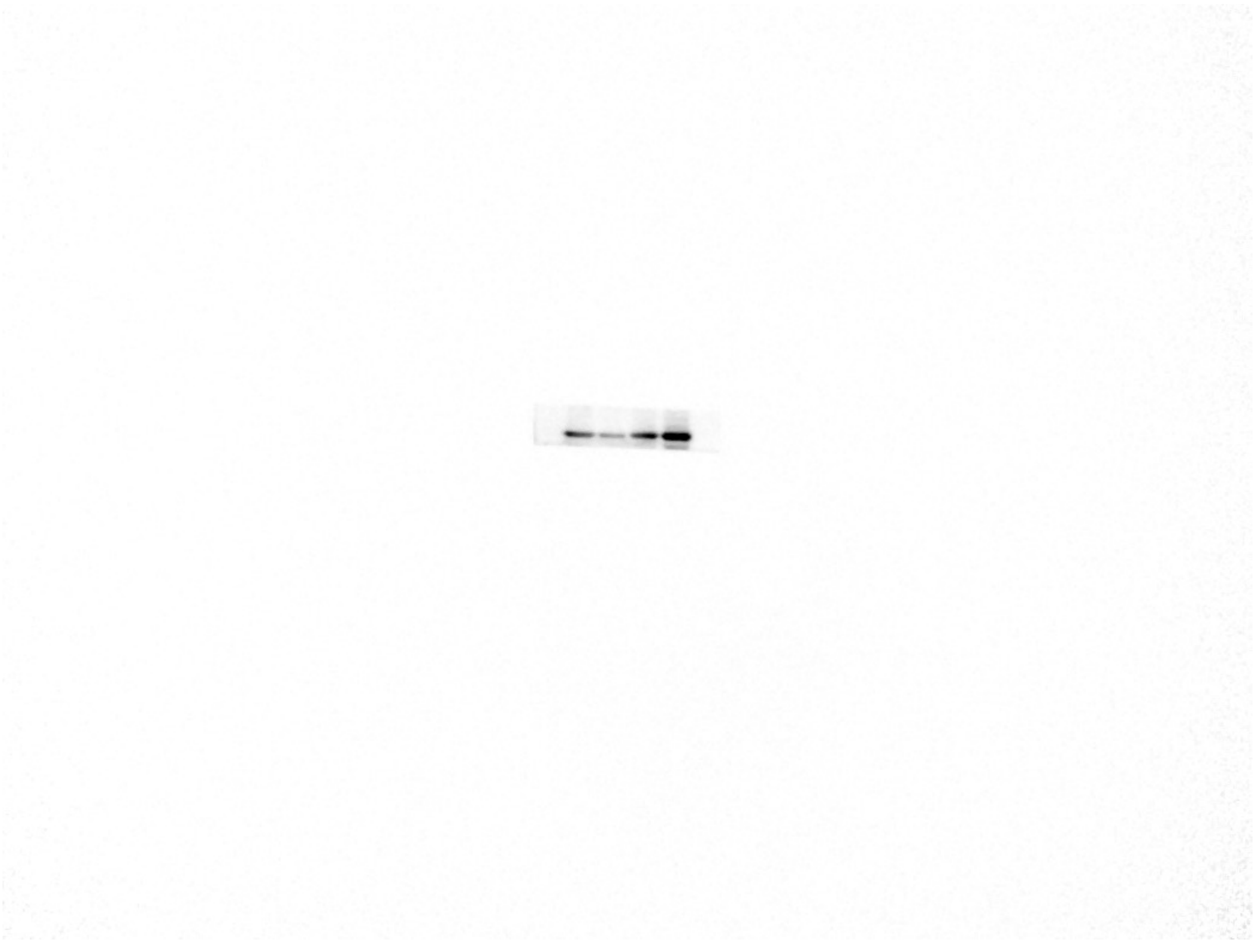

MGP

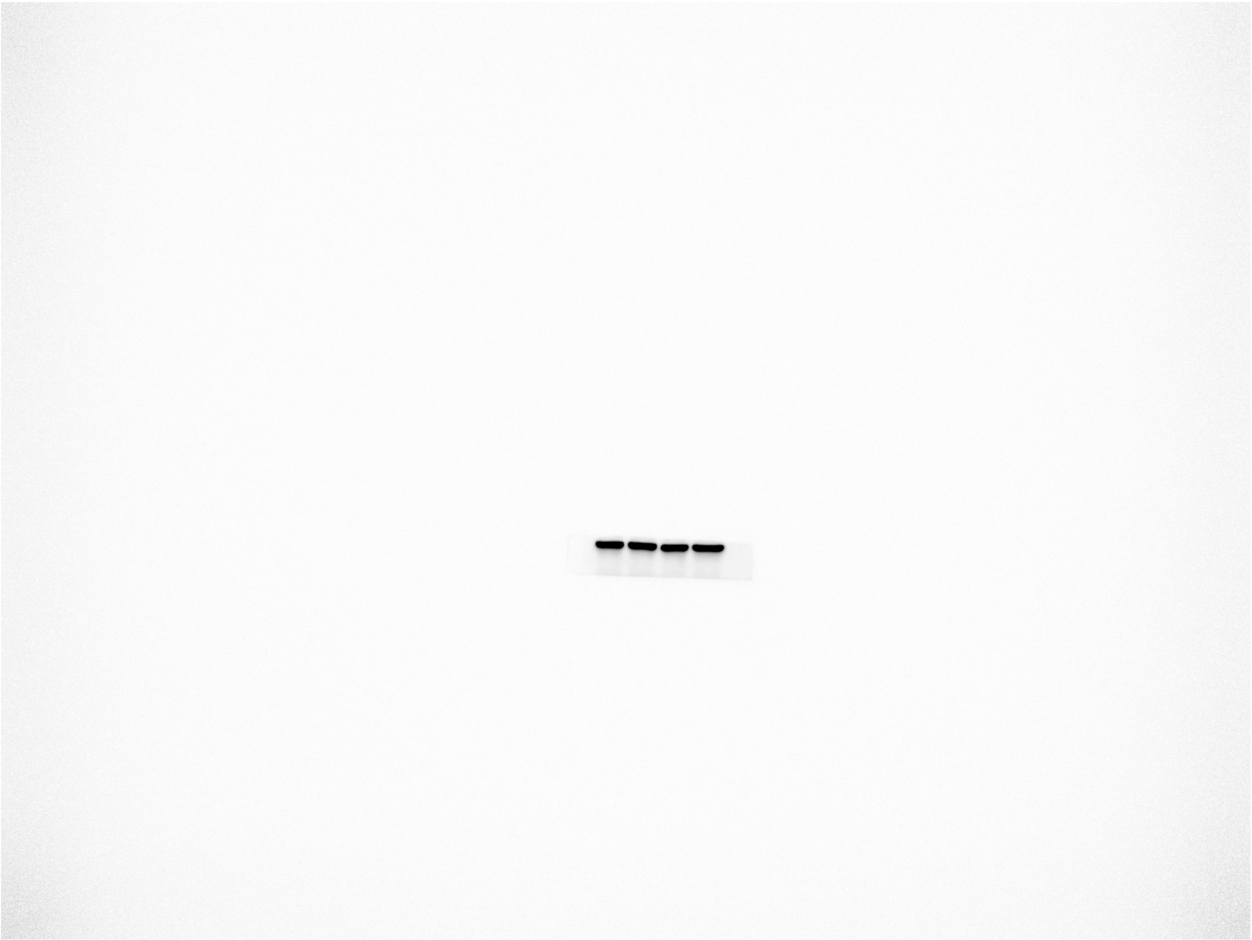

Figure3F

LPL

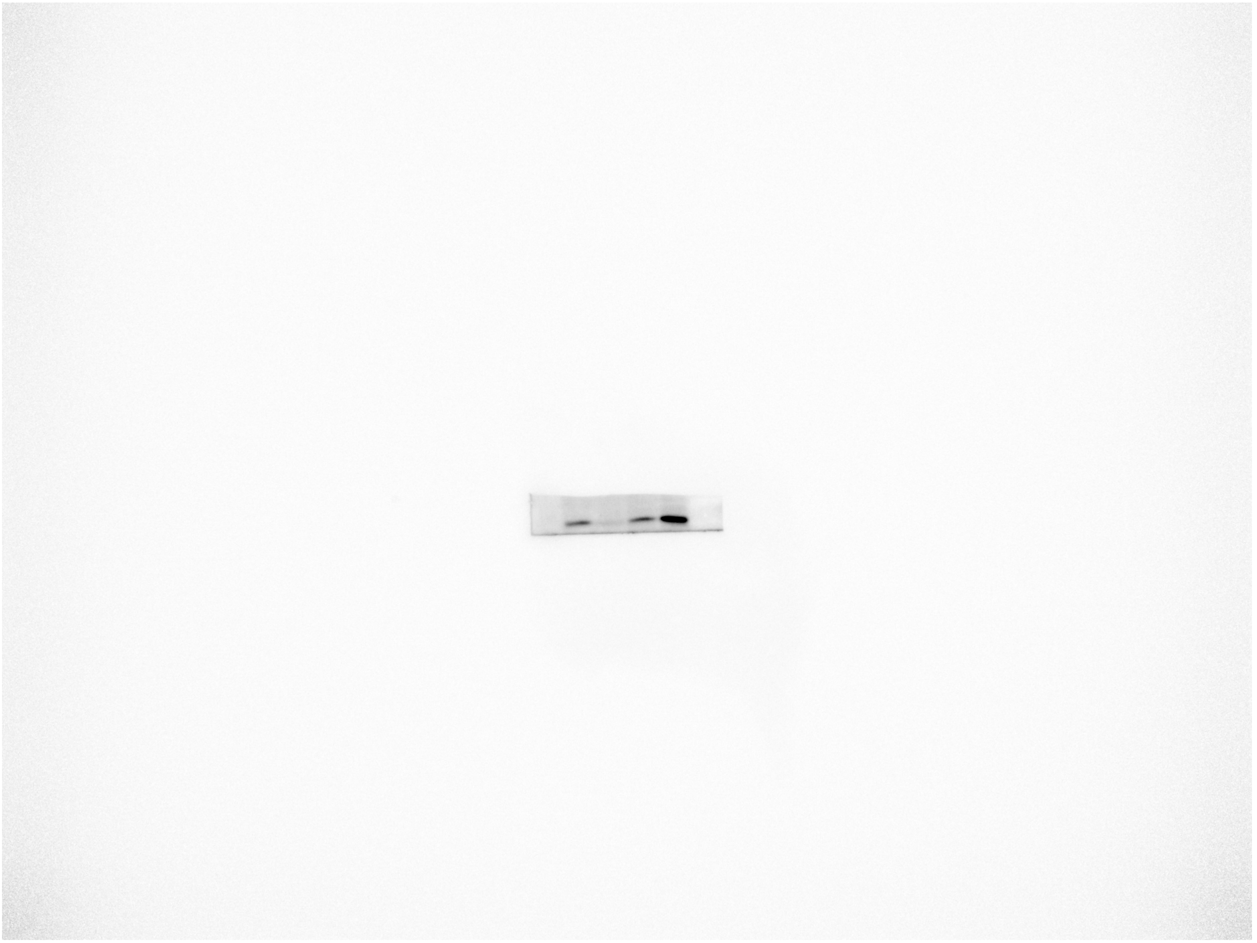

CEBP-α

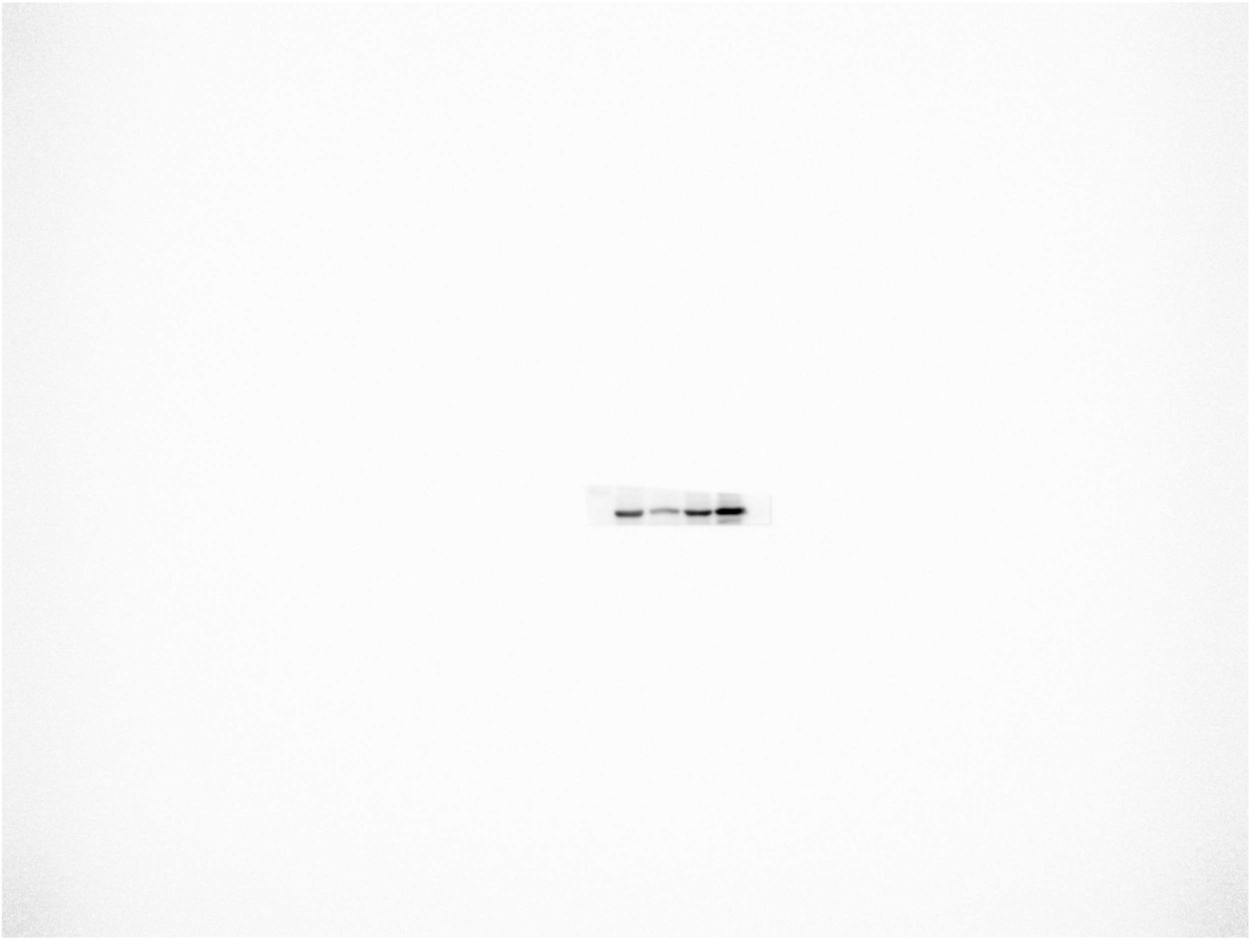

Figure3F

Perilipin1

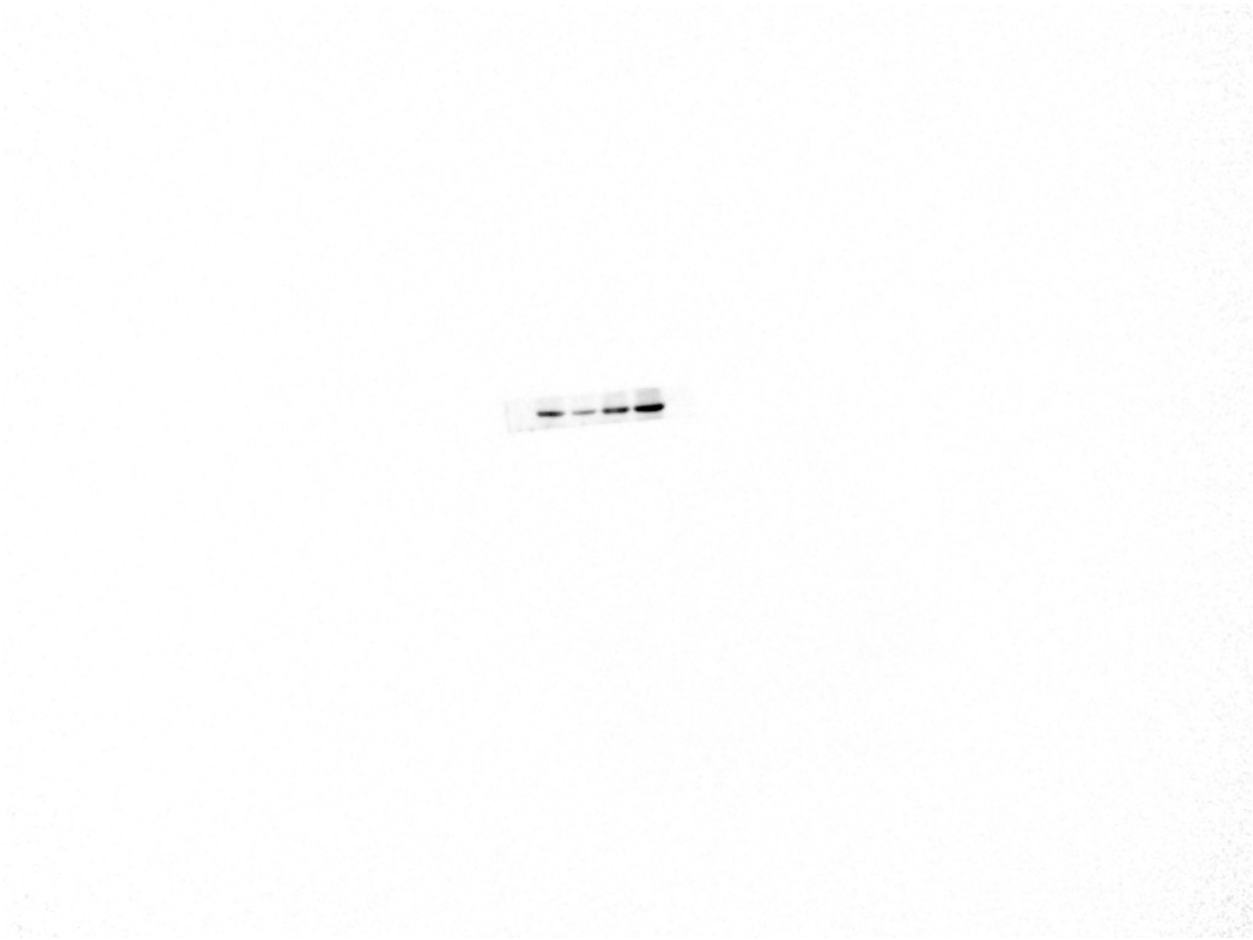

GAPDH

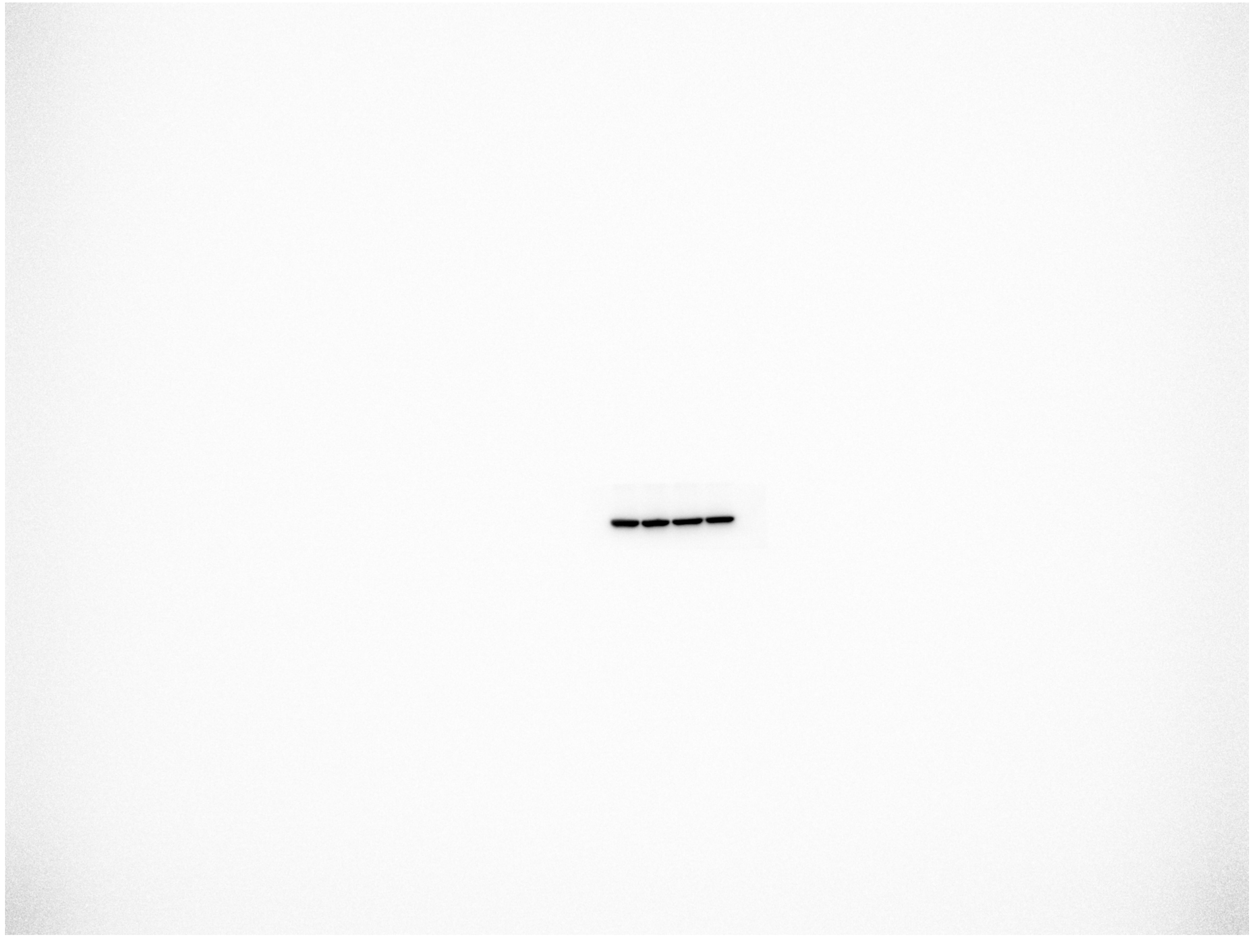

Figure3G

MGP

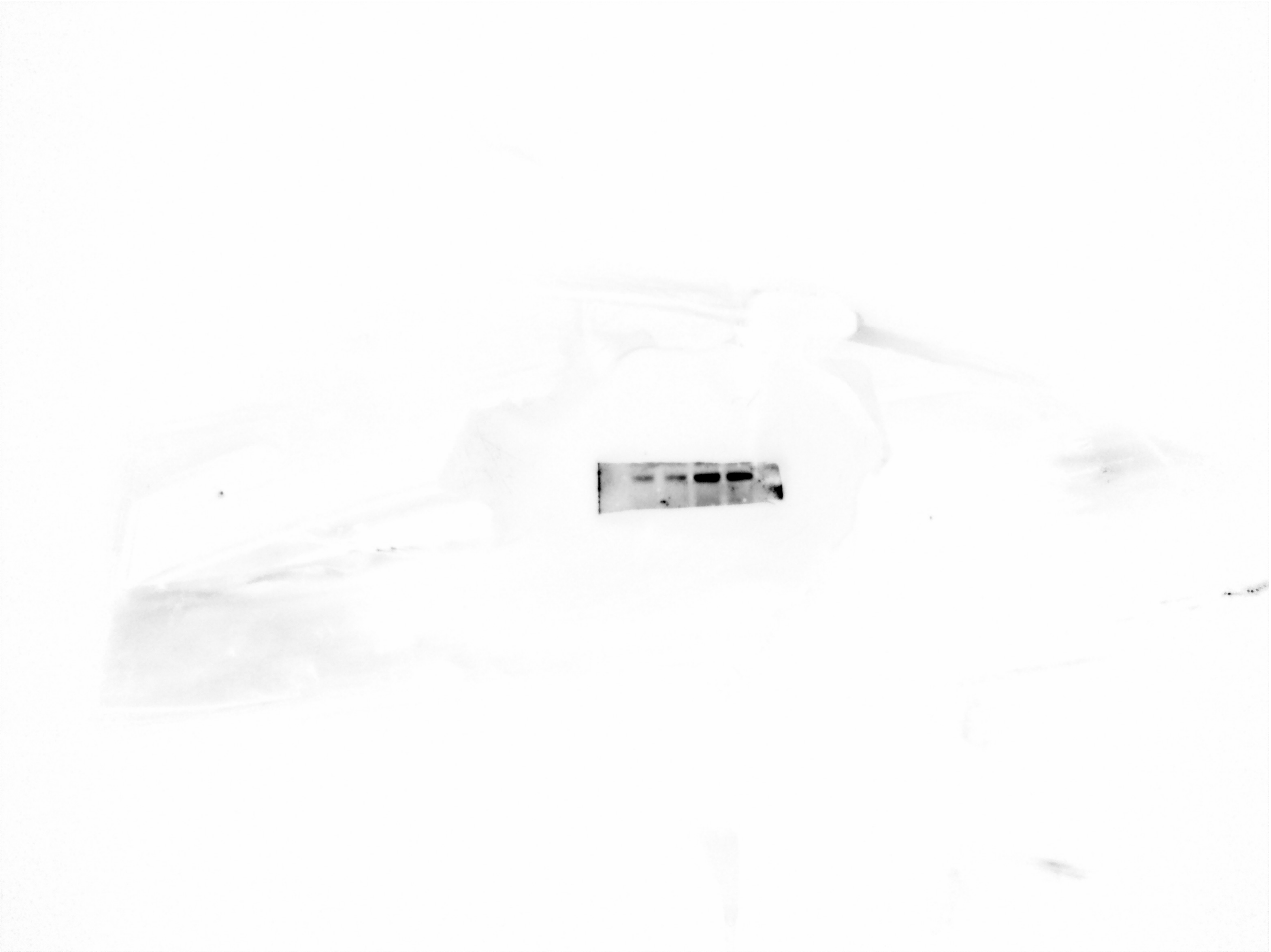

FABP3

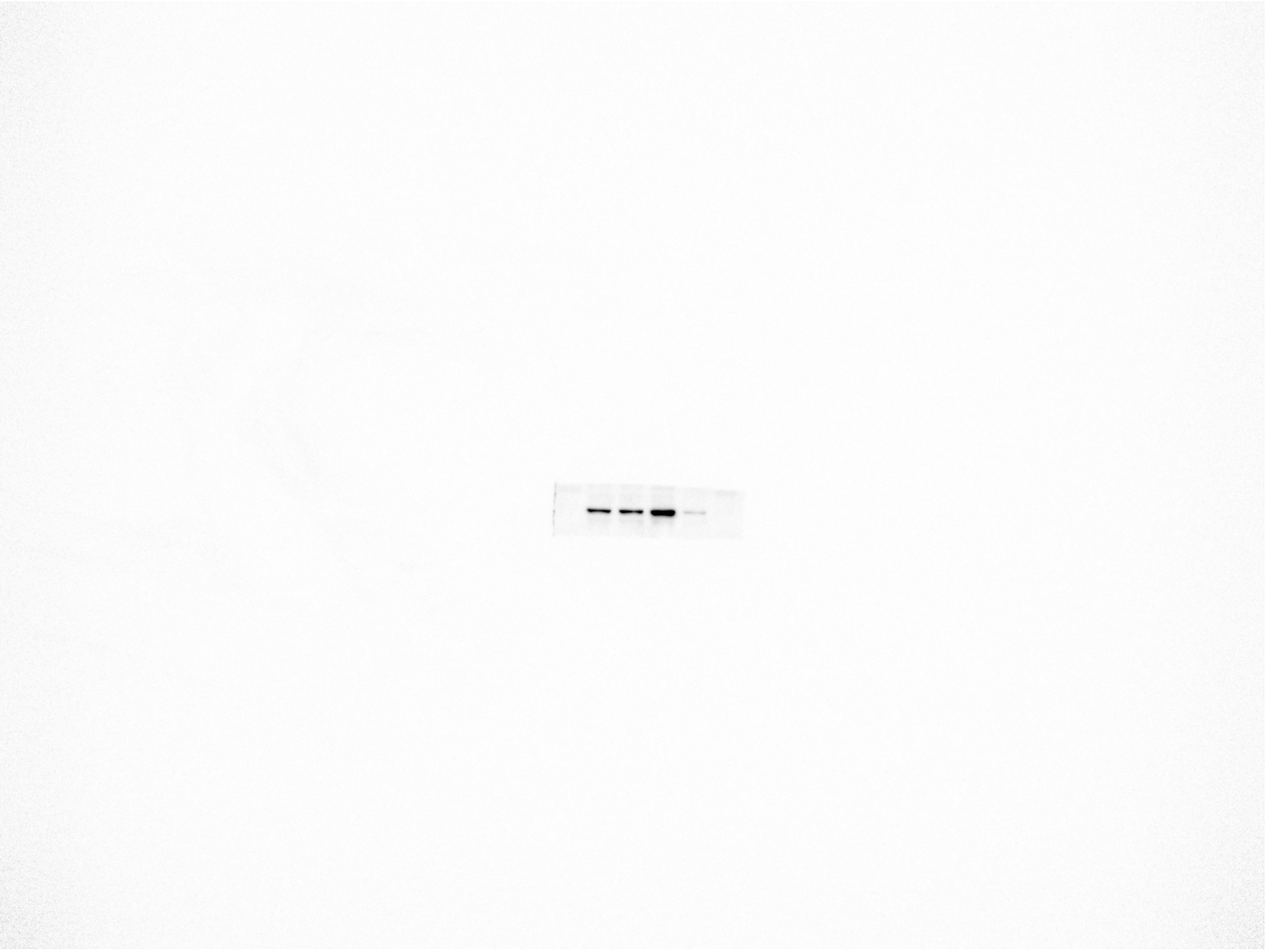

Figure3G

LPL

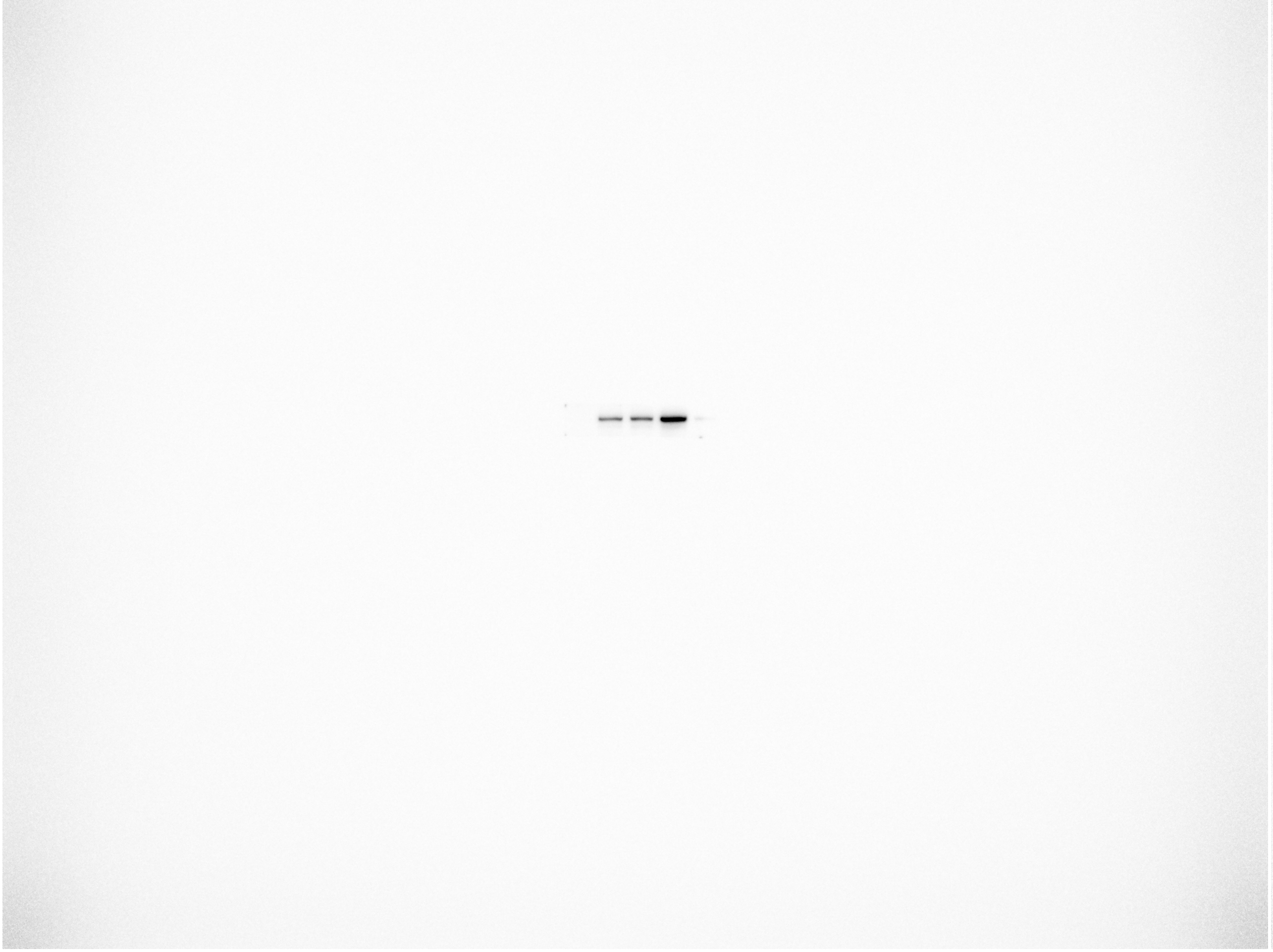

CEBP-α

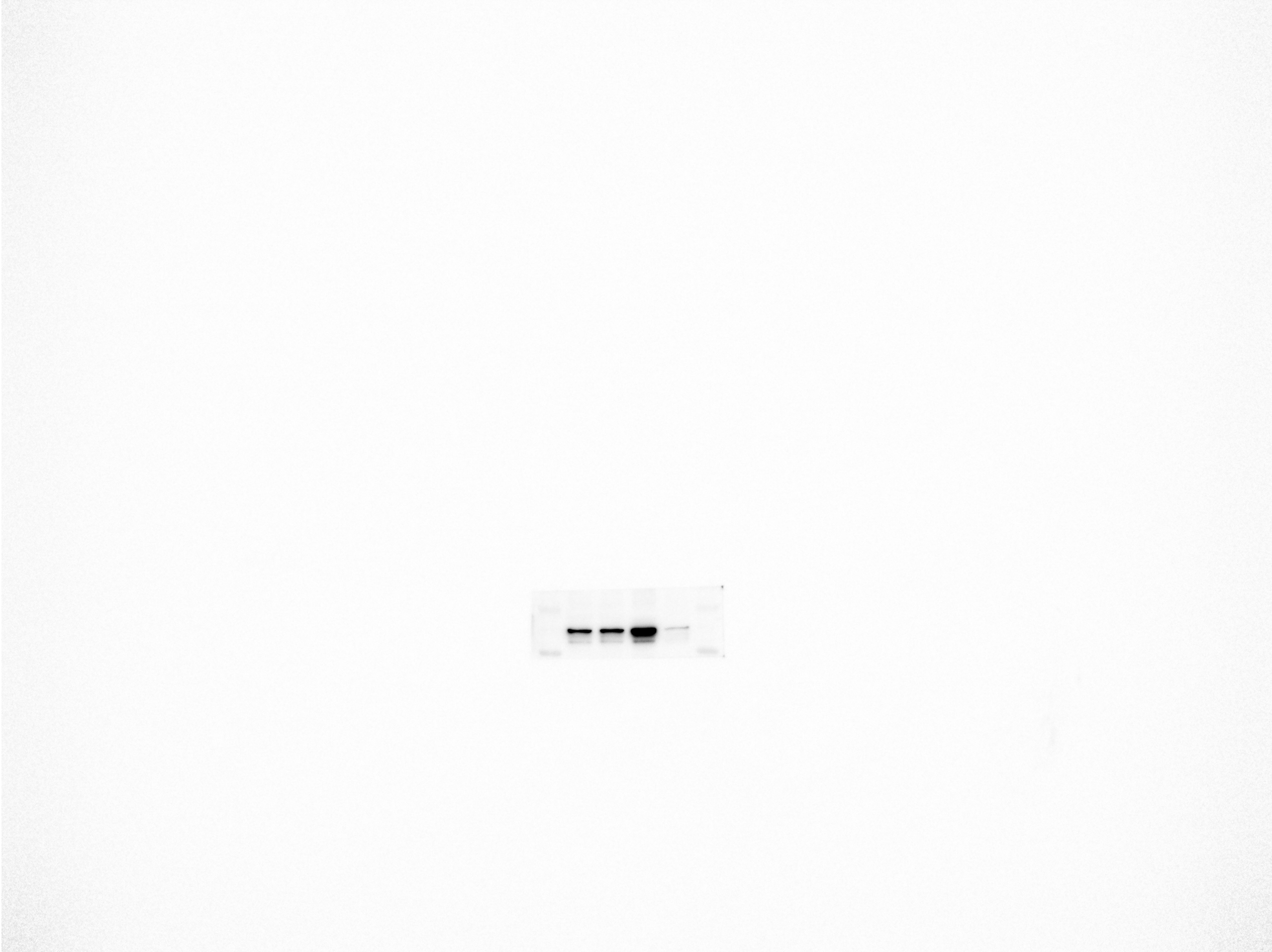

Figure3G

Perilipin1

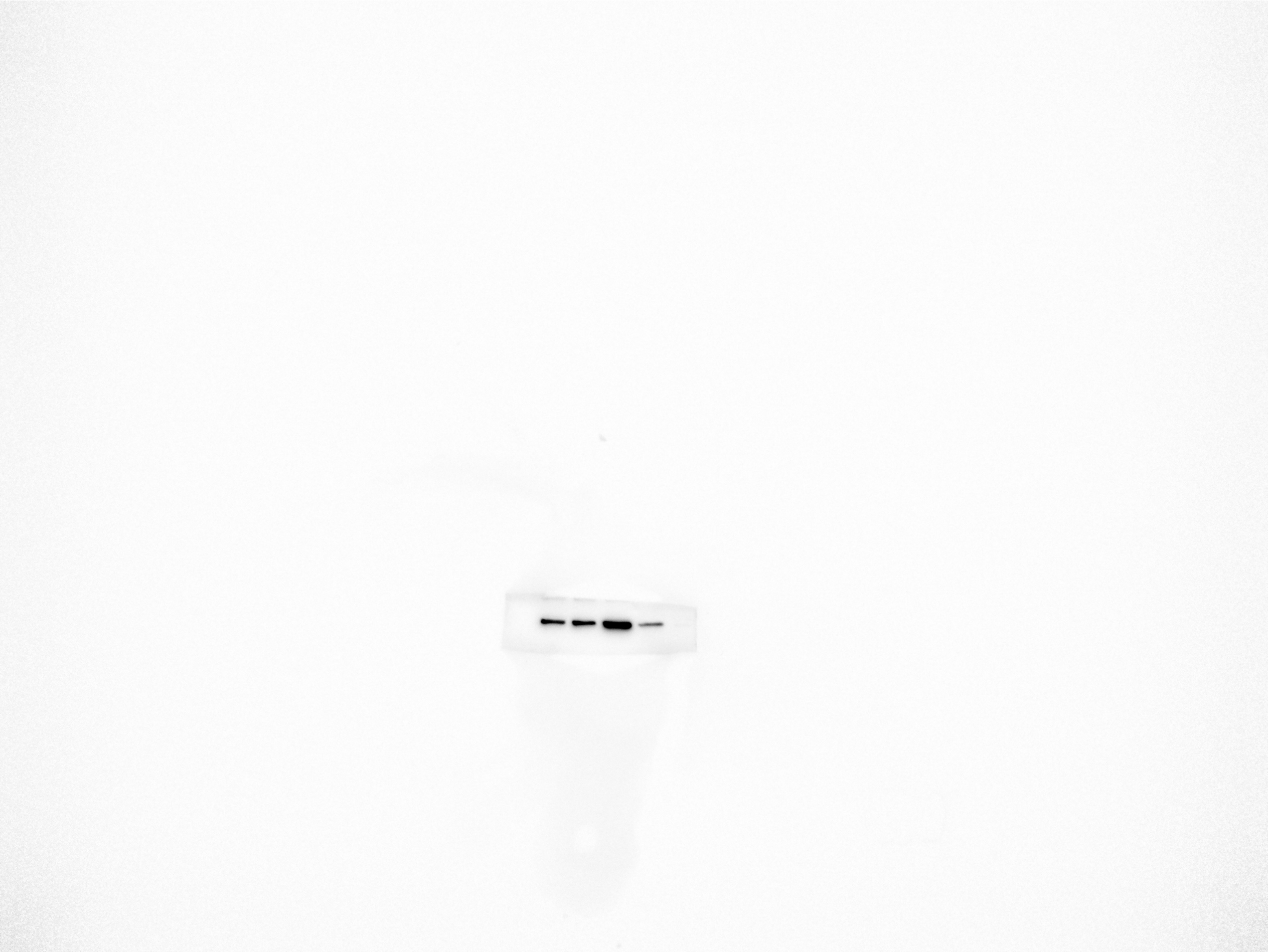

GAPDH

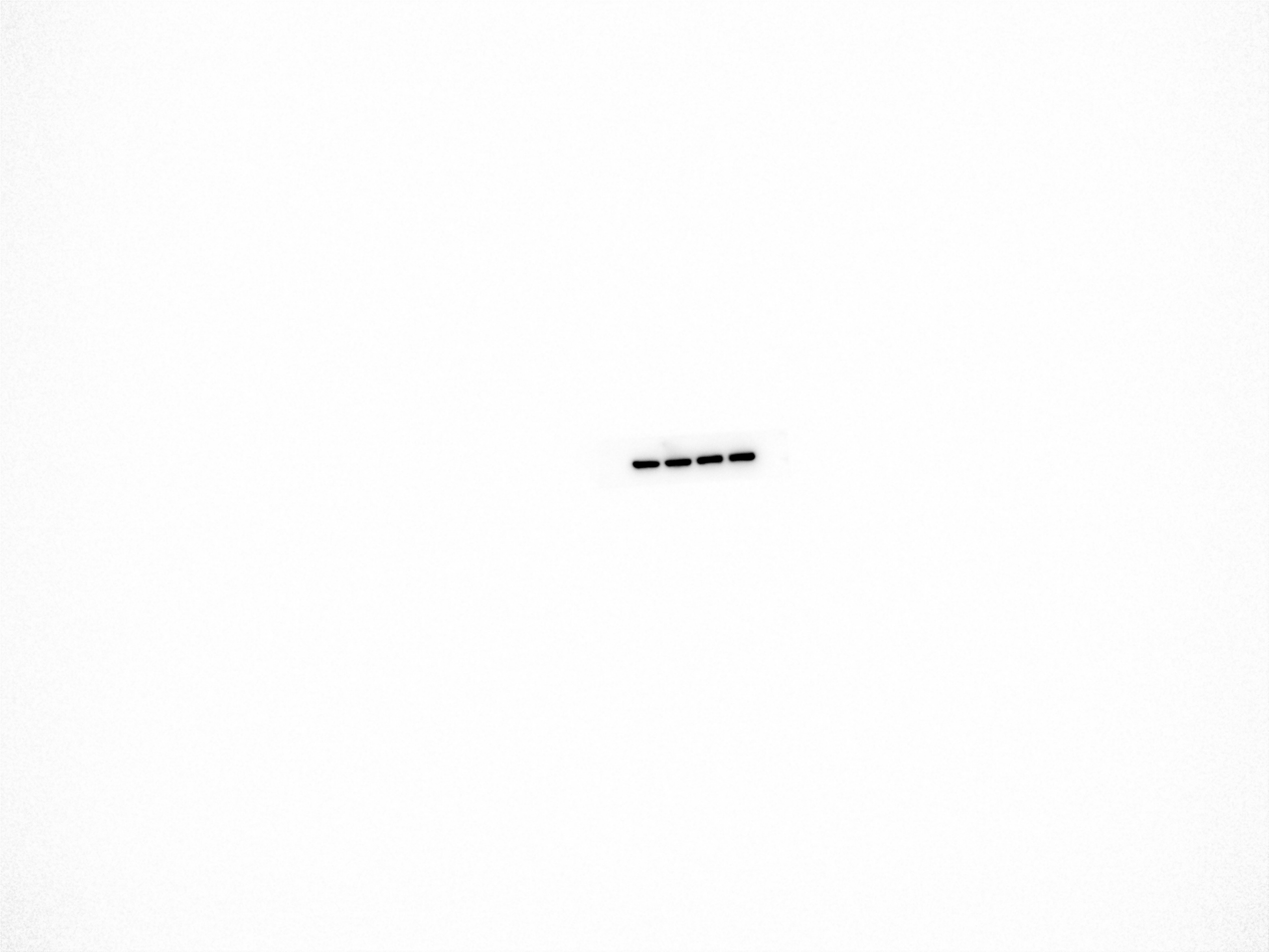

Figure4C

MGP

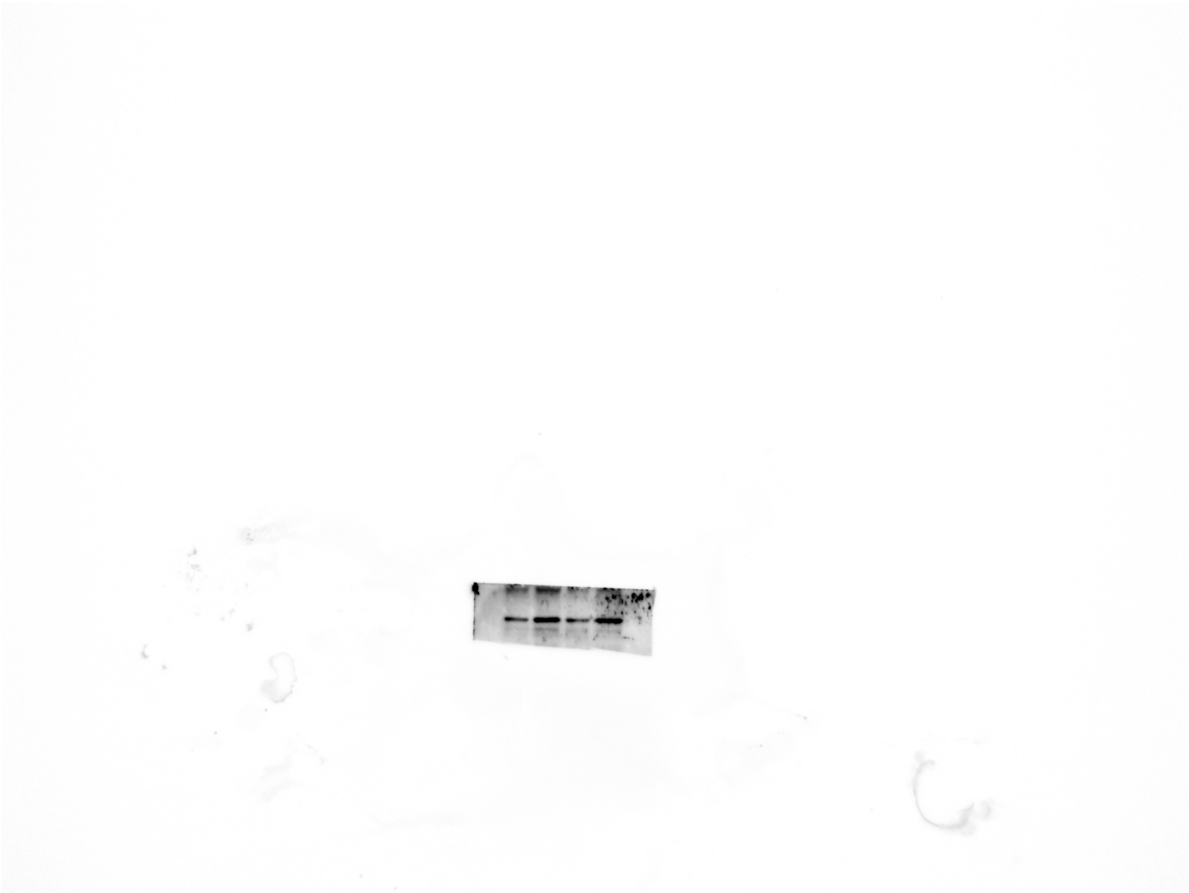

P-CaMKII

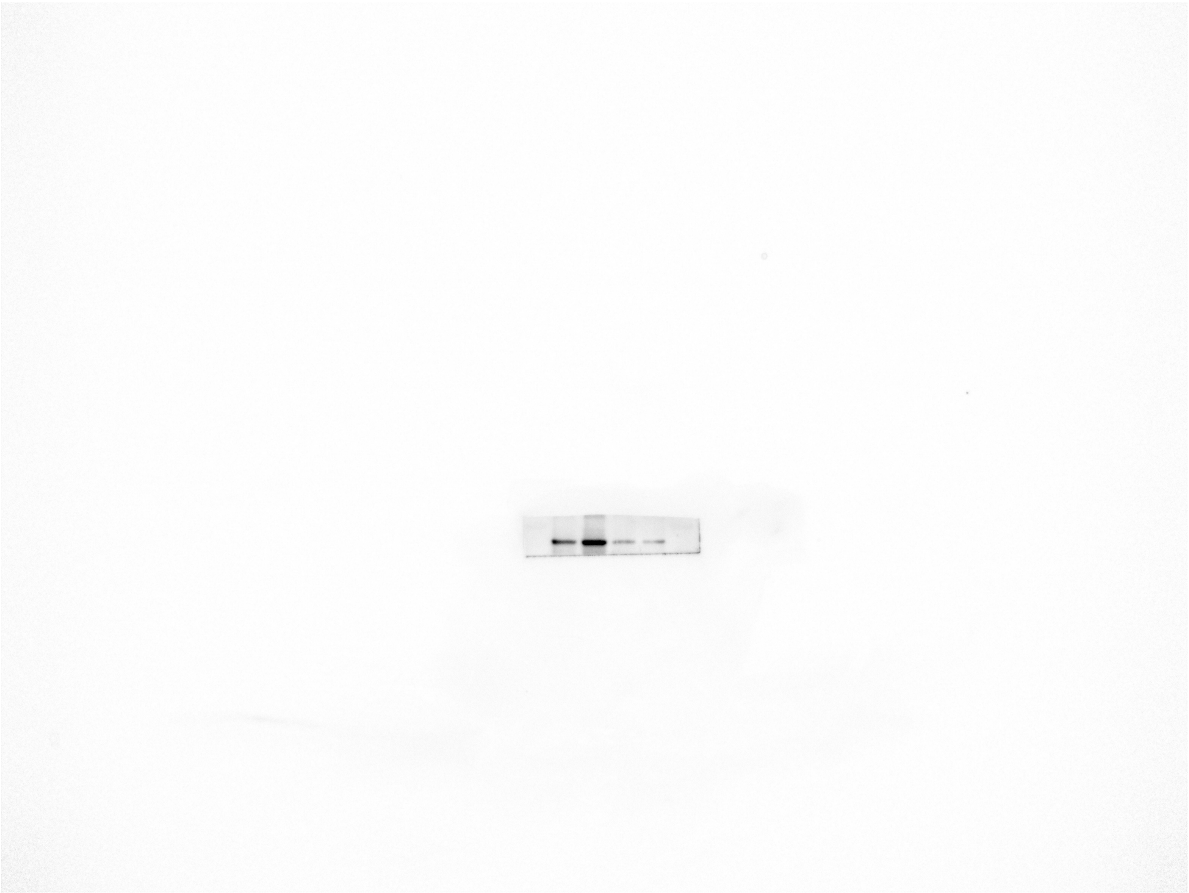

Figure4C

CaMKII

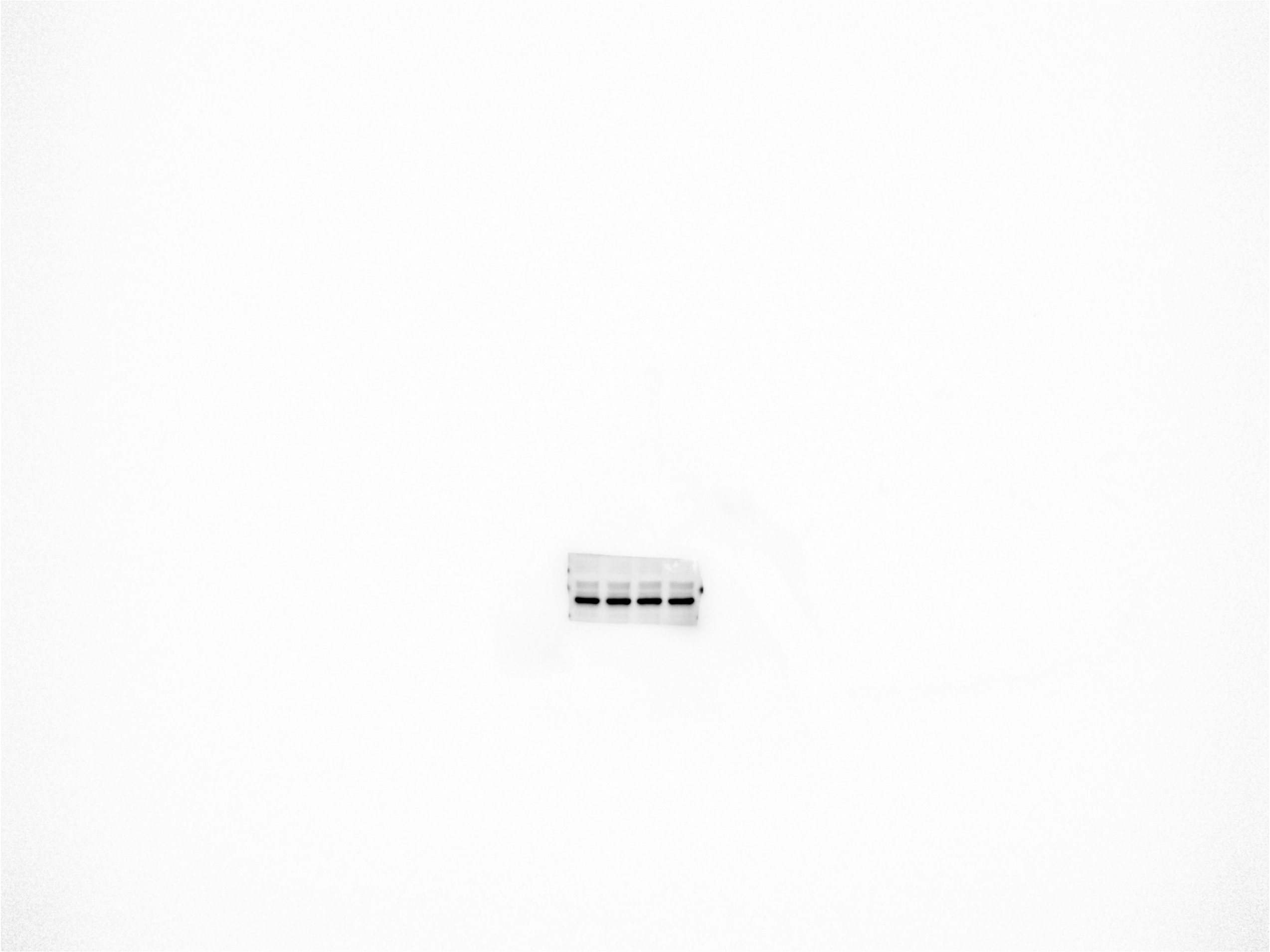

GAPDH

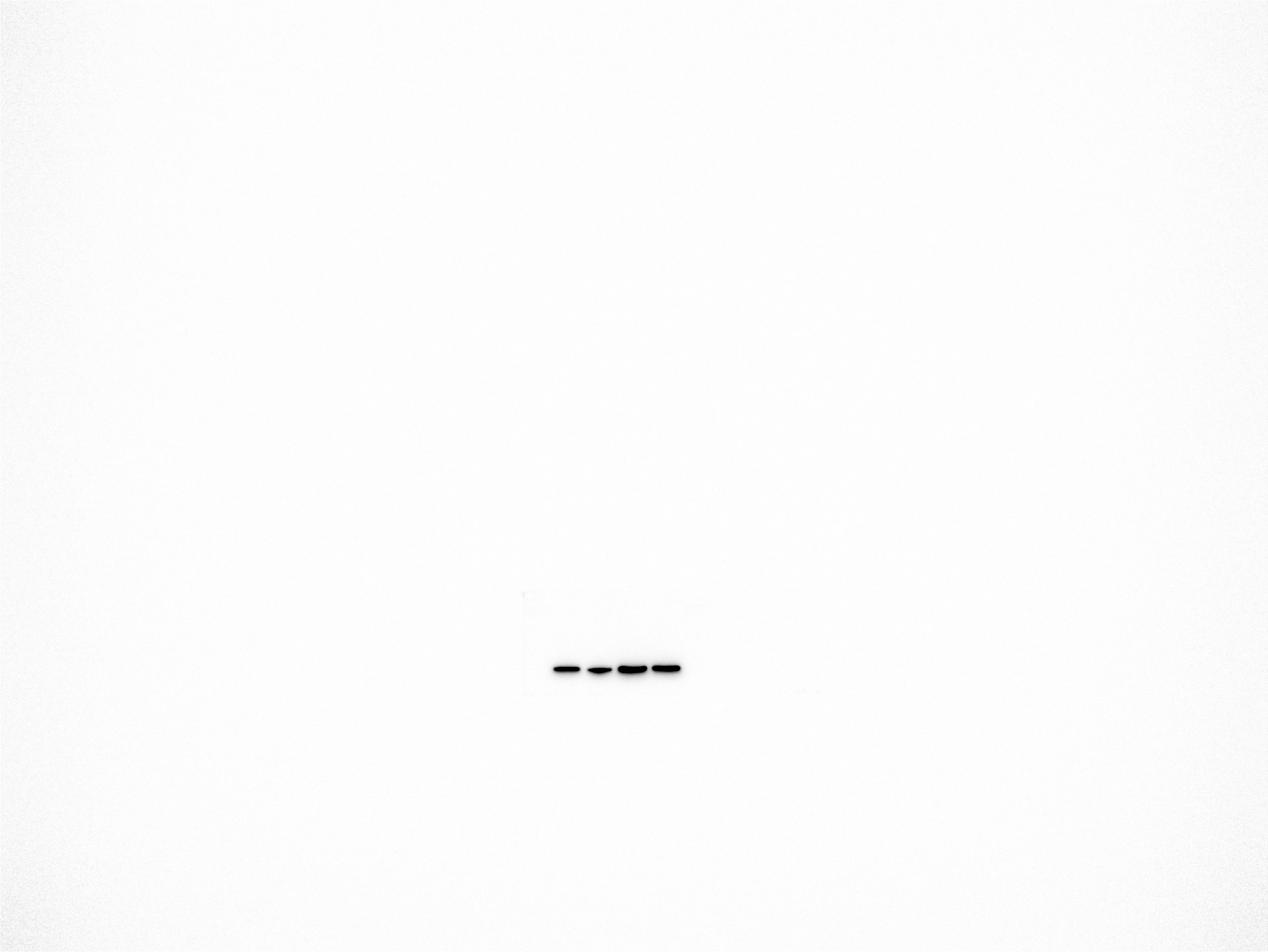

Figure5A

P-CaMKII

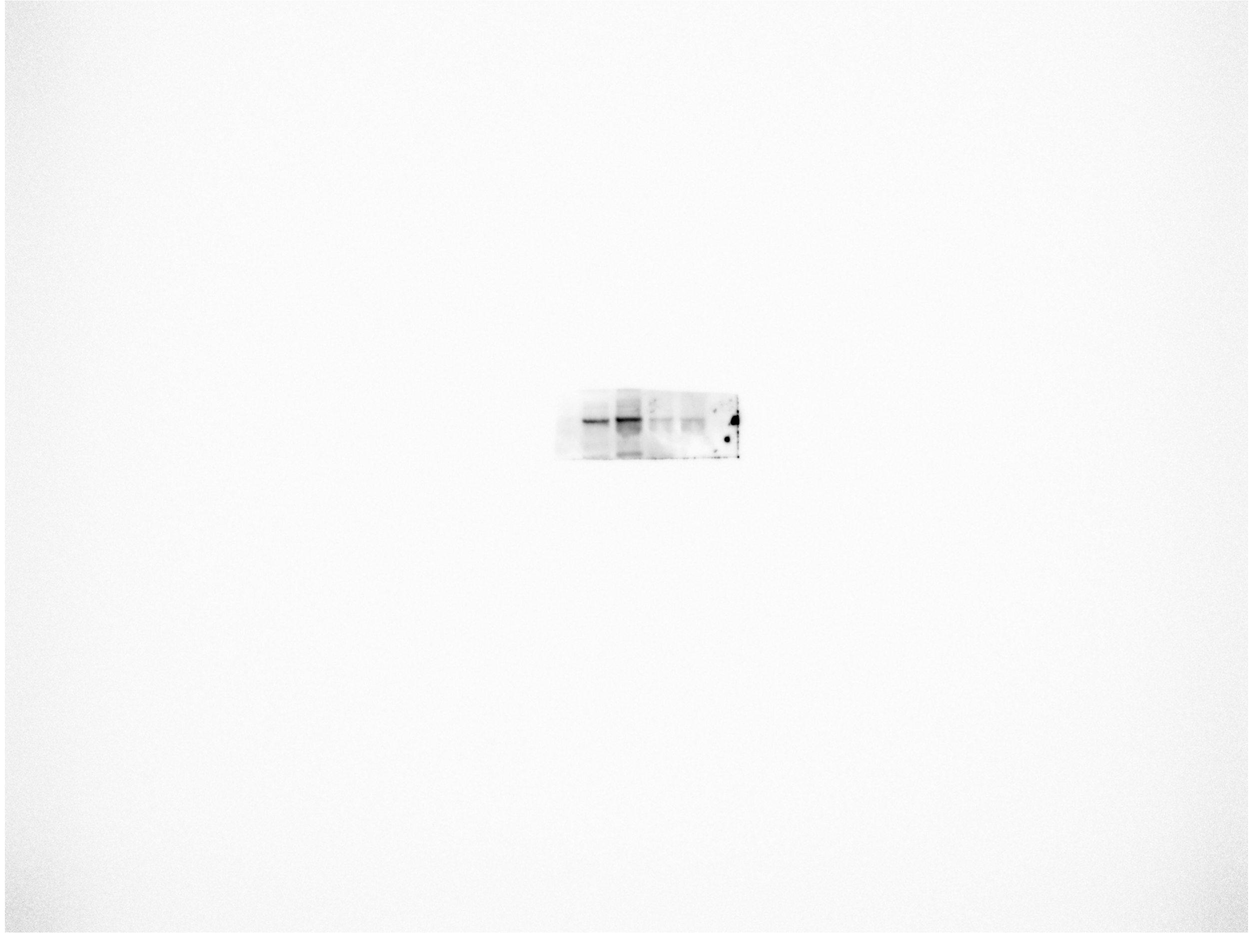

CaMKII

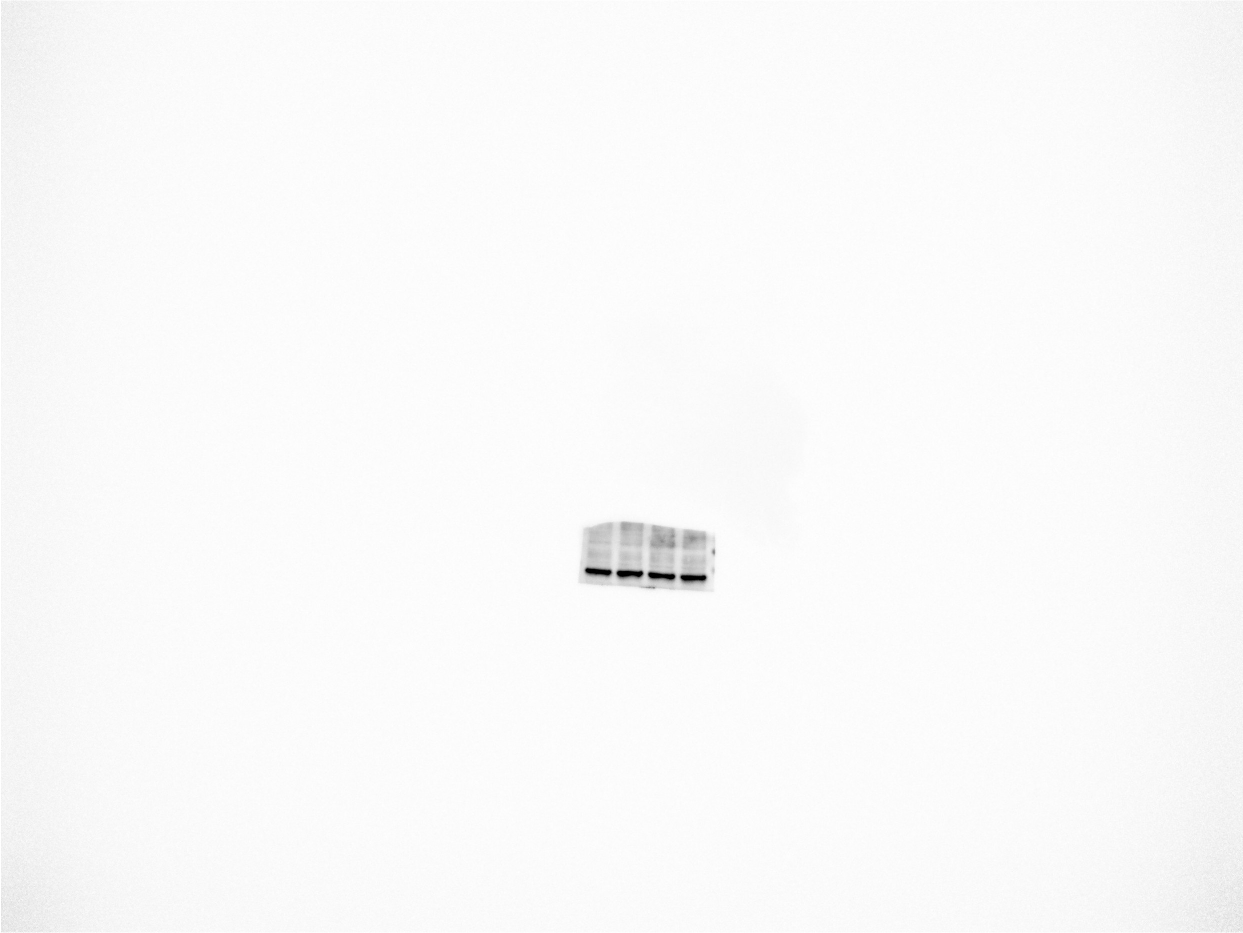

Figure5A

LPL **CEBP-α**

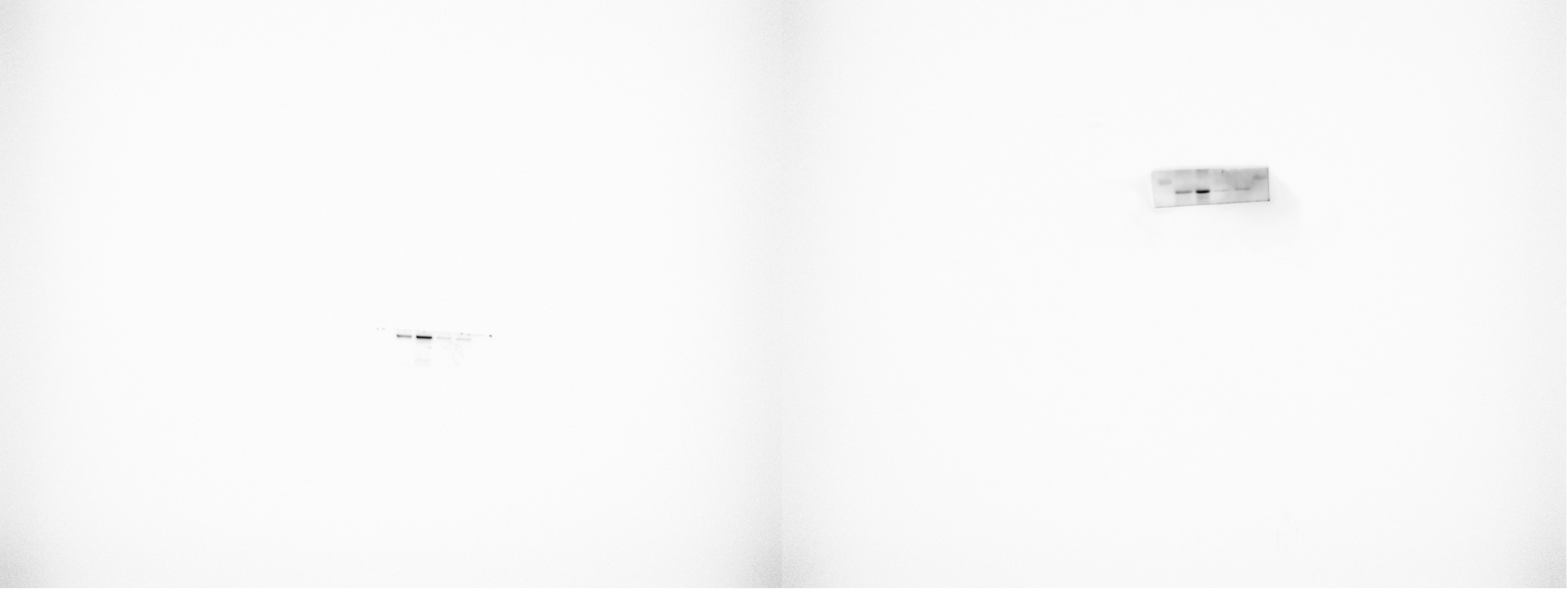

Figure5A

Perilipin1

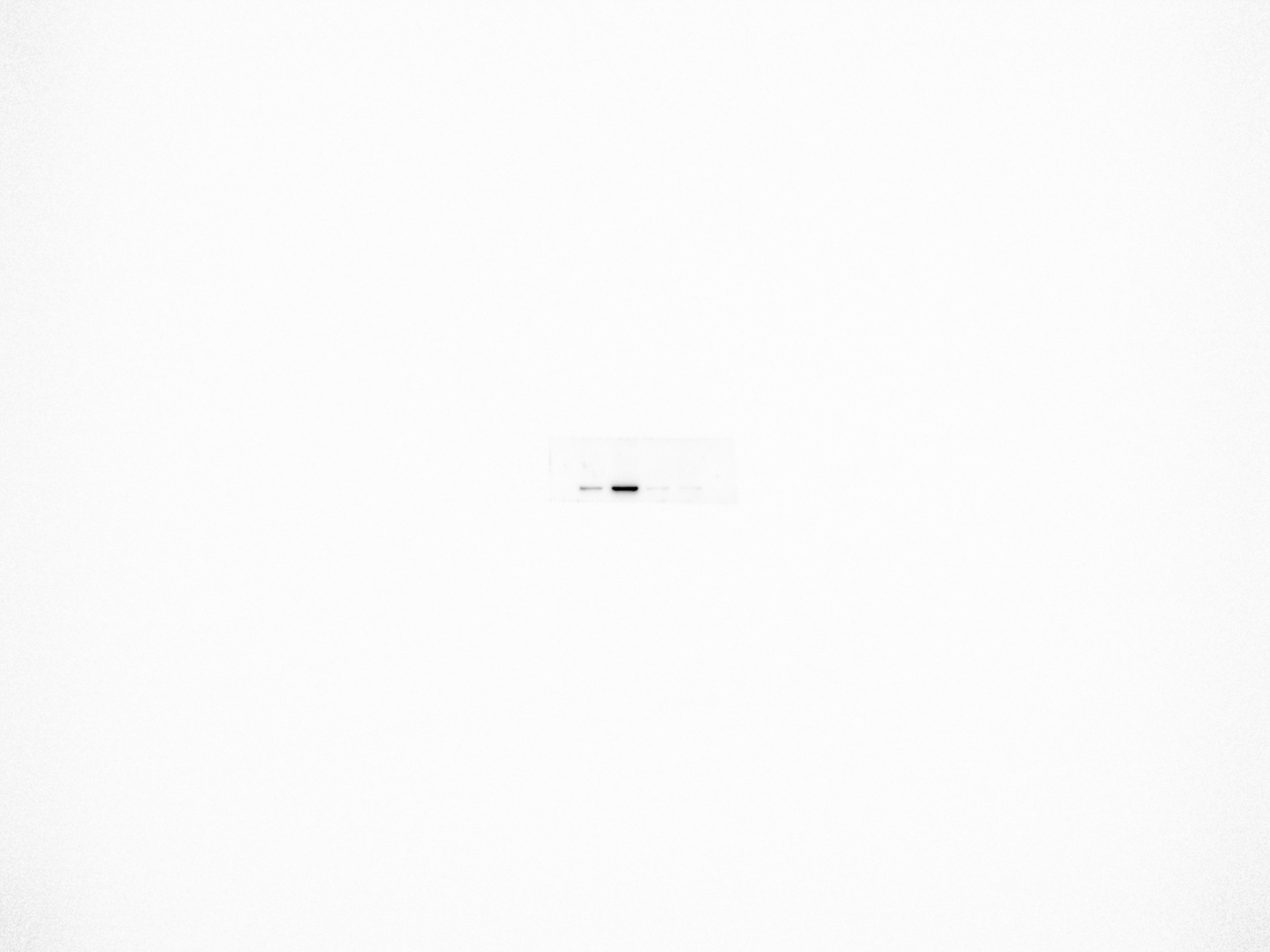

FABP3

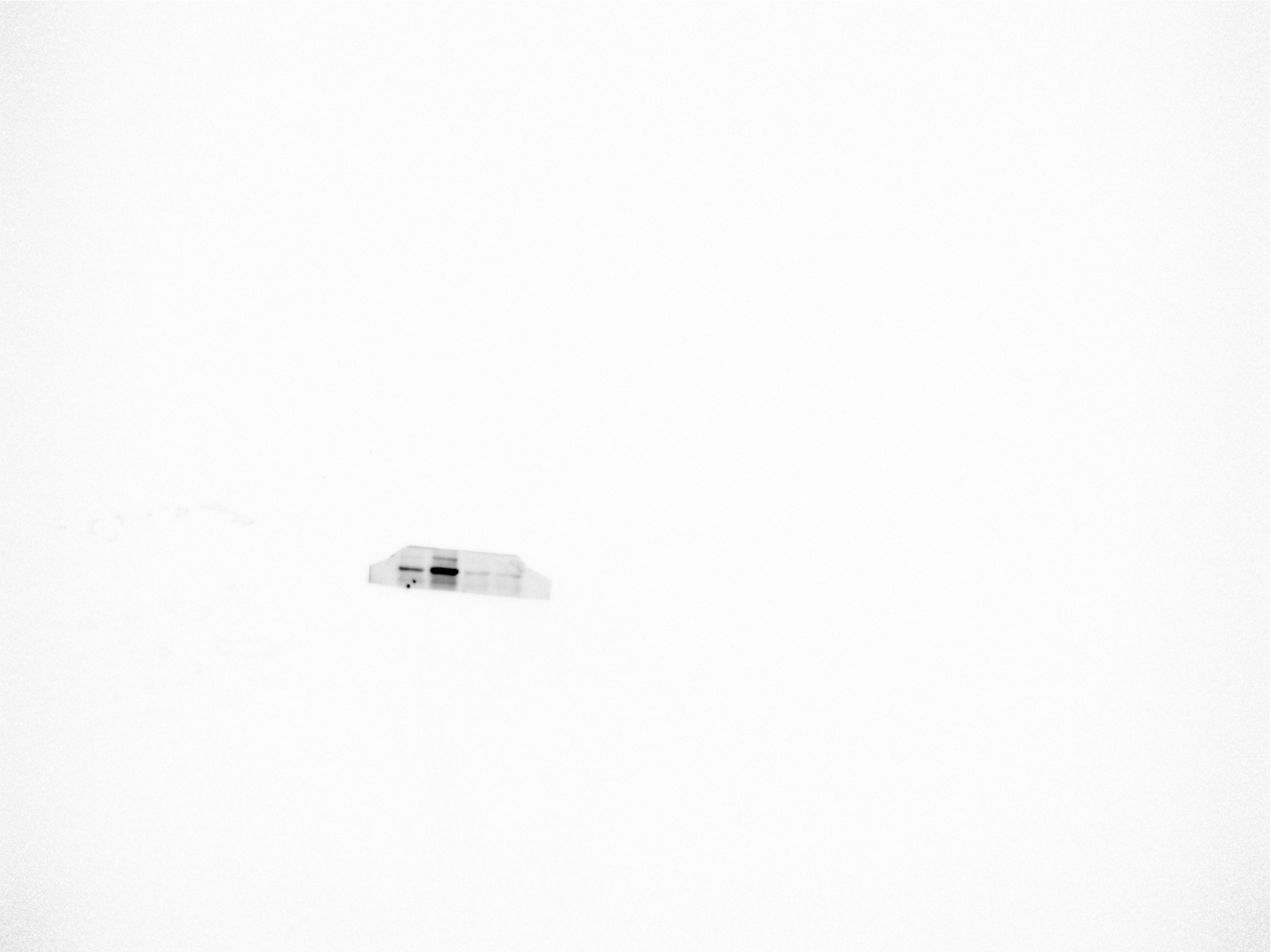

Figure5A

MGP

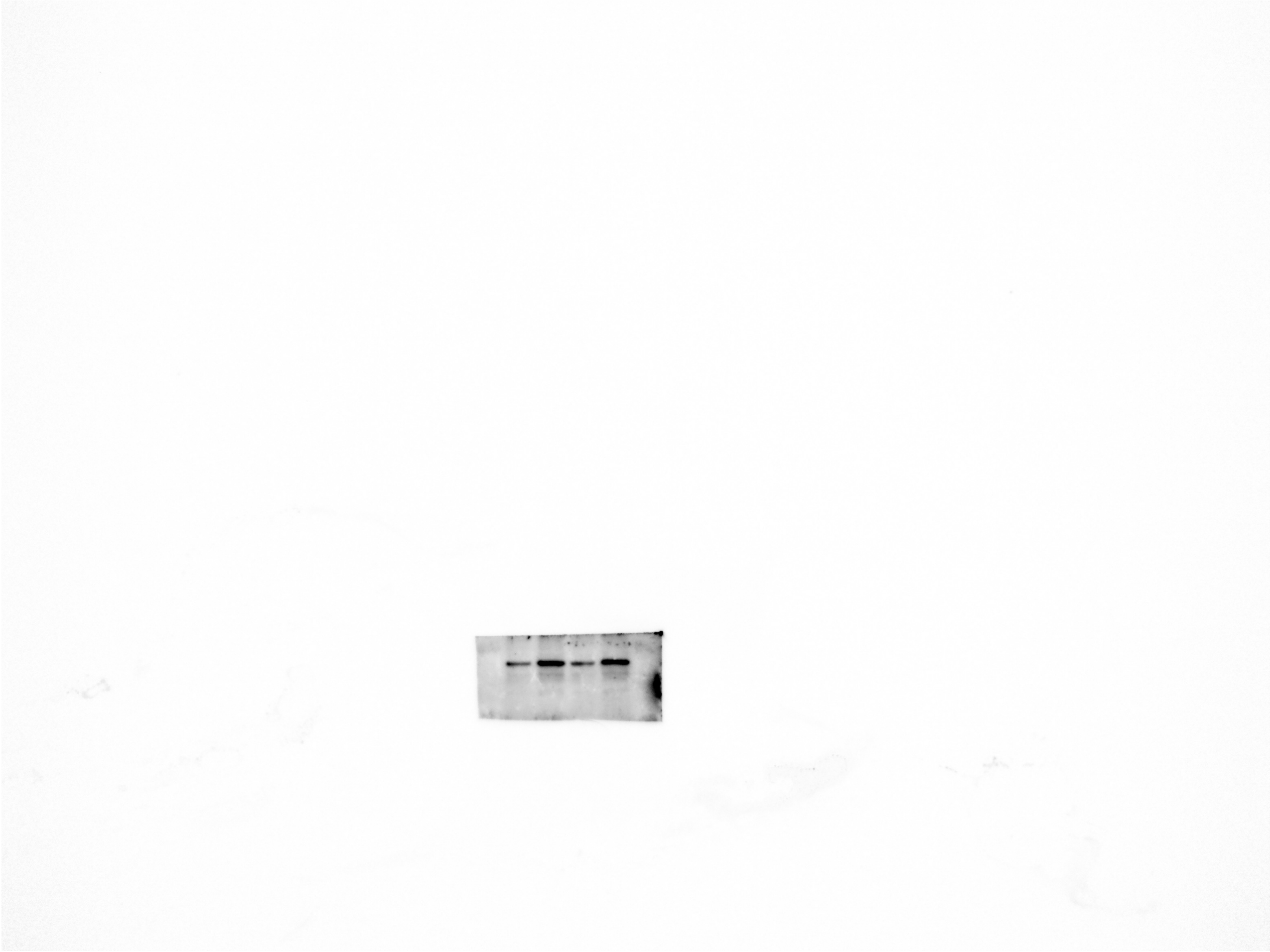

GAPDH

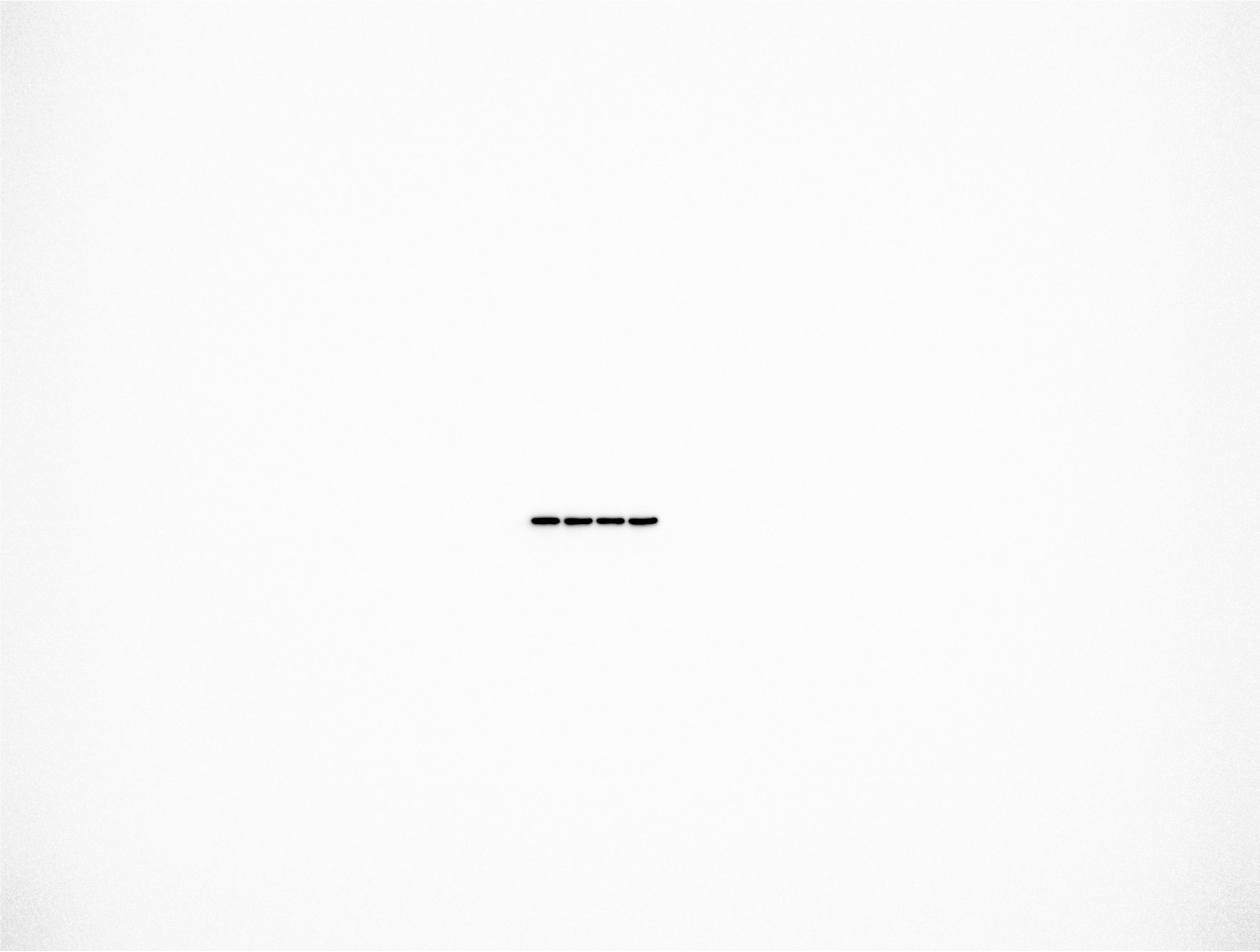

Figure5C

P-CaMKII

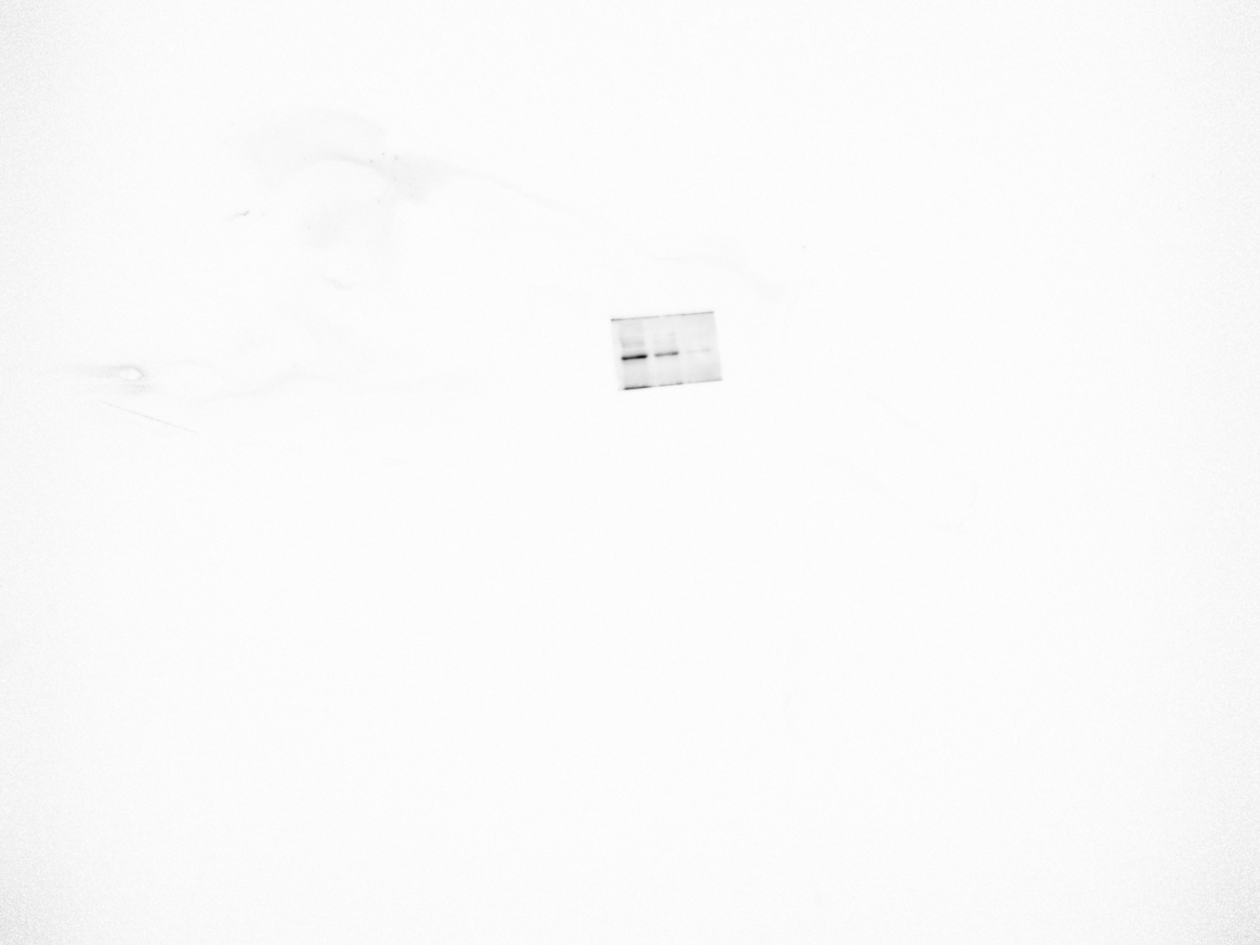

CaMKII

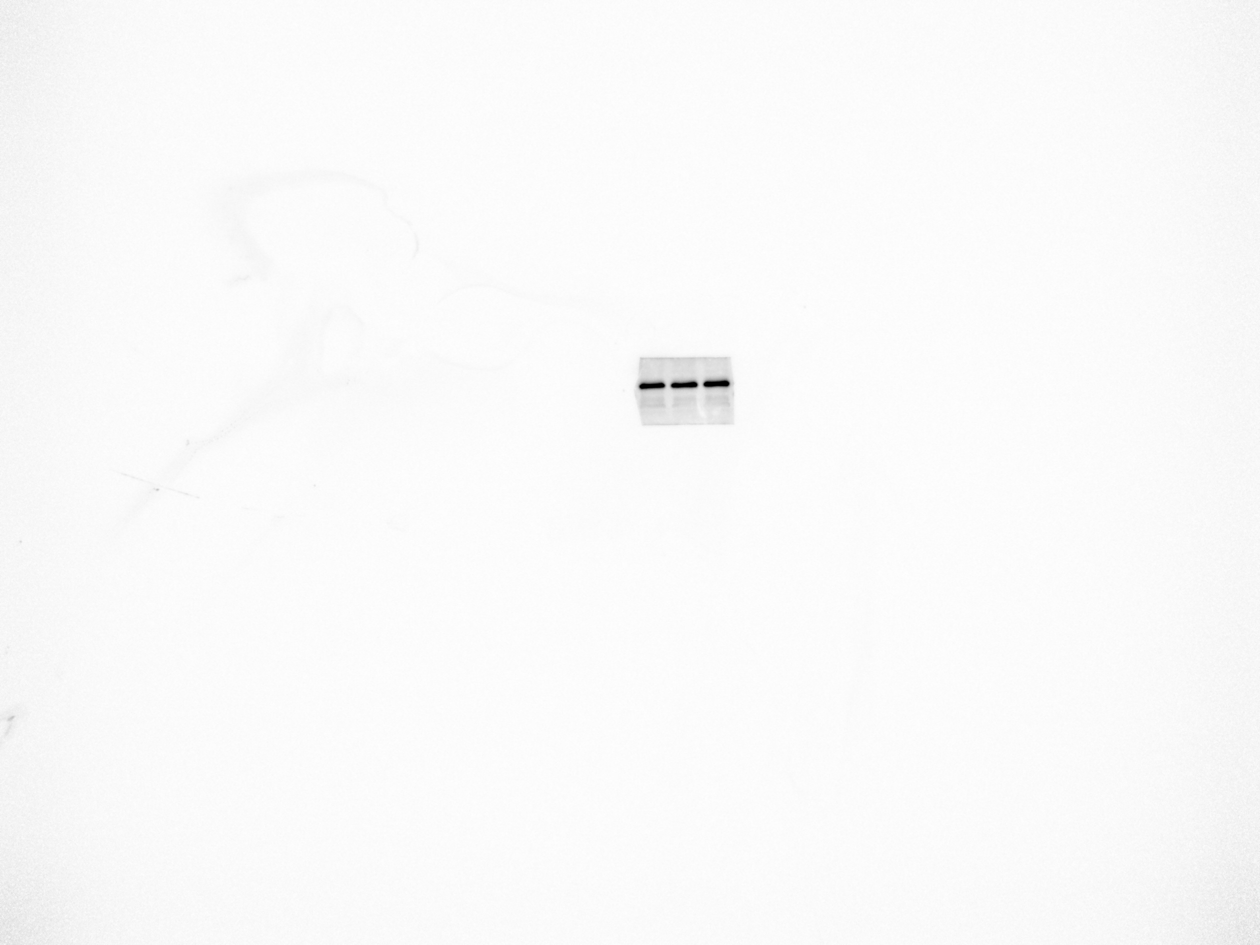

Figure5C

RIP140

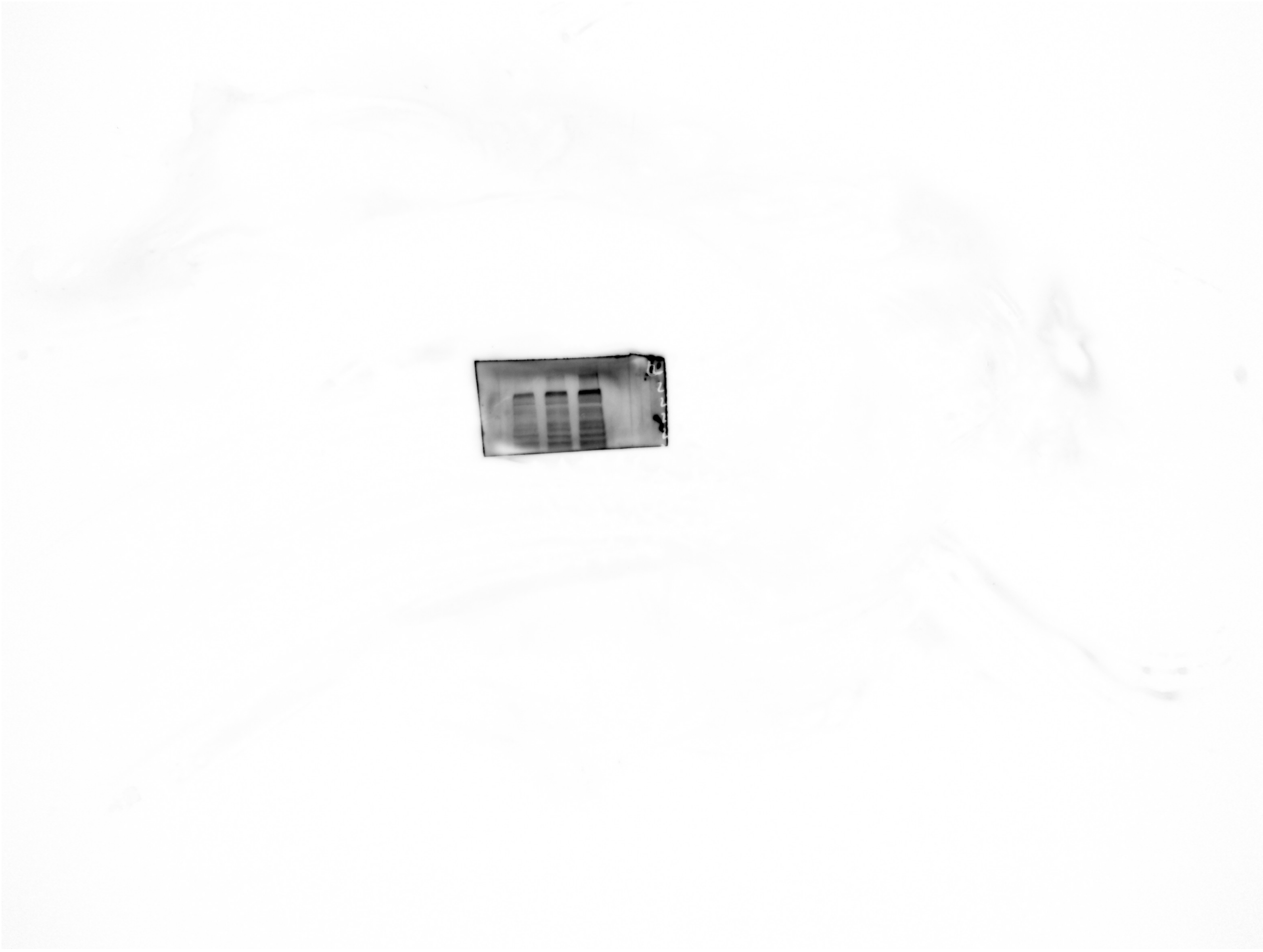

GAPDH

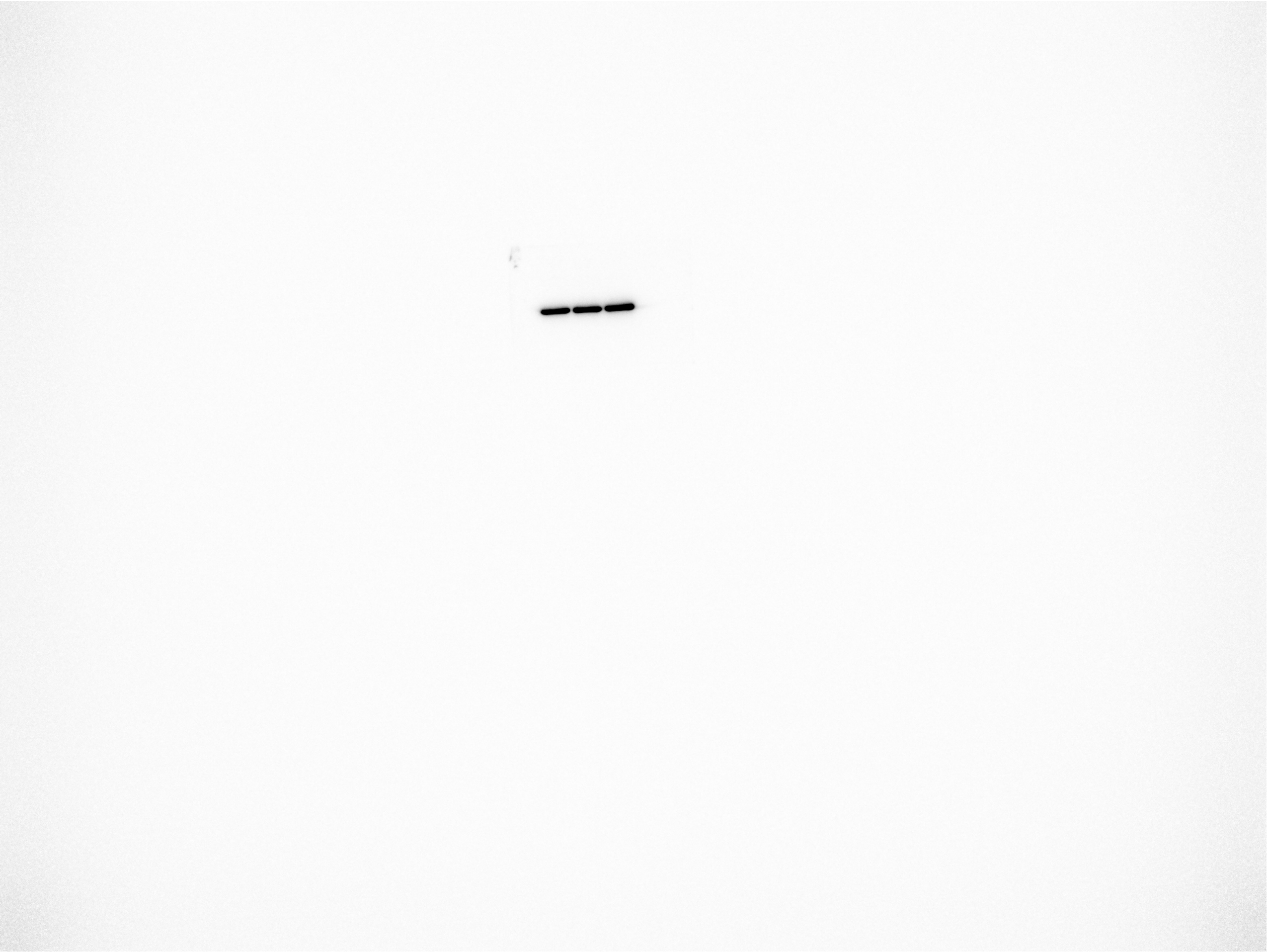

Supplement: Supplementary file 4 — Original western blots [file 41420_2025_2472_MOESM4_ESM.pdf]
